# Toward Near-Optimal and Scalable Placement and Orientation of Directional Sensors for Stochastic Directional Trajectory Detection

**DOI:** 10.3390/s26216955

**Published:** 2026-11-03

**Authors:** Mingyu Kim, Cholsa Kosal, Hyeongjun Mun, Heejip Moon, Jorge Jimenez

**Affiliations:** 1 Department of Electrical and Computer Engineering, Georgia Southern University, Statesboro, GA 30458, USA; 2 Department of Civil Engineering and Construction, Georgia Southern University, Statesboro, GA 30458, USA; ck17156@georgiasouthern.edu (C.K.); hm15582@georgiasouthern.edu (H.M.); 3 Bradley Department of Electrical and Computer Engineering, Virginia Tech, Blacksburg, VA 24061, USA; hmoon5@vt.edu; 4 Johns Hopkins University Applied Physics Laboratory, Laurel, MD 20723, USA; jorge.jimenez@jhuapl.edu

**Keywords:** directional sensor placement, stochastic trajectories, trajectory-level detection, log-Gaussian Cox process, sensor orientation optimization, void probability

## Abstract

Directional sensors are widely used to monitor moving targets, yet conventional placement methods often represent targets as spatial points or overlook the coupling among sensor location, orientation, and target travel direction. This paper develops a theoretically grounded and scalable framework for joint placement and orientation of directional sensors for stochastic directional-trajectory detection. Directed trajectories are represented by heading and signed perpendicular offset, preserving opposite travel directions along the same geometric path, and their stochastic occurrence is formulated through an intensity model that can be represented using a log-Gaussian Cox process. Sensor performance is evaluated at the trajectory level by incorporating directional field-of-view geometry, distance-dependent detection probability, sensing frequency, dwell time, and repeated sensing opportunities. Network performance is quantified through missed-trajectory intensity and a corresponding void-probability objective. To improve computational efficiency, we introduce a dwell-time-aware binary sensing surrogate with deployment-aware radius calibration and an omni-greedy algorithm that ranks locations using an omnidirectional surrogate before orientation refinement. Theoretical analysis provides bounds on sensing approximation error, greedy decision preservation, location–orientation approximation, and end-to-end deployment performance. Experiments using real maritime AIS and wildlife GPS trajectories show that omni-greedy reduces selection-stage candidate evaluations by roughly 18–21× while maintaining detection performance close to full directional greedy optimization; when candidate responses are cached, total planning time is dominated by shared precomputation and radius calibration.

## 1. Introduction

Directional sensors support monitoring applications in which detection depends on both sensor position and viewing direction, including maritime surveillance and wildlife monitoring. For moving targets, effective deployment also depends on where targets travel, how long they remain within a sensor’s field of view, and the detection opportunities available during each passage. A configuration that covers a large spatial area may therefore provide limited detection of the trajectories that occur most frequently. This motivates a deployment objective based on the probability of detecting complete target passages, weighted by their occurrence intensity.

This paper addresses the joint selection of sensor locations and orientations for detecting stochastic target trajectories under a prescribed sensor budget. Candidate locations and orientations are drawn from finite sets, with at most one orientation selected at each location. Each target passage is associated with a directed path and the motion information needed to determine its exposure to the sensors. The objective is to maximize the expected number of detected passages over a specified observation window. This formulation connects the spatial distribution of target traffic with directional sensing, repeated detection opportunities, and overlap among deployed sensors.

Two computational difficulties arise. First, evaluating trajectory-level detection requires accumulating probabilistic sensing opportunities along each passage. Repeating this calculation across many candidate configurations can be expensive. Second, a joint greedy search must compare combinations of location and orientation at every deployment step. The resulting computational cost grows with the number of candidate locations, orientations, and trajectory samples. Reducing either cost introduces an approximation whose effect on deployment quality must be assessed.

We address these difficulties through a dwell-time-aware binary sensing approximation and an omni-greedy deployment strategy. The binary model represents a passage as detected when its residence time within a radius-limited directional footprint satisfies a prescribed threshold. An error analysis relates this approximation to the underlying probabilistic detection model and supports the selection of the binary sensing radius. The omni-greedy strategy then separates each deployment decision into two stages: it ranks available locations using an omnidirectional proxy and selects an orientation at the chosen location using the directional objective. The proxy is used only for location selection; every sensor in the resulting deployment remains directional.

The intended improvements are therefore specific. At the modeling level, the formulation evaluates detection over target passages while accounting for directional coverage and sensing exposure. At the computational level, the proposed selection strategy reduces the number of candidate-score evaluations relative to jointly evaluating all available location–orientation pairs at every greedy iteration. At the analytical level, the framework distinguishes the error caused by the binary sensing approximation from the error caused by separating location and orientation selection. This distinction makes it possible to examine the conditions under which computational savings preserve deployment quality and the conditions under which the approximations may become inaccurate.

The main contributions are as follows:

1. A trajectory-level deployment formulation: We formulate joint placement and orientation selection using a trajectory-occurrence intensity and accumulated probabilistic detection. The formulation explicitly connects individual sensing opportunities, passage-level detection, and the network objective.
2. An error-controlled binary sensing approximation: We develop a dwell-time-aware binary model and analyze its effect on the deployment objective and marginal gains. The resulting bounds support radius calibration and provide sufficient conditions for preserving greedy decisions.
3. A scalable deployment strategy with explicit approximation analysis: We develop an omni-greedy procedure that selects location before orientation, reducing candidate-score evaluations. We analyze the additional approximation introduced by this separation and relate the deployment procedure to the submodular structure of the finite candidate problem.
4. An evaluation using maritime and wildlife trajectories: We examine detection performance, approximation behavior, and computational requirements using two application datasets. Comparisons with joint directional greedy selection and selection using the probabilistic sensing model assess the effects of the proposed approximations.

The theoretical guarantees are stated relative to the finite candidate deployment problem under the specified sensing and trajectory assumptions. They do not establish unrestricted global optimality over continuous locations and orientations. Likewise, the empirical evaluation examines performance on the datasets and sensing models considered, with the approximation bounds providing a means to interpret the observed tradeoffs.

The remainder of this paper is organized as follows. Section 2 reviews related work and identifies the distinctions between existing approaches and the proposed framework. Section 3 presents the trajectory and sensing models, deployment objective, and algorithms. Section 4 develops the theoretical analysis. Section 5 reports the experimental setup and results. Section 6 discusses the findings and limitations, and Section 7 concludes the paper.

## 2. Related Work

Research relevant to this study includes directional coverage optimization, sensor deployment for moving targets, and stochastic sensor placement. These research areas address related sensing decisions but differ in their objectives, treatment of target motion, and deployment constraints. The following review identifies these distinctions and positions the present framework within the existing literature.

### 2.1. Directional Coverage and Moving-Target Monitoring

Directional coverage optimization accounts for the dependence of a sensor’s footprint on its orientation. Fusco and Gupta [1] studied sensor selection and orientation for geometric coverage maximization, establishing that orientation must be considered explicitly when selecting directional sensors. Such formulations provide a foundation for deployment optimization, although geometric coverage and the probability of detecting a complete target passage are different performance criteria.

Moving-target detection has also been considered directly in directional sensor deployment. Wasim et al. [2] developed a time-phased deployment strategy using probabilistic target-motion models and direct-search optimization. Their formulation incorporates deployment timing and sensor deliverability constraints when planning directional sensors for mobile-target search. Consequently, neither directional sensing nor the use of target-motion information alone distinguishes the present study. Our emphasis is on a passage-level detection objective and the explicit analysis of approximations used to simplify its evaluation and optimize deployment.

Another research direction concerns the online coordination of directional sensors. Xu et al. [3] proposed hierarchical target-oriented multi-agent coordination, in which a coordinator assigns targets and individual executors adjust their sensing directions to track the assigned targets. Pan et al. [4] introduced the Multi-Agent Tracking Environment (MATE), a benchmark for distributed target coverage involving cooperative camera agents and mobile target agents. These studies address sequential sensing control and coordination. The present work instead selects a fixed deployment from candidate locations and orientations using a statistical description of target passages.

More recently, Kósa et al. [5] combined moving-target coverage with the maintenance of *k*-barrier coverage. For cameras at given locations, they developed an integer linear programming formulation, clustering strategies, and a greedy alternative for selecting sensing sectors. Their formulation explicitly couples target observation with a geometric barrier requirement. Our formulation instead optimizes intensity-weighted passage detection under a sensor budget; it does not impose an uninterrupted *k*-barrier coverage constraint.

### 2.2. Stochastic Sensor Placement and Detection Objectives

Stochastic placement methods account for the spatial variability and uncertainty of target occurrence. Kim et al. [6] considered sensor placement for detecting Poisson-distributed targets and used the probability of missing no targets as a network performance criterion. They introduced a monotone submodular approximation that supports greedy placement and analyzed the discrepancy from the original objective. Subsequent work [7] developed an improved approximation of void probability for seabed acoustic sensor networks, with greater attention to uncertainty in the target intensity model.

Trajectory-based stochastic placement provides a more direct connection to the present problem. Kim et al. [8] represented linear target trajectories as points in a transformed space and considered sensor placement for a barrier coverage system under a log-Gaussian Cox line-process model. This representation connects target traffic patterns with the probability of detecting passing targets. The present study builds on this trajectory-space perspective while addressing joint location and orientation selection, accumulated sensing opportunities along finite passages, and a dwell-time-aware binary approximation.

The distinction between occurrence uncertainty and sensing approximation is important. Approximating a network objective under uncertain target intensity is a different operation from replacing probabilistic passage detection with a binary sensing model. Likewise, separating location and orientation selection introduces an additional approximation. The present analysis therefore treats binary sensing error and location–orientation decoupling error separately. In the experimental implementation, the estimated trajectory intensity supplies the weights of the deployment objective; this use of an estimated intensity should not be interpreted as fitting a full log-Gaussian Cox posterior.

### 2.3. Comparison and Scope of the Present Study

Table 1 summarizes representative formulations and their relationship to the present study. Existing research already addresses directional sensor selection, probabilistic moving-target search, online camera coordination, and stochastic trajectory-based placement. The contribution pursued here is the integration of passage-level probabilistic detection, dwell-time-based approximation, and a location-first, orientation-second deployment procedure with separate analyses of the resulting approximation errors.

The omnidirectional footprint in the proposed procedure serves only as a location-ranking proxy. Orientation is subsequently selected using the directional objective, and the deployed sensors remain directional. This procedure reduces the number of candidate-score evaluations relative to joint enumeration of available location–orientation pairs at each greedy iteration. Its performance guarantees concern the stated finite candidate problem and assumptions; they do not establish unrestricted global optimality over continuous sensor configurations.

## 3. Materials and Methods

This section develops the deployment framework from target-trajectory modeling to sensor selection. The decision is to select sensor locations and orientations from finite candidate sets, subject to a sensor budget and at most one selected orientation at each location. The objective is to maximize the expected number of detected target passages over the observation window under the specified trajectory-intensity model.

The formulation connects three components. First, the trajectory representation describes where targets travel, while the occurrence intensity weights passages according to their expected frequency. Second, the directional sensing model determines the detection opportunities available along each passage and combines them into a passage-level detection probability. Third, the network objective combines the responses of the selected sensors while accounting for overlapping detection of the same passage.

These components define the reference deployment problem. To reduce its computational cost, we introduce a dwell-time-aware binary sensing approximation and calibrate its sensing radius. We then describe directional greedy selection and the proposed omni-greedy procedure, which selects a location using an omnidirectional proxy before choosing its orientation using the directional objective. The following subsections introduce each component in this order; the theoretical analysis subsequently examines the errors introduced by the sensing approximation and the reduced search.

The following subsections introduce each component in this order. Section 4 examines the errors introduced by the sensing approximation and the reduced search. Table 2 summarizes the principal notation used throughout the deployment formulation and algorithms.

### 3.1. Deployment Problem and Framework Overview

Let $Q\subset R^{2}$ denote the bounded monitoring region, and let Ψ⊆Q denote the region in which sensors may be installed. These sets have different roles: Q specifies the target passages to be monitored, whereas Ψ specifies admissible sensor locations. They may coincide when no additional siting restrictions are imposed.

A directional sensor configuration is a pair c=(a,β), where a∈Ψ is its location, and β∈[0,2π) is its boresight orientation. Let

(1) $$L=\left\{a^{\left(1\right)},\ldots ,a^{\left(L\right)}\right\},\;B=\left\{\beta^{\left(1\right)},\ldots ,\beta^{\left(K\right)}\right\},\;V=L\times B$$

denote the candidate locations, orientations, and configurations, respectively. Here, L⊆Ψ and B⊂[0,2π), with L=|L| and K=|B|. Each element of V is one selectable location–orientation pair.

A deployment is a subset C⊆V. For a prescribed budget 1≤M≤L, the feasible deployment family is

(2) $$I_{M}=\left\{C\subseteq V:|C|\leq M,\left|C\cap (\{a\}\times B)\right|\leq 1\;\mathrm{for}\;\mathrm{every}\;a\in L\right\}.$$

Thus, each physical location can receive at most one sensor with one selected orientation. For a partial deployment *C*, define

L(C)={a:(a,β)∈C}

as the set of occupied locations.

**Figure 1:**
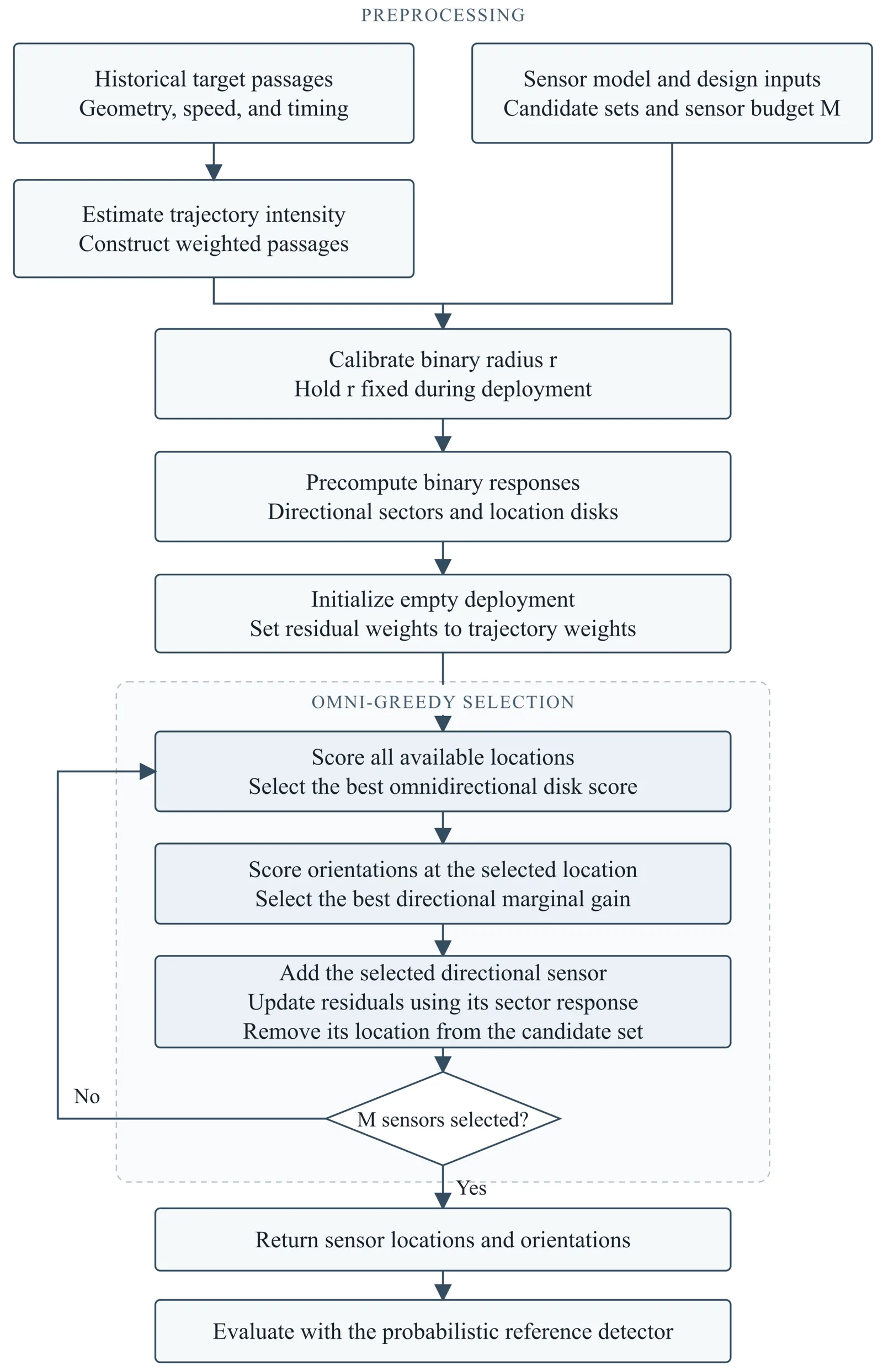
Overview of the proposed deployment framework. After trajectory-intensity estimation and binary-radius calibration, omni-greedy alternates between location selection and orientation refinement. Residual weights are updated using the selected directional sensor until the sensor budget is reached. The final deployment is evaluated using the probabilistic reference detector.

Figure 1 summarizes the preprocessing steps, iterative sensor-selection procedure, and final performance evaluation.

### 3.2. Trajectory Representation and Occurrence Model

To evaluate a candidate deployment, we must specify both the geometry of possible target passages and their relative frequency. The trajectory coordinates describe the directed path through the monitoring region; the associated motion information determines how long the target is exposed to a sensor. The occurrence intensity provides the statistical weight assigned to each passage when computing deployment performance. Thus, trajectory geometry determines sensing exposure, whereas trajectory intensity determines how strongly that exposure contributes to the objective.

#### 3.2.1. Trajectory Geometry and Motion

We consider linear or locally linear target passages through Q. The geometric component of a directed trajectory is represented by

x=(ξ,p)∈Ω=[0,2π)×R,

where ξ is the target heading, and *p* is the signed perpendicular offset from a fixed reference origin. This construction extends the transformed-line representation used in [8] by retaining the direction of travel.

Define the unit tangent and normal vectors

(3) $$v\left(\xi \right)=\left[\begin{array}{c} \cos\;\xi \\ \sin\;\xi \end{array}\right],\;n\left(\xi \right)=\left[\begin{array}{c} -\sin\;\xi \\ \cos\;\xi \end{array}\right].$$

The associated geometric line is

(4) $$q_{x}=\left\{s\in R^{2}:n{\left(\xi \right)}^{T}s=p\right\},$$

with traversal direction v(ξ). Let Θ⊆Ω denote the trajectory domain relevant to passages through Q.

**Figure 2:**
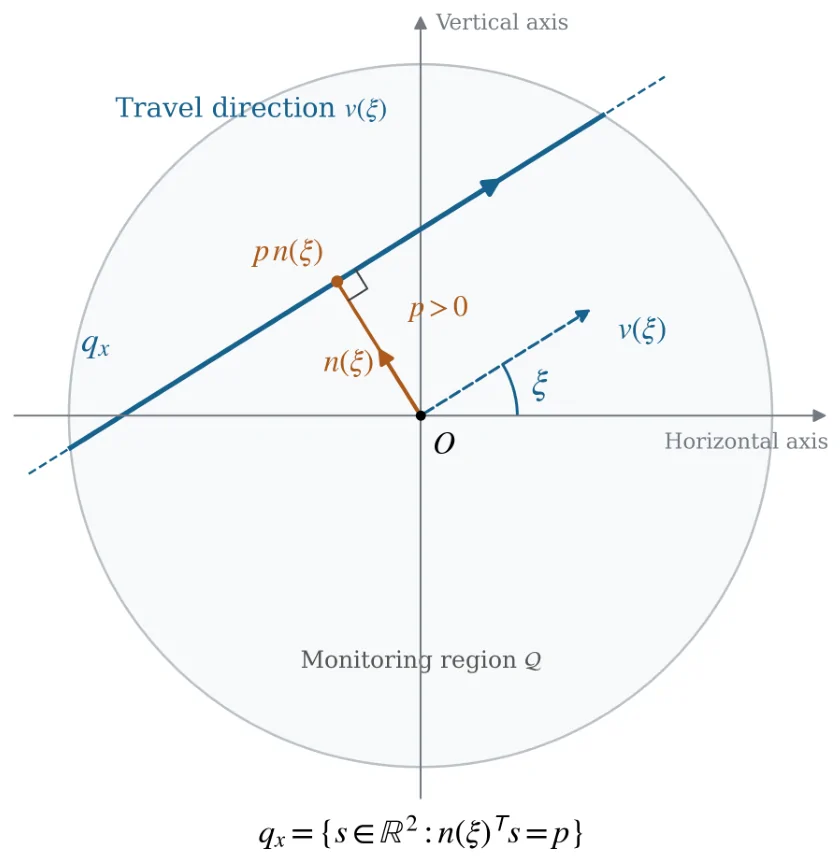
Directed-line representation of a target passage. Heading ξ specifies the travel direction v(ξ), and signed offset *p* locates the line $q_{x}=\left\{s:n{\left(\xi \right)}^{T}s=p\right\}$. The illustrated case has p>0, with perpendicular projection pn(ξ) from the origin.

Figure 2 illustrates the directed-line representation. The target heading ξ is measured counterclockwise from the positive horizontal axis and determines the direction of travel v(ξ). The normal vector n(ξ) is perpendicular to this direction, and the signed offset *p* locates the line relative to the reference origin. The point pn(ξ) is the perpendicular projection of the origin onto the line. Thus, ξ specifies the travel direction, while *p* specifies the line’s position.

A heading and offset specify a directed line, but not its temporal parameterization. Detection evaluation additionally requires a finite passage interval $I_{x}=\left[\tau_{x}^{\mathrm{in}},\tau_{x}^{\mathrm{out}}\right)$, an initial position, and a speed. For a constant-speed passage,

(5) $$s_{x}\left(\tau \right)=s_{x}\left(\tau_{x}^{\mathrm{in}}\right)+v_{x}\left(\tau -\tau_{x}^{\mathrm{in}}\right)v\left(\xi \right),\;\tau \in I_{x},$$

where $v_{x}>0$ is the prescribed or estimated target speed. The passage endpoints specify the finite segment evaluated inside the monitoring region.

Speed enters detection evaluation through the temporal parameterization of the passage. Let $l_{i}\left(x;r\right)$ denote the total length of the finite passage inside the radius-limited directional footprint $F_{i}\left(r\right)$. Under the constant-speed assumption,

(6) $$T_{i}\left(x;r\right)=\frac{l_{i}\left(x;r\right)}{v_{x}},\;\Gamma_{i,r}^{B}\left(x\right)=1\left\{l_{i}\left(x;r\right)\geq v_{x}\tau_{\min}\right\}.$$

Thus, for a fixed geometric passage, a higher speed reduces the dwell time and increases the path length required to satisfy the binary detection criterion.

Speed also determines the target positions at the periodic sampling times in (13). It therefore affects both the number and the locations of the probabilistic sensing opportunities. The exact detection probability depends jointly on speed, passage timing, sampling phase, and the distance-dependent sensor response; it is not determined by geometric intersection alone.

Throughout the formulation, $\Gamma_{i}\left(x\right)$ and $T_{i}\left(x;r\right)$ are evaluated conditional on the motion attributes assigned to trajectory *x*, including its speed and passage timing. These attributes are supplied by the trajectory model and are not determined by (ξ,p) alone. Accordingly, the analysis applies to the specified assignment of motion attributes. If multiple speeds or arrival times are modeled for the same directed line, those attributes must be included as trajectory marks and integrated over their joint occurrence distribution.

The pairs x=(ξ,p) and $x^{\mathrm{rev}}=\left(\left(\xi +\pi \right)\;\mathrm{mod}\;2\pi ,-p\right)$ represent opposite traversal directions along the same geometric line. For reciprocal traversals of the same finite segment at equal speed, the dwell time within any fixed sensing footprint is unchanged. Consequently, the binary detection response is identical for the two traversals.

For the static position-dependent probabilistic detector, the trajectory-level detection response is also unchanged when the traversals have the same multiset of sensing positions: reversal changes only the order of the factors in (13). A fixed periodic schedule does not automatically produce matched sensing positions, so any difference must be attributed to the specified sampling times or other differing motion attributes, rather than to heading alone.

Retaining directed headings permits direction-conditioned occurrence statistics and motion attributes, but does not by itself establish a detection advantage over an undirected representation. When reciprocal passages have identical sensor responses, their intensities can be aggregated without changing the deterministic deployment objective.

Specifically, let H(x;C)=1−Π(x;C) denote the network detection response. For a reciprocal pair with $H\left(x;C\right)=H\left(x^{\mathrm{rev}};C\right)$, its combined contribution can be written as

$$\bar{\lambda }\left(x\right)H\left(x;C\right)+\bar{\lambda }\left(x^{\mathrm{rev}}\right)H\left(x^{\mathrm{rev}};C\right)=\left[\bar{\lambda }\left(x\right)+\bar{\lambda }\left(x^{\mathrm{rev}}\right)\right]H\left(x;C\right).$$

Thus, aggregation over reciprocal pairs preserves the objective when response equivalence holds for every candidate configuration, and the intensity mass is preserved.

#### 3.2.2. Trajectory Occurrence

Let $\bar{\lambda }\left(x\right)\geq 0$ denote the expected occurrence density of trajectories over a specified observation window. Its scale is chosen so that

(7) $$\Lambda_{0}=\int_{\Theta }\bar{\lambda }\left(x\right)\;dx<\infty$$

is the expected number of target passages in that window. Thus, trajectory intensity contains both the relative distribution of passages and their expected total count.

The deployment formulation below accepts a fixed nonnegative planning intensity $\bar{\lambda }$. An LGCP provides an optional model for uncertainty in trajectory occurrence and connects this formulation to the posterior-averaged void probability:

(8) Y∼GP(μ,Σ),λ(x)=exp(Y(x)).

Conditioned on λ, trajectory occurrences form an inhomogeneous Poisson process on Θ. If a posterior distribution is fitted using historical data H, then $\bar{\lambda }\left(x\right)=E\left[\lambda \left(x\right)|H\right]$.

The deployment algorithms require a nonnegative intensity $\bar{\lambda }$, rather than a particular estimation method. In the numerical studies, this intensity is constructed using kernel density estimation, not a fitted LGCP posterior. A normalized density estimate must be scaled by the estimated number of passages in the observation window when an absolute missed-trajectory intensity or void probability is reported. The general LGCP model and the deterministic intensity used in the experiments are therefore distinguished explicitly.

### 3.3. Directional and Trajectory-Level Detection

Given a target passage and a sensor configuration, the next step is to determine whether the sensor can detect the target during that passage. The sensor location and orientation determine which portions of the path lie within its field of view, while the distance-dependent response and sampling convention determine the available detection opportunities. Under the stated independence assumptions, these opportunities are combined into the probability of detecting the passage at least once.

#### 3.3.1. Instantaneous Sensing

For configuration $c_{i}=\left(a_{i},\beta_{i}\right)$, define the boresight vector

(9) $$u\left(\beta_{i}\right)=\left[\begin{array}{c} \cos\;\beta_{i}\\ \sin\;\beta_{i} \end{array}\right].$$

For half-FOV angle ϕ∈(0,π/2], the directional observation region is

(10) $$F_{i}=\left\{s\in Q\setminus \left\{a_{i}\right\}:\frac{{\left(s-a_{i}\right)}^{T}u\left(\beta_{i}\right)}{∥s-a_{i}∥}\geq \cos\;\phi \right\}.$$

Let $\rho_{\max}\in \left(0,1\right]$ be the maximum instantaneous detection probability, and let y(d;l)∈[0,1] be a nonincreasing radial response satisfying y(0;l)=1, with distance-scale parameter l>0. The radial detection probability is

$$\eta_{i}\left(s\right)=\rho_{\max}y(∥s-a_{i}∥;l).$$

Using an effective sensing horizon $R_{\mathrm{sens}}>0$, define

(11) $$\gamma_{i}\left(s\right)=\eta_{i}\left(s\right)1\left\{s\in F_{i}\right\}1\{∥s-a_{i}∥\leq R_{\mathrm{sens}}\}.$$

The three factors represent distance-dependent detection, directional visibility, and the finite sensing horizon, respectively. The exact radial kernel and its parameters must be specified for each numerical sensor model. At $s=a_{i}$, where the angular expression is undefined, a fixed convention may be adopted; this isolated point does not affect integrals under a continuous trajectory distribution.

#### 3.3.2. Sensing Opportunities During a Passage

Suppose sensor *i* samples periodically at frequency f>0 with phase $\vartheta_{i}\in \left[0,1/f\right)$. Its sampling schedule is

$$S_{i}=\left\{\vartheta_{i}+k/f:k\in Z\right\}.$$

For trajectory *x*, let ${\left\{\tau_{i,k}\left(x\right)\right\}}_{k=1}^{N_{i}\left(x\right)}$ be the ordered elements of $S_{i}$ that lie in $I_{x}$ and satisfy both FOV membership and the sensing-horizon constraint. Consequently,

(12) $$N_{i}\left(x\right)=\sum_{\tau \in S_{i}\cap I_{x}}1\left\{s_{x}\left(\tau \right)\in F_{i}\right\}1\{∥s_{x}\left(\tau \right)-a_{i}∥\leq R_{\mathrm{sens}}\}.$$

This definition makes the sampling phase and finite passage explicit. For sample membership, observation intervals include entry and exclude exit; isolated zero-duration contacts are excluded. The same convention is applied to the inner and outer sensing regions used in the analysis.

We model each sensing opportunity as a Bernoulli trial whose success probability is determined by the target position and the specified sensor response. Conditional on the complete passage, including its motion attributes and sampling times, we assume that the remaining trial-to-trial detection errors are independent. This is a simplifying observation-model assumption: it represents detection fluctuations without temporal memory after the modeled conditions are fixed. Under this assumption, the probability of at least one detection is

(13) $$\Gamma_{i}\left(x\right)=1-\prod_{k=1}^{N_{i}\left(x\right)}\left[1-\gamma_{i}\left(s_{x}\left(\tau_{i,k}\left(x\right)\right)\right)\right].$$

An empty product equals one, so $\Gamma_{i}\left(x\right)=0$ when no sensing opportunity occurs.

Conditioning on the passage does not itself establish independence. Persistent occlusion, temporally correlated noise, or detector processing that combines successive observations can violate this assumption. The product model therefore serves as the probabilistic reference detector for the present analysis; the trajectory datasets do not contain detection outcomes with which to validate its independence assumption.

For comparison with dwell-time approximations, define the observable duration within the effective sensing region as

(14) $$T_{i}\left(x;R_{\mathrm{sens}}\right)=\int_{I_{x}}1\left\{s_{x}\left(\tau \right)\in F_{i}\right\}1\{∥s_{x}\left(\tau \right)-a_{i}∥\leq R_{\mathrm{sens}}\}\;d\tau .$$

A dwell-based approximation is

(15) $$N_{i}\left(x\right)\approx \left\lfloor fT_{i}\left(x;R_{\mathrm{sens}}\right)\right\rfloor .$$

Equation (15) is not an exact sampling rule: the actual count also depends on the relative timing of the passage and the sampling schedule. Detection evaluation and any sample-count bounds must use a consistent timing convention.

#### 3.3.3. Network Detection

At the network level, we additionally assume that sensor-level detection outcomes are independent conditional on the same complete passage and the specified sensing conditions. This assumption excludes residual dependence caused by shared disturbances or common occlusion. It permits the network missed-detection probability to be written as

(16) $$\Pi \left(x;C\right)=\prod_{c_{i}\in C}\left(1-\Gamma_{i}\left(x\right)\right),$$

and its network detection probability is

$$\hat{\Gamma }\left(x;C\right)=1-\Pi \left(x;C\right).$$

Physical separation alone does not justify this factorization. If a shared random condition affects several sensors, the joint missed-detection probability must account for that condition. Even when independence holds conditional on a shared latent variable, averaging over that variable generally does not equal multiplying the separately averaged missed-detection probabilities. Consequently, the present product-based expressions and their associated error bounds apply to the stated independent observation model. The multiplicative missed-detection probability accounts for overlapping coverage without counting a trajectory more than once. A sensor provides less additional benefit for trajectories already likely to be detected by the existing deployment.

### 3.4. Deployment Objective

The network detection probability defined above connects individual sensor responses to deployment performance. Integrating this probability against $\bar{\lambda }\left(x\right)$ gives the expected number of detected passages over the specified observation window. No additional multiplication by the window duration is needed because $\bar{\lambda }\left(x\right)$ already represents trajectory occurrence over that window. This objective weights passages by their expected frequency while counting each detected passage only once.

For a fixed intensity $\bar{\lambda }$, the expected missed-trajectory count is

(17) $$\tilde{\Lambda }\left(C\right)=\int_{\Theta }\bar{\lambda }\left(x\right)\Pi \left(x;C\right)\;dx.$$

The corresponding expected detected-trajectory count is

(18) $$\begin{array}{cc} F(C) & =\tilde{\Lambda }\left(⌀\right)-\tilde{\Lambda }\left(C\right)\\ & =\int_{\Theta }\bar{\lambda }\left(x\right)\left[1-\prod_{c_{i}\in C}\left(1-\Gamma_{i}\left(x\right)\right)\right]dx. \end{array}$$

The finite-candidate deployment problem is

(19) $${\hat{C}}^{★}\in \underset{C\in I_{M}}{\mathrm{argmax}}F\left(C\right).$$

Thus, the optimization explicitly enforces both the sensor budget and location exclusivity.

For a feasible additional configuration *c*, the continuous marginal gain is

(20) $$\begin{array}{cc} \Delta (c|C) & =F(C\cup \{c\})-F(C)\\ & =\int_{\Theta }\bar{\lambda }\left(x\right)\Gamma_{c}\left(x\right)\Pi \left(x;C\right)\;dx. \end{array}$$

The residual weight $\bar{\lambda }\left(x\right)\Pi \left(x;C\right)$ emphasizes frequent trajectories that remain poorly detected by *C*. Under this sensing model, *F* is normalized, monotone, and submodular, as established in Proposition 1.

#### Relationship to Void Probability

Under a deterministic Poisson intensity and independent thinning across trajectory occurrences, the probability that no trajectory is missed is

$$\tilde{\nu }\left(C\right)=\exp\left[-\tilde{\Lambda }\left(C\right)\right].$$

Maximizing this probability is equivalent to maximizing F(C) because $\tilde{\Lambda }\left(⌀\right)$ is independent of the deployment.

For an LGCP, the corresponding posterior-averaged probability is instead

(21) $$\nu \left(C\right)=E_{\lambda |H}\left[\exp\left(-\int_{\Theta }\lambda \left(x\right)\Pi \left(x;C\right)\;dx\right)\right].$$

Provided the trajectory-dependent sensing response is fixed under this expectation, Jensen’s inequality gives

(22) $$\nu \left(C\right)\geq \exp\left[-\int_{\Theta }\bar{\lambda }\left(x\right)\Pi \left(x;C\right)\;dx\right]=\tilde{\nu }\left(C\right).$$

The general stochastic objective would be

(23) $$C^{★}\in \underset{C\in I_{M}}{\mathrm{argmax}}\nu \left(C\right),$$

whereas the algorithms below optimize (19). An optimizer of the lower-bound surrogate need not optimize the full posterior-averaged probability.

For $0<\Lambda_{0}<\infty$, the normalized detected intensity $F\left(C\right)/\Lambda_{0}$ can also be reported. It expresses the expected detected fraction and does not depend on a common multiplicative scaling of $\bar{\lambda }$. In contrast, void probability depends on the expected number of passages and therefore on the observation window.

### 3.5. Binary Sensing Approximation and Radius Calibration

The probabilistic model provides the reference detection response, but its evaluation requires combining sensing opportunities along target passages. The binary approximation replaces this response with a dwell-time criterion within a radius-limited directional footprint. The binary radius controls the spatial extent of that footprint, while the minimum dwell time specifies the required exposure. Radius calibration relates this simplified response to the probabilistic reference model; the binary response is an approximation rather than a claim that physical detection is certain.

#### 3.5.1. Dwell-Time-Aware Binary Detector

For radius $0<r\leq R_{\mathrm{sens}}$, define

(24) $$F_{i}\left(r\right)=F_{i}\cap \{s:∥s-a_{i}∥\leq r\},$$

and the corresponding passage duration

(25) $$T_{i}\left(x;r\right)=\int_{I_{x}}1\left\{s_{x}\left(\tau \right)\in F_{i}\left(r\right)\right\}\;d\tau .$$

For a prescribed minimum dwell time $\tau_{\min}>0$, the binary detector is

(26) $$\Gamma_{i,r}^{B}\left(x\right)=1\left\{T_{i}\left(x;r\right)\geq \tau_{\min}\right\}.$$

Unlike a pure intersection test, this criterion rejects passages that only graze the sensing sector without providing sufficient observation time. Nevertheless, a binary-positive passage is assigned probability one by the surrogate; it is not guaranteed to be detected with certainty by the probabilistic reference model.

The dwell-based count

(27) $$N_{\min}=\left\lfloor f\tau_{\min}\right\rfloor$$

is used as a lower bound on sensing opportunities only under the appropriate sampling and interval assumptions. For a single contiguous half-open observable interval and a periodic schedule, the count is at least the floor of its duration multiplied by *f*. This conclusion cannot be transferred without modification to a total duration formed from multiple disjoint intervals. When this bound is used, we require $N_{\min}\geq 1$.

#### 3.5.2. Binary Deployment Objective

The single-sensor binary detector specifies whether an individual passage satisfies a sensor’s dwell-time criterion. Deployment planning, however, requires evaluating a collection of sensors whose coverage may overlap. Simply summing individual sensor benefits would count passages covered by multiple sensors more than once.

We therefore define a binary network objective that counts each passage once whenever at least one selected sensor satisfies the dwell-time criterion. This objective supplies the marginal gains used by binary directional greedy and by the orientation-selection step of omni-greedy. Its missed-detection indicator also supplies the directional residual used to rank subsequent candidate locations. Replacing $\Gamma_{i}$ by $\Gamma_{i,r}^{B}$ in the network model gives

(28) $$\begin{array}{c} \Pi_{r}^{B}\left(x;C\right)=\prod_{c_{i}\in C}\left[1-\Gamma_{i,r}^{B}\left(x\right)\right], \end{array}$$

(29) $$\begin{array}{c} \Lambda_{r}^{B}\left(C\right)=\int_{\Theta }\bar{\lambda }\left(x\right)\Pi_{r}^{B}\left(x;C\right)\;dx, \end{array}$$

(30) $$\begin{array}{c} F_{r}^{B}\left(C\right)=\Lambda_{0}-\Lambda_{r}^{B}\left(C\right). \end{array}$$

The associated marginal gain is

(31) $$\begin{array}{cc} \Delta_{r}^{B}\left(c|C\right) & =F_{r}^{B}\left(C\cup \left\{c\right\}\right)-F_{r}^{B}\left(C\right)\\ & =\int_{\Theta }\bar{\lambda }\left(x\right)\Gamma_{c,r}^{B}\left(x\right)\Pi_{r}^{B}\left(x;C\right)\;dx. \end{array}$$

Thus, the binary marginal gain is the intensity of previously uncovered trajectories satisfying the candidate sensor’s dwell-time criterion.

#### 3.5.3. Radius Calibration

The radius *r* controls the discrepancy between binary and probabilistic detection. A small radius can exclude passages with meaningful detection probability, while a large radius can classify weakly observed passages as perfectly detected. Radius selection therefore compares the binary response with the reference model rather than assigning a range from an arbitrary instantaneous-probability threshold.

Let $R\subset (0,R_{\mathrm{sens}}]$ be the admissible radius set. Section 4.5 defines the deployment-aware error envelope $E_{M}\left(r\right)$ and the calibration rule

$$r^{★}\in \underset{r\in R}{\mathrm{argmin}}E_{M}\left(r\right).$$

The envelope aggregates intensity-weighted single-sensor error bounds while accounting for the sensor budget and location exclusivity. Its construction and validity conditions are given in Section 4.

For a finite radius grid, calibration evaluates the envelope at each admissible radius and selects a minimizer using a fixed tie-breaking rule. The selected radius is held fixed during the deployment run described below. Calibration is a separate preprocessing cost, and a conservative error envelope may provide a loose certificate even when empirical sensing errors are small.

### 3.6. Directional Greedy and Omni-Greedy Deployment

With the objective and sensing responses defined, the remaining task is to select feasible sensor configurations. A marginal gain measures the additional expected detection contributed by a candidate sensor given the current partial deployment. Directional greedy evaluates this gain for every available location–orientation pair. Omni-greedy reduces the search by first ranking available locations using an omnidirectional proxy and then evaluating orientations at the selected location. After each selection, the directional residual is updated so that subsequent decisions account for passages already covered.

#### 3.6.1. Directional Greedy Benchmark

Starting from $C_{0}=⌀$, binary directional greedy selects

(32) $$c_{t}^{\mathrm{DG}}\in \underset{\begin{array}{c} a\in L\setminus L(C_{t-1})\\ \beta \in B \end{array}}{\mathrm{argmax}}\Delta_{r}^{B}\left(\left(a,\beta \right)|C_{t-1}\right),$$

and sets $C_{t}=C_{t-1}\cup \left\{c_{t}^{\mathrm{DG}}\right\}$. Continuous directional greedy uses the same feasible candidates but replaces $\Delta_{r}^{B}$ with the continuous marginal gain Δ in (20).

These benchmarks separate two comparisons: continuous versus binary directional greedy assesses the sensing approximation, while binary directional greedy versus binary omni-greedy assesses the reduced location–orientation search. All returned deployments can additionally be evaluated using the same probabilistic reference detector.

#### 3.6.2. Omnidirectional Location Proxy

For a candidate location *a*, define

(33) $$\begin{array}{cc} D(a,r) & =\{s\in Q:∥s-a∥\leq r\}, \end{array}$$

(34) $$\begin{array}{cc} T_{a}^{O}\left(x;r\right) & =\int_{I_{x}}1\left\{s_{x}\left(\tau \right)\in D\left(a,r\right)\right\}\;d\tau , \end{array}$$

(35) $$\begin{array}{cc} \Gamma_{a,r}^{O}\left(x\right) & =1\{T_{a}^{O}\left(x;r\right)\geq \tau_{\min}\}. \end{array}$$

The omnidirectional location score is

(36) $$\Delta_{r}^{O}\left(a|C\right)=\int_{\Theta }\bar{\lambda }\left(x\right)\Gamma_{a,r}^{O}\left(x\right)\Pi_{r}^{B}\left(x;C\right)\;dx.$$

It measures residual trajectory exposure near *a* without selecting a sensor orientation. Because a disk can include trajectories that cannot all be covered by a single directional sector, this score need not preserve the ordering of the best directional marginal gains.

Location is selected first because the omnidirectional footprint provides an orientation-independent measure of residual trajectory exposure around each candidate site. Once a site is selected, orientation refinement determines which directional sector captures the greatest remaining benefit there. This ordering is a computational design choice, not a claim that location must always precede orientation.

An orientation-first procedure is also possible, but it would require a score that aggregates or optimizes over candidate locations for each orientation. Exact maximization over locations would require evaluating all available location–orientation pairs unless additional structure or acceleration were available; a cheaper aggregation would introduce a different approximation. The present work analyzes the location-first construction and does not claim that it universally outperforms an orientation-first alternative.

#### 3.6.3. Location Selection and Orientation Refinement

At iteration *t*, omni-greedy first selects

(37) $$a_{t}\in \underset{a\in L\setminus L(C_{t-1})}{\mathrm{argmax}}\Delta_{r}^{O}\left(a|C_{t-1}\right),$$

then selects the orientation at that location:

(38) $$\beta_{t}\in \underset{\beta \in B}{\mathrm{argmax}}\Delta_{r}^{B}\left(\left(a_{t},\beta \right)|C_{t-1}\right).$$

The deployment update is

(39) $$C_{t}=C_{t-1}\cup \left\{\left(a_{t},\beta_{t}\right)\right\}.$$

The residual missed-detection indicator is updated using the selected *directional* sensor:

(40) $$\Pi_{r}^{B}\left(x;C_{t}\right)=\Pi_{r}^{B}\left(x;C_{t-1}\right)\left[1-\Gamma_{(a_{t},\beta_{t}),r}^{B}\left(x\right)\right].$$

The omnidirectional disk is used only for location ranking and is never used to remove trajectories from the residual.

#### 3.6.4. Solution of the Two Maximization Problems

Both selection problems are solved by exhaustive evaluation over finite candidate sets; no continuous or iterative optimization solver is required. At iteration *t*, the algorithm evaluates $\Delta_{r}^{O}\left(a|C_{t-1}\right)$ for each of the L−t+1 unoccupied locations and selects a maximizer. With this location fixed, it evaluates $\Delta_{r}^{B}\left(\left(a_{t},\beta \right)|C_{t-1}\right)$ for all *K* candidate orientations and selects a maximizer. A fixed candidate ordering resolves ties reproducibly.

These maximizations are exact with respect to the computed scores on the specified candidate sets. Nevertheless, the overall procedure is approximate: the location is chosen using an omnidirectional proxy, the sensing response is approximated by a binary model, and the trajectory integrals are evaluated numerically. In particular, optimizing orientation after location selection does not generally recover the configuration obtained by jointly searching all location–orientation pairs.

#### 3.6.5. Finite Numerical Implementation

Let ${\left\{x_{j},w_{j}\right\}}_{j=1}^{J}$ be trajectory quadrature nodes and nonnegative intensity weights such that

$$\int_{\Theta }\bar{\lambda }\left(x\right)h\left(x\right)\;dx\approx \sum_{j=1}^{J}w_{j}h\left(x_{j}\right).$$

Each node is supplied with the motion attributes required by the sensing model. The weights include the intensity and integration weights; they are not necessarily normalized to sum to one.

The index *j* identifies a trajectory quadrature node, and *k* identifies a candidate orientation. In Algorithm 1, A contains the currently unoccupied candidate locations, while $g_{a}$ and $h_{k}$ store the location and orientation scores, respectively.

For the calibrated radius, precompute

$$B_{j,a,k}=\Gamma_{(a,\beta^{\left(k\right)}),r}^{B}\left(x_{j}\right),\;O_{j,a}=\Gamma_{a,r}^{O}\left(x_{j}\right).$$

If $z_{j}$ is the current binary missed-detection indicator, then location and orientation scores are evaluated as

$$\sum_{j=1}^{J}w_{j}O_{j,a}z_{j},\;\sum_{j=1}^{J}w_{j}B_{j,a,k}z_{j},$$

respectively. Algorithm 1 gives the resulting procedure.
**Algorithm 1** Binary Omni-Greedy Sensor Placement and Orientation**Require:** Candidate locations L, orientations B, budget M≤L, weights ${\left\{w_{j}\right\}}_{j=1}^{J}$, and precomputed responses $B_{j,a,k}$ and $O_{j,a}$ at calibrated radius *r*  1:C←⌀, A←L  2:$z_{j}\leftarrow 1$ for j=1,…,J  3:**for** t=1,…,M **do**  4:       **for all** a∈A **do**  5:             $g_{a}\leftarrow \sum_{j=1}^{J}w_{j}O_{j,a}z_{j}$  6:       **end for**  7:       $a_{t}\leftarrow {\mathrm{argmax}}_{a\in A}g_{a}$  8:       **for** k=1,…,K **do**  9:             $h_{k}\leftarrow \sum_{j=1}^{J}w_{j}B_{j,a_{t},k}z_{j}$ 10:       **end for** 11:       $k_{t}\leftarrow {\mathrm{argmax}}_{k=1,\ldots ,K}h_{k}$ 12:       $C\leftarrow C\cup \{\left(a_{t},\beta^{\left(k_{t}\right)}\right)\}$ 13:       $z_{j}\leftarrow z_{j}\left(1-B_{j,a_{t},k_{t}}\right)$ for j=1,…,J 14:       $A\leftarrow A\setminus \{a_{t}\}$ 15:**end for** 16:**return** *C*

#### 3.6.6. Computational Tradeoff

Without lazy evaluation or other pruning, directional greedy evaluates

(41) $$K\sum_{t=1}^{M}\left(L-t+1\right)=K\left[ML-\frac{M(M-1)}{2}\right]=O\left(MLK\right)$$

candidate scores. Omni-greedy instead evaluates

(42) $$\sum_{t=1}^{M}\left(L-t+1+K\right)=M\left(L+K\right)-\frac{M(M-1)}{2}=O\left(M\left(L+K\right)\right)$$

scores. A direct weighted-sum implementation introduces an additional factor of *J* in the corresponding selection-stage arithmetic costs.

For fixed trajectories and candidate configurations, both binary and continuous sensing responses can be precomputed and cached. Consequently, the reduction in candidate evaluations arises from location–orientation decoupling, not from requiring the continuous benchmark to recompute its detector responses at every iteration. Precomputation, radius calibration, selection, and optional certification should be reported separately when comparing total runtime. Storing all directional and omnidirectional response arrays requires O(JLK+JL) entries; implementations can trade storage for on-demand evaluation.

The computational saving comes with a possible loss in directional marginal gain because the location proxy does not jointly optimize orientation. Section 4 characterizes this discrepancy, gives conditions for agreement with directional greedy, and relates the sensing and search errors to deployment performance. The applicable greedy guarantee is determined by the feasibility constraints in (2); agreement with a greedy benchmark alone does not establish global optimality.

## 4. Theoretical Analysis

The framework in Section 3 introduces two computational approximations: replacing probabilistic trajectory-level detection with a dwell-time-aware binary model parameterized by radius *r*, and replacing joint location–orientation enumeration with omni-greedy location selection followed by directional orientation refinement. This section examines how these approximations affect deployment decisions and detection performance. The supporting analysis is presented separately to keep the modeling and implementation procedure accessible without requiring readers to follow the derivations.

The two approximations serve distinct purposes and need not be used together. Binary directional greedy uses the sensing approximation while retaining joint location–orientation search. Omni-greedy additionally reduces the number of candidate scores evaluated during selection. Binary sensing can perturb candidate marginal gains, whereas the omnidirectional location proxy can change the ranking of locations relative to their best directional gains. Separating these effects allows their individual contributions to deployment error to be assessed.

The analysis therefore proceeds from sensor-level approximation error to network-level error, radius calibration, greedy-decision preservation, location–orientation decoupling, and finally an end-to-end deployment guarantee. This order establishes bounds for the sensing approximation before examining the additional search error and combining their effects. In practical terms, the results support radius selection, identify conditions under which approximate scores preserve greedy choices, and assess the potential loss associated with reduced search. Failure of a sufficient condition indicates a lack of certification, rather than necessarily poor deployment performance.

The Jensen lower bound in (22) concerns a separate approximation: using the posterior-mean intensity in place of averaging the void probability over the random LGCP intensity. The tightness of that bound is outside the present analysis. Accordingly, the guarantees below concern the deterministic probabilistic reference model with fixed planning intensity $\bar{\lambda }$. Maximizing its detected-passage objective F(C) is equivalent to minimizing $\tilde{\Lambda }\left(C\right)$ and maximizing $\tilde{\nu }\left(C\right)$, but quantitative performance bounds must be translated through the relationships between these objectives. The guarantees do not directly apply to the full posterior-averaged LGCP void probability.

### 4.1. Preliminaries and Analysis Assumptions

Whenever a target lies inside the directional FOV, define its instantaneous detection probability at distance *d* as

(43) $$\begin{array}{cc} p(d) & =\rho_{\max}y\left(d;l\right). \end{array}$$

**Assumption 1** 
(Radial sensing kernel)**.** *The radial kernel y(d;l) is continuous and monotonically decreasing for d≥0, satisfies*

(44) $$\begin{array}{cc} y(0;l) & =1, \end{array}$$

*and obeys*

(45) $$\begin{array}{cc} \lim_{d\to \infty }y\left(d;l\right) & =0. \end{array}$$

For binary radius *r*, define

(46) $$\begin{array}{c} p_{r}=p\left(r\right)=\rho_{\max}y\left(r;l\right). \end{array}$$

The floor expression in (15) is a dwell-based approximation, not an exact count under a fixed periodic sampling schedule. The following analysis instead uses the actual scheduled sensing opportunities.

**Assumption 2** 
(Sampling schedule and inner observation interval)**.** *Sensor i samples at times $S_{i}=\left\{\vartheta_{i}+k/f:k\in Z\right\}$. For every admissible passage, sensor configuration, and binary radius, the times spent inside $F_{i}\left(r\right)$ form either an empty set or a single finite interval. Entry is included, and exit is excluded when counting samples; isolated zero-duration contacts are excluded. This convention is used consistently in the reference detector and the bounds.*

For a nonempty inner observation interval [a,b) of duration T=b−a, the exact scheduled sample count is

(47) $$N_{i}\left(\left[a,b\right)\right)=\left\lceil f(b-\vartheta_{i})\right\rceil -\left\lceil f(a-\vartheta_{i})\right\rceil .$$

Consequently,

(48) $$\left\lfloor fT\right\rfloor \leq N_{i}\left(\left[a,b\right)\right)\leq \left\lceil fT\right\rceil .$$

Although the exact count depends on the sampling phase, these bounds hold for every phase.

Define

(49) $$N_{\min}:=\left\lfloor f\tau_{\min}\right\rfloor .$$

**Assumption 3** 
(Minimum binary dwell time)**.** *The binary dwell threshold satisfies $N_{\min}\geq 1$.*

A binary-positive passage has $T_{i}\left(x;r\right)\geq \tau_{\min}$ and therefore at least $N_{\min}$ inner sensing opportunities. A binary-negative passage has $T_{i}\left(x;r\right)<\tau_{\min}$ and at most

(50) $$N_{<}:=\left\lceil f\tau_{\min}\right\rceil$$

inner sensing opportunities. The subscript refers to dwell time below the threshold, not to a sample count strictly below $f\tau_{\min}$.

Let

(51) $$\begin{array}{cc} R & =[r_{\min},r_{\max}] \end{array}$$

denote the admissible binary-radius interval. When an effective sensing horizon $R_{\mathrm{sens}}$ is imposed on the continuous reference model, we require

(52) $$\begin{array}{c} r_{\max}\leq R_{\mathrm{sens}}. \end{array}$$

Thus, binary-radius calibration is restricted to the same sensing domain used by the continuous reference model. Continuous sensing may also remain nonzero outside the binary radius. We therefore require a finite bound on the number of relevant outer-region sensing opportunities.

For each passage, define the outer-region sensing count using the actual sampling schedule:

(53) $$N_{i}^{\mathrm{out}}\left(x;r\right)=\sum_{\tau \in S_{i}\cap I_{x}}1\left\{s_{x}\left(\tau \right)\in F_{i}\right\}1\{r<∥s_{x}\left(\tau \right)-a_{i}∥\leq R_{\mathrm{sens}}\}.$$

Sample membership uses the same boundary convention as the reference detector.

**Assumption 4** 
(Uniform bound on outer sensing opportunities)**.** *For every admissible radius r∈R, there exists a finite nonnegative integer $N_{\max}\left(r\right)$ such that*

(54) $$N_{i}^{\mathrm{out}}\left(x;r\right)\leq N_{\max}\left(r\right)$$

*for every admissible sensor configuration and passage. The bound is nonincreasing in r, and $N_{\max}\left(R_{\mathrm{sens}}\right)=0$ whenever that radius is included in the admissible domain.*

A sufficient condition for a finite uniform count is a uniform upper bound on passage duration. Alternatively, a bounded sensing region combined with straight, constant-speed passages and a positive lower speed bound provides such a bound. A finite spatial sensing horizon alone does not establish a uniform count if speeds can approach zero or passage durations are unbounded.

If $N_{\max}\left(r\right)$ is computed by enumeration over a finite trajectory and candidate set, its validity is restricted to that evaluated set unless a separate uniform argument is supplied.

Before analyzing either approximation, we first establish the structural property that makes greedy deployment meaningful. In particular, both the continuous and binary objectives should exhibit diminishing returns: once a trajectory is already well covered, an additional sensor should provide a smaller incremental benefit. The following proposition formalizes this property and provides the basis for the greedy guarantees developed later.

**Proposition 1** 
(Monotonicity and submodularity)**.** *Under the conditional-independence sensing model, the continuous benefit F(C) and binary benefit $F_{r}^{B}\left(C\right)$ are normalized, monotone, and submodular over the finite directional candidate set V.*

**Proof.**  Using (16) and (18),

(55) $$\begin{array}{cc} F(C) & =\int_{\Theta }\bar{\lambda }\left(x\right)\left[1-\Pi (x;C)\right]dx. \end{array}$$

Since Π(x;⌀)=1, we have F(⌀)=0.

For candidate c∉C, the continuous marginal gain is

(56) $$\begin{array}{cc} \Delta (c|C) & =F(C\cup \{c\})-F(C)\\ & =\int_{\Theta }\bar{\lambda }\left(x\right)\Gamma_{c}\left(x\right)\Pi \left(x;C\right)dx\\ & \geq 0. \end{array}$$

Hence, *F* is monotone.

Now, let A⊆B and c∉B. Since every factor $1-\Gamma_{i}\left(x\right)$ lies in [0,1],

(57) $$\begin{array}{c} \Pi (x;A)\geq \Pi (x;B). \end{array}$$

Therefore,

(58) $$\begin{array}{c} \Delta (c|A)\geq \Delta (c|B), \end{array}$$

which establishes diminishing returns and hence submodularity.

Replacing $\Gamma_{c}\left(x\right)$ and Π(x;C) by $\Gamma_{c,r}^{B}\left(x\right)$ and $\Pi_{r}^{B}\left(x;C\right)$ gives the same result for $F_{r}^{B}\left(C\right)$. □

Proposition 1 establishes the optimization structure needed later for both directional greedy and the final matroid-constrained performance guarantee. We next quantify the first source of approximation: replacing continuous trajectory-level detection by the binary sensing model.

### 4.2. Binary-Positive Overestimation

A binary-positive trajectory is assigned detection probability one, whereas its continuous detection probability may be smaller.

**Lemma 1** 
(Binary-positive detection bound)**.** *Under Assumptions 1, 2 and 4, and the conditional-independence sensing model, if*

(59) $$\begin{array}{cc} \Gamma_{i,r}^{B}\left(x\right) & =1, \end{array}$$

*then*

(60) $$\begin{array}{c} \Gamma_{i}\left(x\right)\geq 1-{\left(1-p_{r}\right)}^{N_{\min}}. \end{array}$$

*Consequently,*

(61) $$\begin{array}{c} 0\leq \Gamma_{i,r}^{B}\left(x\right)-\Gamma_{i}\left(x\right)\leq e_{\mathrm{in}}\left(r\right), \end{array}$$

*where*

(62) $$\begin{array}{cc} e_{\mathrm{in}}\left(r\right) & ={\left(1-p_{r}\right)}^{N_{\min}}. \end{array}$$

**Proof.**  Since $\Gamma_{i,r}^{B}\left(x\right)=1$, $T_{i}\left(x;r\right)\geq \tau_{\min}$. By Assumption 2, the inner observation interval is a single half-open interval of duration $T_{i}\left(x;r\right)$, so by (48), its sample count satisfies

$$N_{i}^{\mathrm{in}}\left(x;r\right)\geq \left\lfloor fT_{i}\left(x;r\right)\right\rfloor \geq \left\lfloor f\tau_{\min}\right\rfloor =N_{\min}.$$

Each inner sample lies within distance *r* of $a_{i}$, so by Assumption 1, its detection probability is at least $p_{r}$. Retaining only these samples,

(63) $$\begin{array}{cc} \Gamma_{i}\left(x\right) & =1-\prod_{k}\left[1-p(d_{k})\right]\\ & \geq 1-{\left(1-p_{r}\right)}^{N_{\min}}. \end{array}$$

Since the binary model assigns value one, (61) follows. □

Repeated sensing therefore improves the binary-positive approximation exponentially with $N_{\min}$. However, $p_{r}$ decreases with *r*, so $e_{\mathrm{in}}\left(r\right)$ is nondecreasing in *r*. Enlarging the binary region therefore weakens the guarantee associated with declaring every binary-positive trajectory perfectly detected.

Lemma 1 controls only one side of the binary approximation: trajectories that the binary model declares detected. A complete error characterization also requires the converse case, because a binary-negative trajectory may still have nonzero continuous detection probability.

### 4.3. Binary-Negative Underestimation

A binary-negative trajectory can still be detected continuously for two reasons: it may spend less than $\tau_{\min}$ inside the radius-*r* FOV, or it may generate nonzero sensing opportunities outside radius *r*.

**Lemma 2** 
(Binary-negative detection bound)**.** *Under Assumptions 1–4, if*

(64) $$\begin{array}{cc} \Gamma_{i,r}^{B}\left(x\right) & =0, \end{array}$$

*then*

(65) $$\begin{array}{c} \Gamma_{i}\left(x\right)\leq e_{\mathrm{neg}}\left(r\right), \end{array}$$

*where*

(66) $$\begin{array}{c} e_{\mathrm{neg}}\left(r\right)=1-{\left(1-\rho_{\max}\right)}^{N_{<}}{\left(1-p_{r}\right)}^{N_{\max}\left(r\right)}. \end{array}$$

*Thus,*

(67) $$\begin{array}{c} 0\leq \Gamma_{i}\left(x\right)-\Gamma_{i,r}^{B}\left(x\right)\leq e_{\mathrm{neg}}\left(r\right). \end{array}$$

**Proof.**  Since $\Gamma_{i,r}^{B}\left(x\right)=0$, $T_{i}\left(x;r\right)<\tau_{\min}$, so at most $N_{<}$ relevant sensing opportunities occur inside the radius-*r* FOV. Their instantaneous detection probabilities are bounded by $\rho_{\max}$, giving an inner-region miss contribution of at least

(68) $$\begin{array}{c} {\left(1-\rho_{\max}\right)}^{N_{<}}. \end{array}$$

For sensing opportunities outside radius *r*, Assumption 1 gives $p\left(d_{k}\right)\leq p_{r}$, and Assumption 4 limits their number to $N_{\max}\left(r\right)$. Hence,

(69) $$\begin{array}{c} 1-\Gamma_{i}\left(x\right)\geq {\left(1-\rho_{\max}\right)}^{N_{<}}{\left(1-p_{r}\right)}^{N_{\max}\left(r\right)}. \end{array}$$

Rearranging gives (65). □

The first factor in (66) accounts for short dwell inside the binary radius; the second accounts for continuous sensing outside it.

A dwell threshold equal to one sampling period does not eliminate inner-region detection for binary-negative passages. A shorter passage can still contain one scheduled sample, depending on its phase.

**Corollary 1** 
(Binary-negative bound for a one-period dwell threshold)**.** *Under the assumptions of Lemma 2, if $\tau_{\min}=1/f$, then $N_{<}=1$, and every binary-negative passage satisfies*

(70) $$\Gamma_{i}\left(x\right)\leq 1-\left(1-\rho_{\max}\right){\left(1-p_{r}\right)}^{N_{\max}\left(r\right)}.$$

*In particular, when $r=R_{\mathrm{sens}}$,*

(71) $$\Gamma_{i}\left(x\right)\leq \rho_{\max}.$$

**Proof.**  Substitute $N_{<}=\left\lceil f\tau_{\min}\right\rceil =1$ into (66). At $r=R_{\mathrm{sens}}$, no outer-region sensing opportunities remain. □

For example, with f=1 Hz and sampling at integer seconds, an inner observation interval [0.9,1.1) fails a one-second dwell threshold but contains the sample at 1 s. Its detection probability can therefore be positive even when no outer sensing region remains. An outer-tail-only bound requires the additional condition that no inner sensing opportunity occurs; it does not follow from $\tau_{\min}=1/f$ alone.

**Remark 1** 
(Conservativeness of the binary-negative bound)**.** *The binary-negative bound can become conservative when $N_{<}$ is large, or $\rho_{\max}$ is close to one, because it permits every short-dwell sensing opportunity to occur at the maximum instantaneous detection probability. All subsequent results remain valid if $e_{\mathrm{neg}}\left(r\right)$ is replaced by a tighter trajectory- or geometry-specific upper bound.*

**Remark 2** 
(Two-sided binary sensing error)**.** *Binary-positive trajectories may be overestimated, whereas binary-negative trajectories may be underestimated. Increasing r reduces the ignored outer region but weakens the guaranteed sensing probability for binary-positive trajectories. Binary-radius calibration must therefore account for both effects.*

### 4.4. Network-Level Approximation Error

The preceding lemmas quantify the binary approximation error for a single sensor–trajectory pair. Sensor placement, however, is optimized at the network level, where overlapping sensing regions determine how individual errors combine. We therefore propagate the single-sensor bounds through the network missed-detection probability and ultimately to the deployment objective.

Define the signed per-sensor mismatch

(72) $$\begin{array}{c} \delta_{i,r}\left(x\right)=\Gamma_{i,r}^{B}\left(x\right)-\Gamma_{i}\left(x\right). \end{array}$$

Lemmas 1 and 2 imply

(73) $$\begin{array}{c} \left|\delta_{i,r}\left(x\right)\right|\leq {\bar{\delta }}_{i,r}\left(x\right), \end{array}$$

where

(74) $$\begin{array}{cc} {\bar{\delta }}_{i,r}\left(x\right)=\; & \Gamma_{i,r}^{B}\left(x\right)e_{\mathrm{in}}\left(r\right)\\ & +\left[1-\Gamma_{i,r}^{B}\left(x\right)\right]e_{\mathrm{neg}}\left(r\right). \end{array}$$

Define

(75) $$\begin{array}{cc} P_{r}\left(x;C\right) & =\left\{c_{i}\in C:\Gamma_{i,r}^{B}\left(x\right)=1\right\}. \end{array}$$

The corresponding network-level envelope is

(76) $$\begin{array}{c} E_{r}\left(x;C\right)=\left\{\begin{array}{cc} \prod_{c_{i}\in P_{r}\left(x;C\right)}e_{\mathrm{in}}\left(r\right), & P_{r}\left(x;C\right)\neq ⌀,\\ 1-{\left[1-e_{\mathrm{neg}}\left(r\right)\right]}^{\left|C\right|}, & P_{r}\left(x;C\right)=⌀. \end{array}\right. \end{array}$$

**Theorem 1** 
(Network missed-detection approximation bound)**.** *For every feasible deployment C and trajectory x,*

(77) $$\begin{array}{cc} \left|\Pi_{r}^{B}\left(x;C\right)-\Pi \left(x;C\right)\right| & \leq E_{r}\left(x;C\right). \end{array}$$

*Consequently,*

(78) $$\begin{array}{cc} \left|\Lambda_{r}^{B}\left(C\right)-\tilde{\Lambda }\left(C\right)\right| & \leq E(C,r), \end{array}$$

*where*

(79) $$\begin{array}{cc} E(C,r) & =\int_{\Theta }\bar{\lambda }\left(x\right)E_{r}\left(x;C\right)dx. \end{array}$$

**Proof.**  If $P_{r}\left(x;C\right)\neq ⌀$, then $\Pi_{r}^{B}\left(x;C\right)=0$. For each binary-positive sensor, Lemma 1 gives $1-\Gamma_{i}\left(x\right)\leq e_{\mathrm{in}}\left(r\right)$, while all remaining continuous miss probabilities are at most one. Hence,

(80) $$\begin{array}{cc} \Pi (x;C) & \leq \prod_{c_{i}\in P_{r}\left(x;C\right)}e_{\mathrm{in}}\left(r\right). \end{array}$$

If $P_{r}\left(x;C\right)=⌀$, then $\Pi_{r}^{B}\left(x;C\right)=1$. Lemma 2 gives $1-\Gamma_{i}\left(x\right)\geq 1-e_{\mathrm{neg}}\left(r\right)$ for every deployed sensor, so

(81) $$\begin{array}{cc} \Pi (x;C) & \geq {\left[1-e_{\mathrm{neg}}\left(r\right)\right]}^{\left|C\right|}. \end{array}$$

The two cases establish (77). Multiplication by $\bar{\lambda }\left(x\right)$ and integration over Θ yield (78). □

For comparison, the per-sensor envelope also gives the simpler additive bound

(82) $$\begin{array}{cc} \left|\Pi_{r}^{B}\left(x;C\right)-\Pi \left(x;C\right)\right| & \leq \min\left\{1,\;\sum_{c_{i}\in C}{\bar{\delta }}_{i,r}\left(x\right)\right\}. \end{array}$$

Theorem 1 is generally sharper because it preserves the binary-positive/binary-negative structure.

Theorem 1 bounds the error in missed-trajectory intensity. Because the deployment objective is expressed through the corresponding void probability, the following corollary translates this bound directly to the original deployment metric.

**Corollary 2** 
(Void-probability approximation interval)**.** *For every feasible deployment C,*

(83) $$\begin{array}{c} \nu_{r}^{B}\left(C\right)e^{-E(C,r)}\leq \tilde{\nu }\left(C\right)\leq \min\left\{1,\;\nu_{r}^{B}\left(C\right)e^{E(C,r)}\right\}. \end{array}$$

Thus, the sensing approximation is propagated from individual trajectories through network overlap to the deployment objective itself.

### 4.5. Binary-Radius Calibration

The preceding results quantify the binary sensing error for a fixed radius *r*. The radius itself, however, remains a design parameter. We therefore use the two-sided error structure to determine how *r* should be selected rather than prescribing it through an arbitrary instantaneous-detection threshold.

Because $p_{r}$ decreases with *r*, $e_{\mathrm{in}}\left(r\right)$ is nondecreasing. Under Assumption 4, $N_{\max}\left(r\right)$ is nonincreasing, so $e_{\mathrm{neg}}\left(r\right)$ is nonincreasing. A distribution-independent minimax radius is therefore

(84) $$\begin{array}{c} r_{\mathrm{mm}}=\underset{r\in R}{\mathrm{argmin}}\max\left\{e_{\mathrm{in}}\left(r\right),e_{\mathrm{neg}}\left(r\right)\right\}. \end{array}$$

**Proposition 2** 
(Balanced uniform radius)**.** *If $e_{\mathrm{in}}\left(r\right)$ and $e_{\mathrm{neg}}\left(r\right)$ are continuous on R and intersect at $r^{†}\in R$, then*

(85) $$\begin{array}{c} r_{\mathrm{mm}}=r^{†}. \end{array}$$

**Proof.**  For $r<r^{†}$, the decreasing error $e_{\mathrm{neg}}\left(r\right)$ is no smaller than its value at $r^{†}$. For $r>r^{†}$, the increasing error $e_{\mathrm{in}}\left(r\right)$ is no smaller than its value at $r^{†}$. Hence, the maximum of the two is minimized at their crossing. □

If the envelopes do not cross, or if a geometry-dependent $N_{\max}\left(r\right)$ makes the bound piecewise rather than continuous, (84) is evaluated directly over the admissible radius set. The minimax criterion controls the worst trajectory independently of how frequently that trajectory occurs. For deployment, this can be unnecessarily conservative: approximation errors in high-intensity portions of the trajectory domain affect the objective more strongly than comparable errors in rarely observed regions. We therefore introduce an intensity-weighted, deployment-aware criterion.

For candidate directional configuration *c*, define the intensity-weighted single-sensor approximation envelope as

(86) $$\begin{array}{c} e_{c}\left(r\right)=\int_{\Theta }\bar{\lambda }\left(x\right){\bar{\delta }}_{c,r}\left(x\right)dx. \end{array}$$

Because only one orientation may be selected at a physical location, define

(87) $$\begin{array}{c} e_{a}^{\max}\left(r\right)=\max_{\beta \in B}e_{\left(a,\beta \right)}\left(r\right). \end{array}$$

Let

(88) $$\begin{array}{c} e_{\left[1\right]}^{\max}\left(r\right)\geq e_{\left[2\right]}^{\max}\left(r\right)\geq \cdots \geq e_{\left[L\right]}^{\max}\left(r\right) \end{array}$$

denote these location-level values in nonincreasing order, and define

(89) $$\begin{array}{cc} E_{M}\left(r\right) & =\sum_{m=1}^{M}e_{\left[m\right]}^{\max}\left(r\right). \end{array}$$

**Corollary 3** 
(Deployment-aware radius calibration)**.** *For every feasible deployment C with |C|≤M and at most one sensor per physical location,*

(90) $$\begin{array}{c} \left|\Lambda_{r}^{B}\left(C\right)-\tilde{\Lambda }\left(C\right)\right|\leq E_{M}\left(r\right). \end{array}$$

*A deployment-aware radius is therefore*

(91) $$\begin{array}{c} r^{★}=\underset{r\in R}{\mathrm{argmin}}E_{M}\left(r\right). \end{array}$$

**Proof.**  Using (82),

(92) $$\begin{array}{c} \left|\Lambda_{r}^{B}\left(C\right)-\tilde{\Lambda }\left(C\right)\right|\leq \sum_{c_{i}\in C}e_{c_{i}}\left(r\right). \end{array}$$

A feasible deployment contains at most one configuration from each physical location, so this sum cannot exceed the *M* largest location-level maxima in (89). □

Unlike an instantaneous sensing threshold, (91) incorporates the sensing kernel, dwell requirement, sampling frequency, trajectory intensity, candidate geometry, sensor budget, and location-exclusivity constraint. It is therefore the radius-calibration criterion most directly aligned with the deployment decision.

### 4.6. Binary Marginal-Gain Distortion and Decision Preservation

The preceding results bound the difference between the binary and continuous deployment objectives. Greedy deployment, however, depends on the ordering of candidate marginal gains rather than on objective values alone. Consequently, an objective-error bound does not directly reveal whether the binary approximation changes the sensor selected by greedy optimization.

Define the continuous marginal gain

(93) $$\begin{array}{c} \Delta (c|C)=F(C\cup \{c\})-F(C). \end{array}$$

**Lemma 3** 
(Binary marginal-gain distortion)**.** *For every feasible partial deployment C and candidate c∉C,*

(94) $$\begin{array}{c} \left|\Delta_{r}^{B}\left(c|C\right)-\Delta \left(c|C\right)\right|\leq \varepsilon_{r}\left(c|C\right), \end{array}$$

*where*

(95) $$\begin{array}{c} \varepsilon_{r}\left(c|C\right)=E(C,r)+E(C\cup \{c\},r). \end{array}$$

**Proof.**  Using the missed-intensity representation of the two marginal gains,

(96) $$\begin{array}{c} \left|\Delta_{r}^{B}\left(c|C\right)-\Delta \left(c|C\right)\right|\\ \leq \left|\Lambda_{r}^{B}\left(C\right)-\tilde{\Lambda }\left(C\right)\right|\\ \;\;\;+\left|\Lambda_{r}^{B}\left(C\cup \left\{c\right\}\right)-\tilde{\Lambda }\left(C\cup \left\{c\right\}\right)\right|. \end{array}$$

Theorem 1 gives the result. □

Lemma 3 bounds the distortion of each candidate marginal gain individually. We next translate this candidate-wise error into a guarantee on the candidate actually selected by binary greedy. At iteration *t*, let $V_{t}$ denote the feasible candidate set and define

(97) $$\begin{array}{c} \varepsilon_{t}\left(r\right)=\max_{c\in V_{t}}\varepsilon_{r}\left(c|C_{t-1}\right). \end{array}$$

**Corollary 4** 
(Approximate binary greedy step)**.** *If*

(98) $$\begin{array}{c} c_{t}^{B}=\underset{c\in V_{t}}{\mathrm{argmax}}\Delta_{r}^{B}\left(c|C_{t-1}\right), \end{array}$$

*then*

(99) $$\begin{array}{c} \Delta \left(c_{t}^{B}|C_{t-1}\right)\geq \max_{c\in V_{t}}\Delta \left(c|C_{t-1}\right)-2\varepsilon_{t}\left(r\right). \end{array}$$

**Proof.**  Let

$$c_{t}^{★}=\underset{c\in V_{t}}{\mathrm{argmax}}\Delta \left(c|C_{t-1}\right).$$

By Lemma 3,

(100) $$\begin{array}{cc} \Delta (c_{t}^{B}|C_{t-1}) & \geq \Delta_{r}^{B}\left(c_{t}^{B}|C_{t-1}\right)-\varepsilon_{t}\left(r\right)\\ & \geq \Delta_{r}^{B}\left(c_{t}^{★}|C_{t-1}\right)-\varepsilon_{t}\left(r\right)\\ & \geq \Delta \left(c_{t}^{★}|C_{t-1}\right)-2\varepsilon_{t}\left(r\right). \end{array}$$

□

The preceding corollary guarantees that the binary-selected candidate is close to the best continuous candidate, but it does not guarantee that the two argmax decisions are identical. Exact agreement can be certified when the bounded marginal-gain intervals of the competing candidates do not overlap.

**Corollary 5** 
(Candidate-specific binary decision certificate)**.** *Suppose binary greedy selects $c_{t}^{B}$. If*

(101) $$\begin{array}{cc} & \Delta_{r}^{B}\left(c_{t}^{B}|C_{t-1}\right)-\varepsilon_{r}\left(c_{t}^{B}|C_{t-1}\right)\\ & \;>\max_{c\in V_{t}\setminus \left\{c_{t}^{B}\right\}}\left[\Delta_{r}^{B}\left(c|C_{t-1}\right)+\varepsilon_{r}\left(c|C_{t-1}\right)\right], \end{array}$$

*then $c_{t}^{B}$ also maximizes the continuous marginal gain.*

A simpler sufficient condition follows from the uniform iteration-level distortion.

**Corollary 6** 
(Exact binary decision certificate)**.** *Let $c_{t}^{\left(1\right)}$ and $c_{t}^{\left(2\right)}$ denote the candidates with the largest and second-largest binary marginal gains. If*

(102) $$\begin{array}{c} \Delta_{r}^{B}\left(c_{t}^{\left(1\right)}|C_{t-1}\right)-\Delta_{r}^{B}\left(c_{t}^{\left(2\right)}|C_{t-1}\right)>2\varepsilon_{t}\left(r\right), \end{array}$$

*then $c_{t}^{\left(1\right)}$ also maximizes the continuous marginal gain.*

These results emphasize that the binary model need not reproduce every continuous objective value accurately. For greedy deployment, it is sufficient to preserve the ordering of the highest-value candidates.

### 4.7. Omni-Greedy Location–Orientation Approximation

The preceding analysis isolates the first approximation: replacing continuous sensing by the binary model while retaining a full directional search. Even if the binary approximation were exact, omni-greedy introduces a second and distinct source of error by ranking physical locations before optimizing their orientations. We quantify this location–orientation decoupling.

For partial deployment *C*, define the best binary directional value available at location *a* as

(103) $$\begin{array}{c} G_{r}\left(a|C\right)=\max_{\beta \in B}\Delta_{r}^{B}\left((a,\beta )|C\right). \end{array}$$

Recall the scaled omnidirectional score

(104) $$\begin{array}{c} {\tilde{H}}_{r}\left(a|C\right)=\frac{\phi }{\pi }\Delta_{r}^{O}\left(a|C\right). \end{array}$$

The factor ϕ/π does not affect location ranking; it places the omnidirectional score on the angular scale of a directional sector of width 2ϕ.

Because the optimization uses a finite orientation set, we analyze directional variation directly on that set.

**Assumption 5** 
(Uniform orientation grid for harmonic analysis)**.** *The candidate orientations are uniformly spaced:*

(105) $$\begin{array}{c} \beta^{\left(j\right)}=\beta_{0}+\frac{2\pi j}{K},\;j=0,\ldots ,K-1. \end{array}$$

For fixed *a*, *C*, and *r*, define

(106) $$\begin{array}{c} g_{j}\left(a,C,r\right)=\Delta_{r}^{B}\left((a,\beta^{\left(j\right)})|C\right). \end{array}$$

Its discrete Fourier coefficients are

(107) $$\begin{array}{c} {\hat{g}}_{k}\left(a,C,r\right)=\frac{1}{K}\sum_{j=0}^{K-1}g_{j}\left(a,C,r\right)\exp\left(-\frac{2\pi ijk}{K}\right), \end{array}$$

with exact inverse representation

(108) $$\begin{array}{c} g_{j}\left(a,C,r\right)=\sum_{k=0}^{K-1}{\hat{g}}_{k}\left(a,C,r\right)\exp\left(\frac{2\pi ijk}{K}\right). \end{array}$$

The zero-frequency coefficient is the orientation-averaged directional gain,

(109) $$\begin{array}{cc} {\bar{G}}_{r}\left(a|C\right) & ={\hat{g}}_{0}\left(a,C,r\right)\\ & =\frac{1}{K}\sum_{j=0}^{K-1}g_{j}\left(a,C,r\right). \end{array}$$

Define the finite harmonic anisotropy envelope

(110) $$\begin{array}{c} A_{r}\left(a,C\right)=\sum_{k=1}^{K-1}\left|{\hat{g}}_{k}\left(a,C,r\right)\right|. \end{array}$$

Then,

(111) $$\begin{array}{c} \left|g_{j}\left(a,C,r\right)-{\bar{G}}_{r}\left(a|C\right)\right|\leq A_{r}\left(a,C\right) \end{array}$$

for every candidate orientation.

The second source of discrepancy is the difference between the orientation-averaged directional gain and the scaled omnidirectional score:

(112) $$\begin{array}{c} B_{r}\left(a,C\right)=\left|{\bar{G}}_{r}\left(a|C\right)-{\tilde{H}}_{r}\left(a|C\right)\right|. \end{array}$$

Thus, $A_{r}\left(a,C\right)$ measures sensitivity to orientation, whereas $B_{r}\left(a,C\right)$ measures the mismatch between omnidirectional exposure and orientation-averaged directional value.

To interpret $B_{r}$, define the residual trajectory weight

(113) $$\begin{array}{c} w_{C}\left(x\right)=\bar{\lambda }\left(x\right)\Pi_{r}^{B}\left(x;C\right) \end{array}$$

and the fraction of candidate orientations at location *a* that binary-detect trajectory *x*:

(114) $$\begin{array}{c} q_{a,r}\left(x\right)=\frac{1}{K}\sum_{j=0}^{K-1}\Gamma_{(a,\beta^{\left(j\right)}),r}^{B}\left(x\right). \end{array}$$

Then,

(115) $$\begin{array}{c} {\bar{G}}_{r}\left(a|C\right)=\int_{\Theta }w_{C}\left(x\right)q_{a,r}\left(x\right)dx. \end{array}$$

Since every directional sector lies inside its corresponding omnidirectional disk, $q_{a,r}\left(x\right)=0$ whenever $\Gamma_{a,r}^{O}\left(x\right)=0$. Therefore,

(116) $$\begin{array}{c} B_{r}\left(a,C\right)=\left|\int_{\Theta }w_{C}\left(x\right)\Gamma_{a,r}^{O}\left(x\right)\left[q_{a,r}\left(x\right)-\frac{\phi }{\pi }\right]dx\right|. \end{array}$$

Equation (116) shows that ϕ/π would be an exact trajectory-level scaling only if an omnidirectionally exposed trajectory were detected by exactly that fraction of candidate orientations. FOV geometry, trajectory direction, and the dwell-time criterion generally prevent this equality.

The best directional value can therefore differ from the omnidirectional surrogate for two distinct reasons: variation of the gain across orientations, captured by $A_{r}$, and mismatch between the omnidirectional score and the orientation-averaged directional gain, captured by $B_{r}$. The following theorem combines these two effects into a location-level approximation bound.

**Theorem 2** 
(Omnidirectional-to-directional location gap)**.** *Under Assumption 5,*

(117) $$\begin{array}{cc} \left|G_{r}\left(a|C\right)-{\tilde{H}}_{r}\left(a|C\right)\right| & \leq A_{r}\left(a,C\right)+B_{r}\left(a,C\right). \end{array}$$

**Proof.**  Since $G_{r}\left(a|C\right)=\max_{j}g_{j}\left(a,C,r\right)$ and ${\bar{G}}_{r}\left(a|C\right)$ is the average of the same finite set,

(118) $$\begin{array}{c} 0\leq G_{r}\left(a|C\right)-{\bar{G}}_{r}\left(a|C\right)\leq A_{r}\left(a,C\right). \end{array}$$

Applying the triangle inequality with (112) gives the result. □

Theorem 2 bounds the surrogate error at a fixed location. Omni-greedy, however, uses these surrogate values to choose among multiple locations. We therefore convert the location-wise approximation error into a bound on the loss incurred by the location actually selected by omni-greedy.

Define

(119) $$\begin{array}{c} Z_{r}\left(a,C\right)=A_{r}\left(a,C\right)+B_{r}\left(a,C\right), \end{array}$$

and at iteration *t*,

(120) $$\begin{array}{c} \zeta_{t}\left(r\right)=\max_{a\in L\setminus L(C_{t-1})}Z_{r}\left(a,C_{t-1}\right). \end{array}$$

**Corollary 7** 
(One-step omni-greedy loss)**.** *If*

(121) $$\begin{array}{cc} a_{t}^{\mathrm{OG}} & =\underset{a\in L\setminus L(C_{t-1})}{\mathrm{argmax}}{\tilde{H}}_{r}\left(a|C_{t-1}\right), \end{array}$$

*then*

(122) $$\begin{array}{c} G_{r}\left(a_{t}^{\mathrm{OG}}|C_{t-1}\right)\geq \max_{a}G_{r}\left(a|C_{t-1}\right)-2\zeta_{t}\left(r\right), \end{array}$$

*where the maximum is over feasible unoccupied locations.*

**Proof.**  Let

$$a_{t}^{\mathrm{DG}}=\underset{a}{\mathrm{argmax}}G_{r}\left(a|C_{t-1}\right),$$

where the maximum is over feasible unoccupied locations. By Theorem 2,

(123) $$\begin{array}{cc} G_{r}\left(a_{t}^{\mathrm{OG}}|C_{t-1}\right) & \geq {\tilde{H}}_{r}\left(a_{t}^{\mathrm{OG}}|C_{t-1}\right)-\zeta_{t}\left(r\right)\\ & \geq {\tilde{H}}_{r}\left(a_{t}^{\mathrm{DG}}|C_{t-1}\right)-\zeta_{t}\left(r\right)\\ & \geq G_{r}\left(a_{t}^{\mathrm{DG}}|C_{t-1}\right)-2\zeta_{t}\left(r\right). \end{array}$$

□

The one-step bound controls the loss in location value but does not imply that omni-greedy selects exactly the same location as full directional greedy. Exact agreement can again be certified when the lower surrogate bound of the selected location exceeds the upper bound of every competitor.

**Corollary 8** 
(Exact omni-greedy location certificate)**.** *Suppose omni-greedy selects $a_{t}^{\mathrm{OG}}$. If*

(124) $$\begin{array}{cc} & {\tilde{H}}_{r}\left(a_{t}^{\mathrm{OG}}|C_{t-1}\right)-Z_{r}\left(a_{t}^{\mathrm{OG}},C_{t-1}\right)\\ & \;>{\tilde{H}}_{r}\left(a|C_{t-1}\right)+Z_{r}\left(a,C_{t-1}\right) \end{array}$$

*for every competing feasible location $a\neq a_{t}^{\mathrm{OG}}$, then $a_{t}^{\mathrm{OG}}$ also maximizes $G_{r}\left(a|C_{t-1}\right)$.*

Thus, omni-greedy is reliable when the separation among omnidirectional location scores is large relative to the combined orientation-anisotropy and surrogate discrepancies.

**Remark 3** 
(Nonuniform orientation candidates)**.** *For a nonuniform orientation set, the Fourier representation may be replaced by*

(125) $$\begin{array}{cc} A_{r}^{\mathrm{dir}}\left(a,C\right) & =\max_{\beta \in B}\left|\Delta_{r}^{B}\left(\left(a,\beta \right)|C\right)-{\bar{G}}_{r}\left(a|C\right)\right|, \end{array}$$

*and the subsequent results remain unchanged with $A_{r}^{\mathrm{dir}}$ in place of $A_{r}$.*

### 4.8. Combined Binary Omni-Greedy Step

The two approximation mechanisms have so far been analyzed separately: $\varepsilon_{t}\left(r\right)$ quantifies continuous-to-binary sensing distortion, whereas $\zeta_{t}\left(r\right)$ quantifies location–orientation decoupling. Binary omni-greedy incurs both approximations simultaneously, so we next combine their one-step effects. Let

(126) $$\begin{array}{c} c_{t}^{B-\mathrm{OG}}=\left(a_{t}^{\mathrm{OG}},\beta_{t}^{\mathrm{OG}}\right) \end{array}$$

denote the sensor selected by binary omni-greedy at iteration *t*.

**Theorem 3** 
(Continuous marginal gain of binary omni-greedy)**.** *At every iteration,*

(127) $$\begin{array}{cc} & \Delta \left(c_{t}^{B-\mathrm{OG}}|C_{t-1}\right)\\ & \;\geq \max_{c\in V_{t}}\Delta \left(c|C_{t-1}\right)-2\varepsilon_{t}\left(r\right)-2\zeta_{t}\left(r\right). \end{array}$$

**Proof.**  Lemma 3 gives

(128) $$\begin{array}{c} \Delta \left(c_{t}^{B-\mathrm{OG}}|C_{t-1}\right)\geq \Delta_{r}^{B}\left(c_{t}^{B-\mathrm{OG}}|C_{t-1}\right)-\varepsilon_{t}\left(r\right). \end{array}$$

Because omni-greedy optimizes orientation at its selected location,

(129) $$\begin{array}{c} \Delta_{r}^{B}\left(c_{t}^{B-\mathrm{OG}}|C_{t-1}\right)=G_{r}\left(a_{t}^{\mathrm{OG}}|C_{t-1}\right). \end{array}$$

Corollary 7 therefore implies

(130) $$\begin{array}{c} \Delta_{r}^{B}\left(c_{t}^{B-\mathrm{OG}}|C_{t-1}\right)\geq \max_{c\in V_{t}}\Delta_{r}^{B}\left(c|C_{t-1}\right)-2\zeta_{t}\left(r\right). \end{array}$$

Finally,

(131) $$\begin{array}{c} \max_{c\in V_{t}}\Delta_{r}^{B}\left(c|C_{t-1}\right)\geq \max_{c\in V_{t}}\Delta \left(c|C_{t-1}\right)-\varepsilon_{t}\left(r\right). \end{array}$$

Combining the inequalities gives (127). □

The one-step approximation loss is therefore separated into

(132) $$\begin{array}{c} \underset{\mathrm{binary}\;\mathrm{sensing}\;\mathrm{distortion}}{\underset{︸}{2\varepsilon_{t}\left(r\right)}}+\underset{\mathrm{location}–\mathrm{orientation}\;\mathrm{decoupling}}{\underset{︸}{2\zeta_{t}\left(r\right)}}. \end{array}$$

The first term is controlled primarily by binary-radius calibration, whereas the second is controlled by directional anisotropy and the fidelity of the omnidirectional location surrogate.

### 4.9. End-to-End Deployment Guarantee

Theorem 3 bounds the approximation loss at a single greedy iteration. Final deployment performance, however, depends on how these losses accumulate over all *M* sensor selections. We therefore combine the one-step result with the submodular structure established in Proposition 1 and the location-exclusivity constraint to obtain an end-to-end guarantee.

Let

(133) $$\begin{array}{c} V=L\times B \end{array}$$

be the directional candidate set, and let I contain all subsets C⊆V satisfying |C|≤M and containing at most one orientation associated with each physical location. The feasible family (V,I) is a rank-*M* truncation of a partition matroid.

Define the optimal deterministic continuous deployment as

(134) $$\begin{array}{c} C^{★}=\underset{C\in I}{\mathrm{argmax}}F\left(C\right). \end{array}$$

**Theorem 4** 
(End-to-end performance under location exclusivity)**.** *Suppose at least M distinct candidate locations are available. Let $C_{M}^{B-\mathrm{OG}}$ denote the M-sensor deployment produced by binary omni-greedy. Then,*

(135) $$\begin{array}{c} F\left(C_{M}^{B-\mathrm{OG}}\right)\geq \frac{1}{2}F\left(C^{★}\right)-\sum_{t=1}^{M}\left[\varepsilon_{t}\left(r\right)+\zeta_{t}\left(r\right)\right]. \end{array}$$

**Proof.**  By Proposition 1, *F* is normalized, monotone, and submodular. Because at least *M* distinct candidate locations are available and *F* is monotone, an optimal feasible deployment may be taken to contain *M* sensors. Since (V,I) is a matroid, the exchange property permits the elements of such an optimal deployment to be ordered as $o_{1},\ldots ,o_{M}$ so that $o_{t}$ is feasible to add to $C_{t-1}$.

By Theorem 3,

(136) $$\begin{array}{c} \Delta (o_{t}|C_{t-1})\leq \Delta \left(c_{t}^{B-\mathrm{OG}}|C_{t-1}\right)+2\varepsilon_{t}\left(r\right)+2\zeta_{t}\left(r\right). \end{array}$$

Monotonicity and submodularity give

(137) $$\begin{array}{c} F(C^{★})\leq F\left(C_{M}^{B-\mathrm{OG}}\right)+\sum_{t=1}^{M}\Delta \left(o_{t}|C_{t-1}\right). \end{array}$$

Applying (136) and using the telescoping identity

(138) $$\begin{array}{c} \sum_{t=1}^{M}\Delta \left(c_{t}^{B-\mathrm{OG}}|C_{t-1}\right)=F\left(C_{M}^{B-\mathrm{OG}}\right) \end{array}$$

yields

(139) $$\begin{array}{cc} F(C^{★}) & \leq 2F\left(C_{M}^{B-\mathrm{OG}}\right)+2\sum_{t=1}^{M}\left[\varepsilon_{t}\left(r\right)+\zeta_{t}\left(r\right)\right]. \end{array}$$

Rearranging proves (135). □

#### Scope of the Approximation Guarantee

The preceding result is an approximation guarantee for the stated finite candidate problem, rather than a convergence result to its global optimum. Even when the sensing and search error terms vanish, the underlying matroid-constrained greedy guarantee does not imply that the achieved objective value approaches the optimum. The additive terms quantify further allowances associated with the two approximations and may render the lower bound uninformative. Accordingly, near-optimal empirical performance can be asserted only for instances on which a sufficiently small optimality gap is established against an exact optimum or a valid upper bound. The result does not establish global optimality over continuous sensor locations and orientations. The factor 1/2 is the baseline worst-case guarantee for simple greedy maximization of a monotone submodular objective under the location-exclusivity matroid constraint. The additive terms isolate the additional degradation introduced by the two approximations proposed in this paper.

Theorem 4 is expressed in terms of the submodular benefit F(C) because that is the natural quantity for greedy analysis. The original deployment metric, however, is the deterministic void probability. The following corollary translates the same guarantee back to that metric.

**Corollary 9** 
(End-to-end deterministic void-probability guarantee)**.** *Define*

(140) $$\begin{array}{c} S_{M}\left(r\right)=\sum_{t=1}^{M}\left[\varepsilon_{t}\left(r\right)+\zeta_{t}\left(r\right)\right]. \end{array}$$

*Then,*

(141) $$\begin{array}{c} \tilde{\Lambda }\left(C_{M}^{B-\mathrm{OG}}\right)\leq \frac{\tilde{\Lambda }\left(⌀\right)+\tilde{\Lambda }\left(C^{★}\right)}{2}+S_{M}\left(r\right), \end{array}$$

*and*

(142) $$\begin{array}{c} \tilde{\nu }\left(C_{M}^{B-\mathrm{OG}}\right)\geq e^{-S_{M}\left(r\right)}\sqrt{\tilde{\nu }\left(⌀\right)\tilde{\nu }\left(C^{★}\right)}. \end{array}$$

**Proof.**  Substituting $F\left(C\right)=\tilde{\Lambda }\left(⌀\right)-\tilde{\Lambda }\left(C\right)$ into Theorem 4 and rearranging gives (141). Exponentiating its negative yields (142). □

### 4.10. Summary of the Theoretical Results

The analysis distinguishes two approximation mechanisms. The binary sensing term $\varepsilon_{t}\left(r\right)$ characterizes marginal-gain distortion caused by replacing accumulated probabilistic detection with a dwell-time-aware binary response. The location–orientation term $\zeta_{t}\left(r\right)$ characterizes the discrepancy associated with using an omnidirectional location proxy in place of the best directional score. Separating these mechanisms clarifies whether a deployment discrepancy originates from the sensing approximation or from the reduced search.

At the sensing level, a binary-positive passage satisfies the minimum dwell-time criterion but may still be missed by the probabilistic detector. Conversely, a binary-negative passage may retain a nonzero detection probability despite failing that criterion. The two-sided error bounds account for these distinct discrepancies and propagate them to the network objective. Radius calibration uses these bounds to balance the effects of excluding potentially detectable passages and assigning perfect detection to imperfectly observed passages, while accounting for trajectory intensity and deployment constraints.

The decision-preservation results explain why an approximate sensing model can select the same configuration as the probabilistic model without reproducing its objective values exactly. Agreement can be certified when the separation between competing marginal gains is sufficiently large relative to the approximation bounds. When this condition fails, the selected configurations may still agree; the result simply does not certify that agreement. Moreover, different selections can have similar detection benefits when several candidates have nearly equal marginal gains.

For omni-greedy, the physical distinction is between exposure within an omnidirectional disk and exposure that can be captured by one directional sector. A location with substantial disk-based exposure need not admit an orientation that captures a comparable amount. The location-score analysis therefore accounts for both directional anisotropy and mismatch between the scaled omnidirectional score and the orientation-averaged directional score. These comparisons describe candidate scores at a common partial deployment; they do not, by themselves, bound the final objective difference between two algorithms following different deployment sequences.

Finally, (135) combines the approximation terms with the greedy guarantee associated with the submodular objective and matroid feasibility constraints. Its scope is the specified reference objective over the finite candidate set, rather than unrestricted continuous placement or the full posterior-averaged LGCP objective. The bound may be conservative or uninformative when the additive error terms are large, even when empirical performance is strong. Section 5 therefore evaluates observed sensing and deployment discrepancies alongside the conservativeness of the theoretical bounds on the maritime and wildlife datasets.

## 5. Results

The proposed framework is evaluated using two real trajectory datasets: maritime AIS vessel traffic near the Savannah Harbor approach and GPS telemetry from mule deer. Both studies use the directional trajectory domain Θ=[0,2π)×[−1,1] and the same definitions of sensing, approximation error, and deployment performance. Length is normalized by the monitoring-region radius $L_{\mathrm{ref}}$.

The sensing parameters are dataset-specific because the two applications represent different physical sensors and target dynamics. The maritime study uses a wide-sector sensor with a 1 Hz revisit, whereas the wildlife study uses a narrow-sector thermal imager with a 10 min revisit. Normalized detected intensity is used as the primary scale-independent performance measure. Void probability is additionally reported using a specified observation window for each dataset and is interpreted only within that dataset.

In both studies, the planning intensity $\bar{\lambda }\left(x\right)$ is constructed from the training trajectories using a kernel density estimate that is periodic in heading and weighted by segment length. No LGCP posterior is fitted. The experiments therefore evaluate deployment performance conditional on a deterministic estimated intensity, rather than performance under posterior uncertainty in that intensity.

This choice isolates the sensing and search approximations analyzed in Section 4, whose guarantees require a fixed nonnegative planning intensity but do not require a particular intensity-estimation procedure. The reported void probability $\tilde{\nu }\left(C\right)$ is evaluated under the deterministic Poisson model associated with this estimate. It is not an evaluation of the posterior-averaged LGCP probability ν(C), and the tightness of the Jensen bound in (22) is not assessed.

The experiments are organized to mirror the theoretical development in Section 4. We first examine binary-radius calibration and the trajectory-level approximation bounds, then evaluate how binary sensing affects network objectives and greedy decisions. We next quantify the location–orientation error introduced by omni-greedy, followed by end-to-end deployment performance, held-out evaluation, and computational scaling. The corresponding theoretical results are referenced where each quantity is evaluated.

Table 3 summarizes the dataset, deployment, and sensing settings for both case studies. Dataset preprocessing and parameter-selection details are described below.

### 5.1. Parameter Selection and Interpretation

The sensing parameters were selected as fixed settings for the numerical case studies; they were not decision variables in the deployment optimization. They define illustrative probabilistic sensing models evaluated on real trajectory data and are not claimed to be calibrated descriptions of a particular physical sensor. Their role is to provide a common reference for assessing the sensing and search approximations under each scenario.

The binary radius $r^{★}$ is treated separately: it is selected through the error-envelope calibration in Section 4.5 after the reference sensing parameters have been specified. Consequently, radius calibration approximates the chosen detector model rather than identifying its physical parameters. The reported performance should be interpreted conditional on these sensing assumptions. In Table 3, (D) marks values supported by data, (S) values taken from a sensor specification, and (A) modeling assumptions; for the maritime study, $\rho_{\max}$, ϕ, *f*, $R_{\mathrm{sens}}$, $\tau_{\min}$ and the observation window are assumed. For the wildlife study, the trajectories are GPS fixes of 54 collared mule deer recorded at 5 h intervals between January 2008 and May 2010 [9]; their preprocessing is described in Section 5.3. The sensor geometry follows the specification of the modeled thermal imager [10]: its 640-pixel detector with 12-μm pitch and a 35 mm lens gives $\phi =6.26^{\circ }$, and the Johnson detection criterion, two pixels across the critical dimension of a deer-sized target [11], gives $R_{\mathrm{sens}}=2052$ m (S). The Johnson criterion, as used in current range specifications of thermal imagers, defines this range as the one at which the detection probability is 0.5 [11,12]. The distance scale is therefore set by $\rho_{\max}y\left(R_{\mathrm{sens}};l\right)=0.5$, which gives l=0.199 (S); a fit to the target transfer probability function of the Johnson-criteria acquisition model [13] gives 0.214. The speed used for dwell time, the KDE bandwidths and the expected number of passages are estimated from the training data (D), whereas $\rho_{\max}$, *f*, $\tau_{\min}$ and the observation window are assumed (A). Table 4 lists every parameter of the wildlife study with its source and the reason or reference for its value; (N) marks numerical resolutions fixed by a convergence study.

### 5.2. Numerical Implementation of the Reference Detector

**Figure 3:**
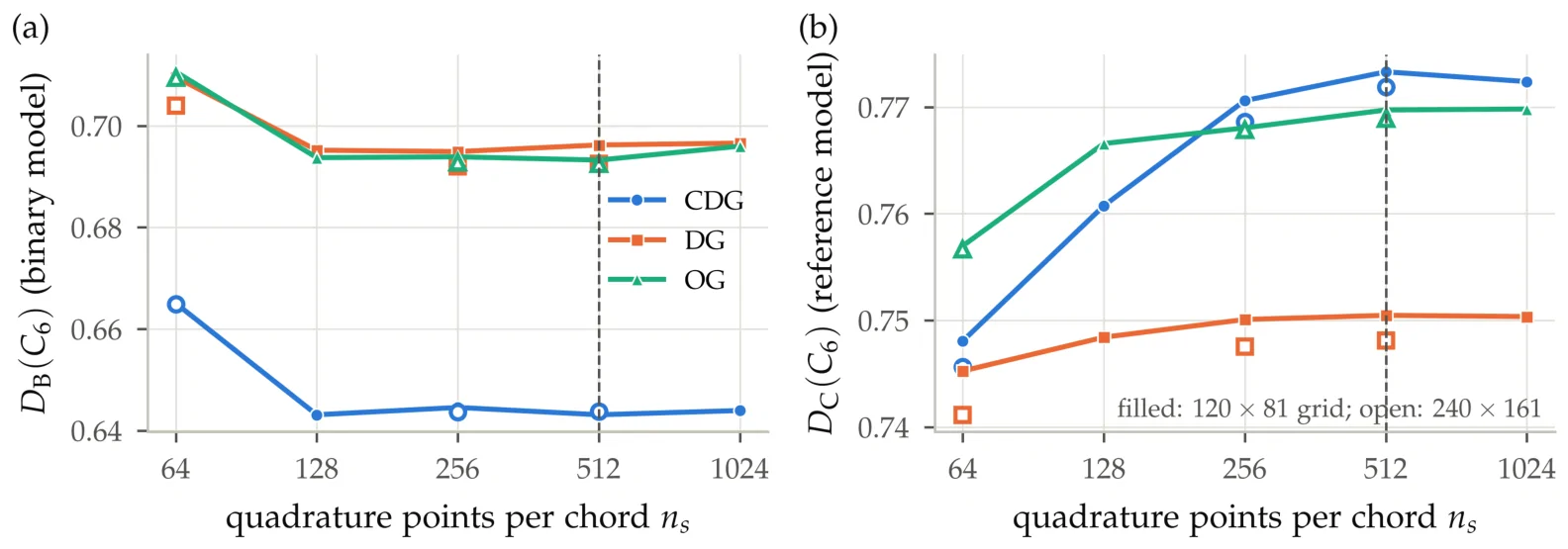
Numerical convergence of the wildlife detector for the M=6 deployments of each method. (**a**) Normalized detected intensity under the binary model versus quadrature points per chord. (**b**) The same under the probabilistic reference model. Filled markers: 120×81Θ grid; open markers: 240×161; dashed line: 512 points, used in this study.

Each heading–offset quadrature node is evaluated as an unbounded directed line; no finite passage interval, entry time, or per-trajectory speed is assigned to a node. For each sensor, the line is intersected with the disk of radius $R_{\mathrm{sens}}$ and sampled at 64 equally spaced points, which are retained when they lie in $F_{i}$. Intersection length is converted to time by dividing by a single constant speed, the median speed of the training segments (5.56 m/s for the maritime data). The binary surrogate declares detection when the resulting in-footprint time within radius *r* is at least $\tau_{\min}$; because $F_{i}\left(r\right)$ is convex for ϕ≤π/2, this time is the length of a single interval. The probabilistic reference detector does not simulate sampling times: it assigns $\left\lfloor f\;l_{\mathrm{disk}}\left(x\right)/v\right\rfloor$ sensing opportunities, where $l_{\mathrm{disk}}\left(x\right)$ is the chord length through the disk of radius $R_{\mathrm{sens}}$, distributes them uniformly over the chord points, and retains those inside $F_{i}$. The resulting number of in-footprint opportunities is a phase-free approximation of (12) and need not be an integer. The sample-count bounds of Section 4 are stated for the scheduled model of (12); the numerical detector serves as a common reference for comparing deployments under the stated assumptions. The numerical experiments therefore use a phase-free approximation to the scheduled detector. The sample-count bounds in Section 4 apply to the periodically scheduled model defined; they are theoretical results for that model and are not direct certificates for the phase-free numerical detector used in the experiments. The wildlife study uses the same implementation with 512 points per chord and $\bar{v}=0.0183$ m/s. Its 120×81 nodes are the midpoints of heading–offset cells, $\Delta \xi =3^{\circ }$ and $\Delta \tilde{p}=2/81$, each weighted by $w_{j}=\bar{\lambda }\left(x_{j}\right)\;\Delta \xi \;\Delta \tilde{p}$. A check against scheduled samples at ϑ+k/f with explicit phase, counting each in-footprint interval separately, agrees with the phase-free detector to within 0.003 for 95% of 3000 sampled candidate–trajectory pairs; the largest differences occur for passages that only graze a sector. The larger number of points is needed because deer are slow: the dwell threshold corresponds to only $\bar{v}\tau_{\min}=110$ m of path inside a sector, whereas 64 points on a horizon chord of up to $2R_{\mathrm{sens}}=4.1$ km are 64 m apart, so a passage’s binary label would be decided by one or two points. For the same six-sensor deployments, the binary detected fraction changes by up to 0.022 between 64 and 128 points, but by at most 0.003 (binary and reference) between 512 and 1024 points; refining the Θ grid to 240×161 changes it by at most 0.004 (Figure 3).

### 5.3. Trajectory Data and Directional Representation

#### 5.3.1. Maritime

June 2022 AIS records from MarineCadastre were restricted to a monitoring region of radius $L_{\mathrm{ref}}=10$ km centered on the Tybee Roads entrance ($31.99^{\circ }$ N, $80.80^{\circ }$ W) [18]. Cargo and tanker traffic was retained after removing moored and low-speed records. Headings were computed from the temporal direction of travel, and curved tracks were divided into approximately linear segments at heading changes exceeding $20^{\circ }$. The resulting dataset contains 190 segments from 82 vessels. Splitting by vessel gives 144 training segments from 57 vessels and 46 testing segments from 25 vessels, with no vessel shared between the two sets. Although AIS provides a speed for each segment, the detection calculation uses the median training speed for every passage.

**Figure 4:**
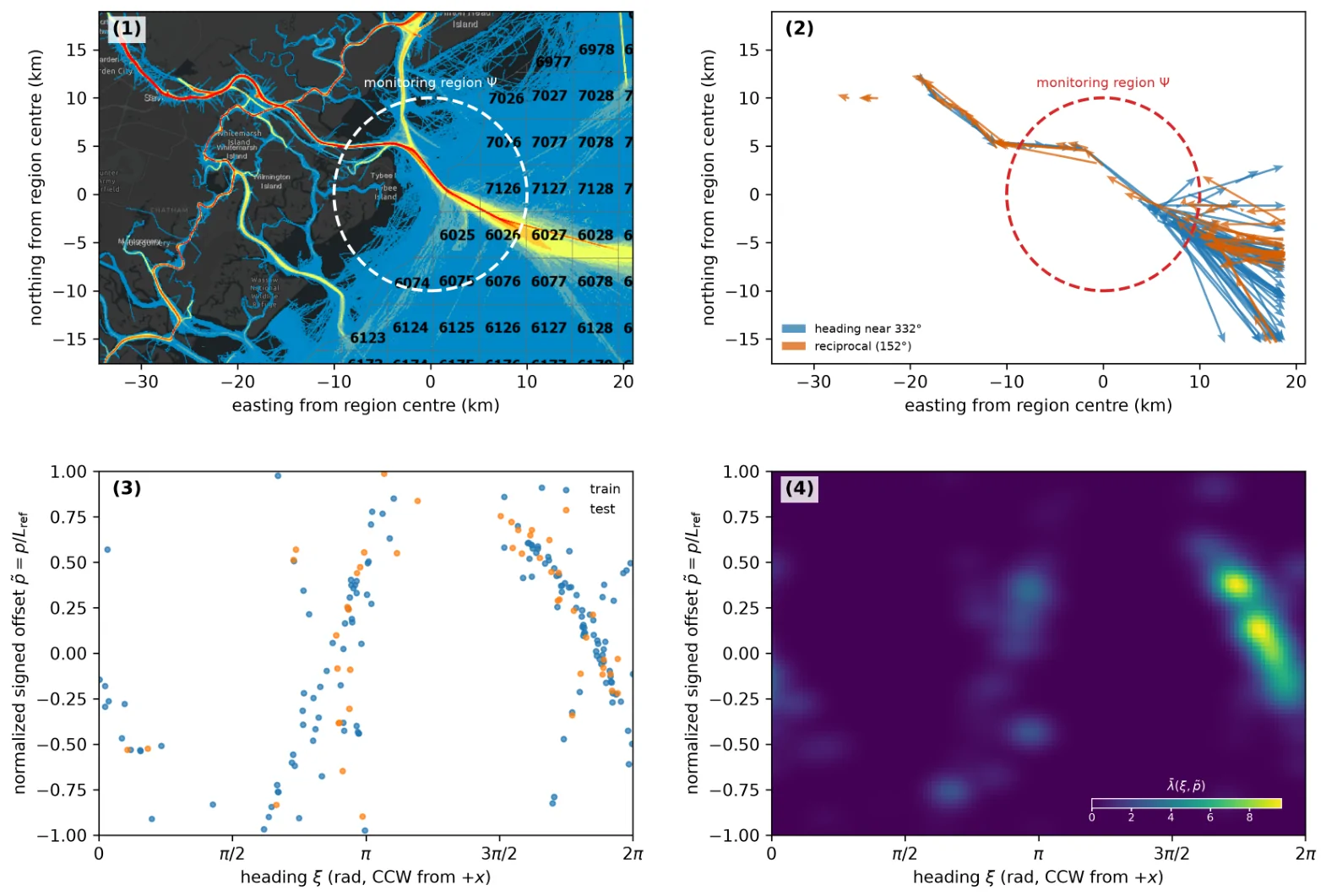
Directional trajectory representation for the Savannah Harbor approach. (**1**) Published 2022 vessel transit density from MarineCadastre AccessAIS [18] and the monitoring region Ψ, with $L_{\mathrm{ref}}=10$ km. (**2**) Extracted directed vessel segments. (**3**) Segments mapped to Θ=[0,2π)×[−1,1], where reciprocal flows remain distinct. (**4**) KDE estimate of the directional trajectory intensity $\bar{\lambda }\left(\xi ,\tilde{p}\right)$ from the training data.

Figure 4 summarizes the complete representation process. The extracted segments reproduce the dredged-channel traffic and offshore approach fan, with dominant directions near $332^{\circ }$ and $152^{\circ }$. Most importantly, inbound and outbound transits appear in the trajectory domain as the reciprocal pair $\left(\xi ,\tilde{p}\right)$ and $\left(\xi +\pi ,-\tilde{p}\right)$. The estimated intensity retains separate inbound and outbound occurrence statistics. This distinction describes the traffic distribution, but does not by itself imply different detection responses or improved deployment performance relative to aggregating reciprocal passages. The mean normalized offset magnitude is 0.404, and the most represented vessel contributes 25 of the 190 segments.

#### 5.3.2. Wildlife

The wildlife dataset contains 33,634 GPS fixes from 54 collared mule deer (*Odocoileus hemionus*) collected between January 2008 and May 2010.

Approximately 96.7% of inter-fix intervals are 5 h, whereas the modeled sensor samples every 10 min. The intermediate positions used for detection evaluation are therefore inferred under the locally linear, constant-speed motion assumption rather than directly observed. Increasing the modeled sensing frequency provides more detection opportunities along the assumed path, but does not increase the temporal resolution of the GPS data.

This mismatch introduces uncertainty in both trajectory geometry and sensing exposure. Unobserved turns can change the heading and offset assigned to a passage, while pauses and speed variations can change its residence time within a directional footprint. Consequently, interpolation can affect both the binary dwell-time classification and the accumulated probabilistic detection response. The direction of this error is not fixed: the assumed path may either overestimate or underestimate actual sensing exposure.

**Figure 5:**
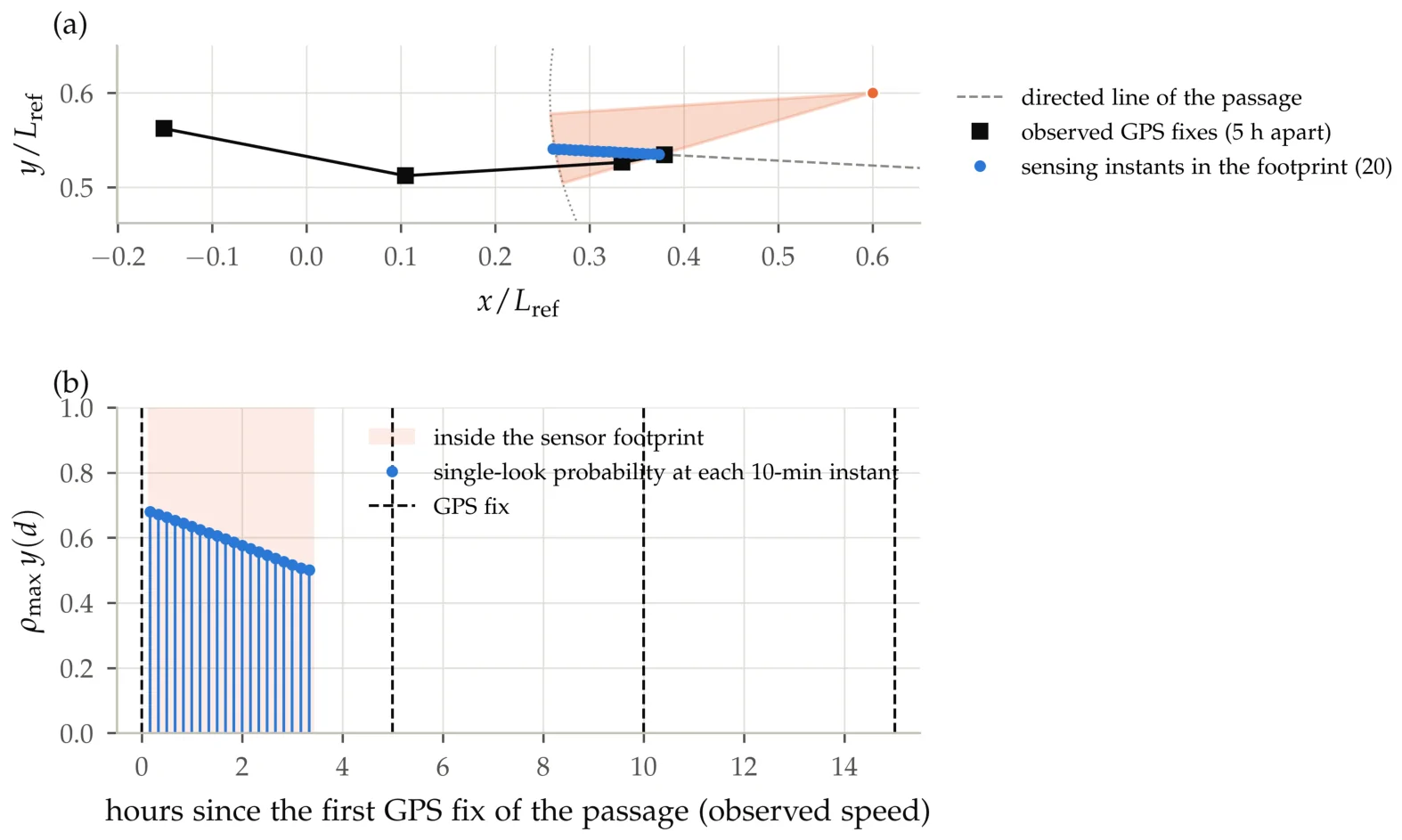
One held-out mule deer passage traced through a deployed sensor. (**a**) Observed GPS fixes, the directed line of the passage, and the sensor footprint and the sensing instants inside it. (**b**) Single-look detection probability at each 10 min sensing instant, on the passage’s own clock (hours since its first GPS fix, at its observed speed). Dashed lines: GPS fixes; shaded: time inside the footprint.

The straightness filter described below restricts the analysis to segments whose observed fixes are approximately collinear, but it cannot exclude deviations between fixes. The wildlife results should therefore be interpreted as deployment comparisons conditional on reconstructed trajectories, rather than validated predictions of detection along the animals’ unobserved paths. On a representative test passage, none of the 20 scheduled sensing instants in the footprint of a deployed sensor falls within 5 min of an observed GPS fix; all of them lie at positions interpolated between the ≈5 h fixes, because the passage enters and leaves the footprint 0.1 h and 3.4 h after its first fix, within a single fix interval. In the reference calculation, the passage is the 4.0 km chord of its directed line through the sensing horizon, traversed at the single modeled speed; its dwell time in the sector, 10.3 h, exceeds $\tau_{\min}$, so it is binary-positive, and its 62 sensing opportunities in the footprint, with single-look probabilities between 0.50 and 0.68, give Γ=1.000 (Figure 5). The scheduled instants in this example illustrate the assumed 10 min sensing interval; they are not the timestamps used by the numerical reference detector, which uses the phase-free approximation described above.

Steps shorter than 60 m were removed, tracks were separated at gaps longer than three fix intervals (15 h), and each trip was divided into approximately linear segments at heading changes exceeding $20^{\circ }$. Segments were retained when they contained at least three fixes and had straightness of at least 0.98. This yields 3524 candidate segments with median length 0.70 km and median straightness 0.997. Of these, 2398 pass through the monitoring disk, that is, the observed segment itself intersects it, and are retained for intensity estimation and deployment. The data are split by individual, with 36 deer assigned to training and 15 to testing and no individual shared between the two sets. The remaining three of the original 54 individuals contribute no retained segments after filtering. The KDE bandwidths, 0.30 rad in heading and 0.40 in offset, were chosen by likelihood cross-validation with folds formed by individuals (Table 4).

**Figure 6:**
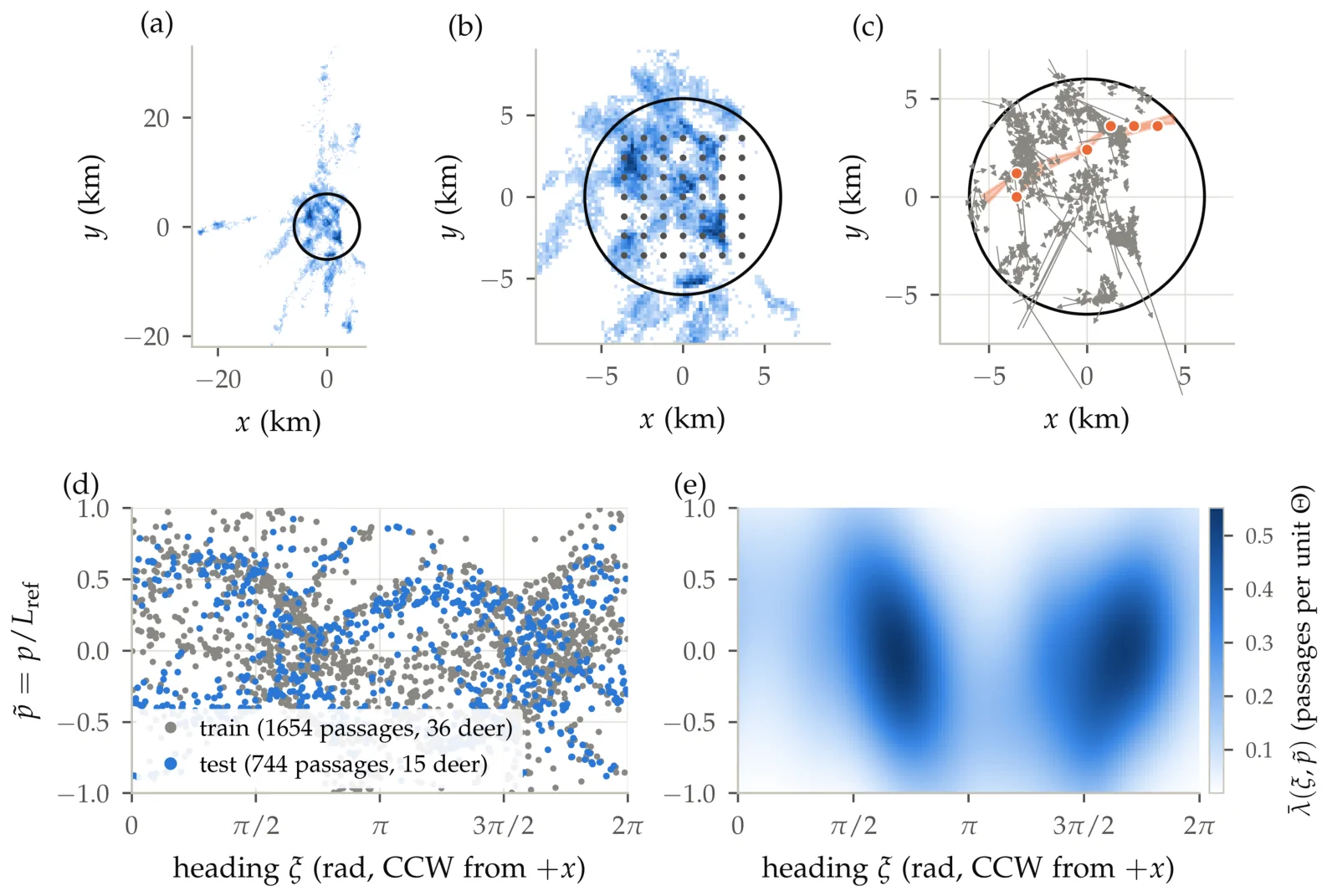
Directional trajectory representation for the mule deer corridor. (**a**) Movement-density heatmap and area of interest. (**b**) Monitoring region Ψ with $L_{\mathrm{ref}}=6$ km. (**c**) Extracted directed linear trajectories. (**d**) Trajectories mapped to $\left(\xi ,\tilde{p}\right)$ and separated into training and testing individuals. (**e**) KDE estimate of $\bar{\lambda }\left(\xi ,\tilde{p}\right)$, showing reciprocal directional modes.

Figure 6 shows that the directed representation exposes a pronounced reciprocal corridor structure. The dominant mode occurs near $125^{\circ }$ and a second mode near $296^{\circ }$. The correlation between $\bar{\lambda }\left(\xi ,\tilde{p}\right)$ and its reciprocal $\bar{\lambda }\left(\xi +\pi ,-\tilde{p}\right)$ is 0.927, indicating repeated travel in opposite directions along similar geometric paths. Thus, as in the maritime case, the directed representation retains information that an undirected line model would merge. The mean normalized offset magnitude is 0.363, and the most represented individual contributes 5.1% of the retained segments.

### 5.4. Binary-Radius Calibration

This subsection evaluates the two-sided binary sensing error characterized by Lemmas 1 and 2. The resulting error curves are then used to examine the distribution-independent minimax rule of Proposition 2 and the deployment-aware criterion of Corollary 3. All radii satisfy the physical constraint in (52).

#### 5.4.1. Maritime

The admissible interval is R=[0.02,0.240]. Consistent with Lemma 1, the binary-positive error $e_{\mathrm{in}}\left(r\right)$ increases from approximately $1.2\times 10^{-14}$ to 0.463 as the binary radius grows. In contrast, the binary-negative bound of Lemma 2 is numerically equal to one throughout the interval. With f=1 Hz, $\tau_{\min}=15$ s, and $\rho_{\max}=0.9$, $N_{<}=\left\lceil f\tau_{\min}\right\rceil =15$ and ${\left(1-\rho_{\max}\right)}^{N_{<}}=10^{-15}$, so the short-dwell term makes the binary-negative bound effectively vacuous. The outer-region count is obtained by enumeration over the evaluated candidate and trajectory sets, counting each disjoint outer interval separately; it decreases from $N_{\max}\left(0.02\right)=405$ to $N_{\max}\left(r^{★}\right)=295$ and to zero at $\tilde{R}$. It therefore applies to the evaluated set, not uniformly; a sweep over all straight lines at the same speed differs by at most one sample. Because ${\left(1-\rho_{\max}\right)}^{N_{<}}=10^{-15}$, neither the corrected $N_{<}$ nor $N_{\max}\left(r\right)$ changes the selected radius.

**Figure 7:**
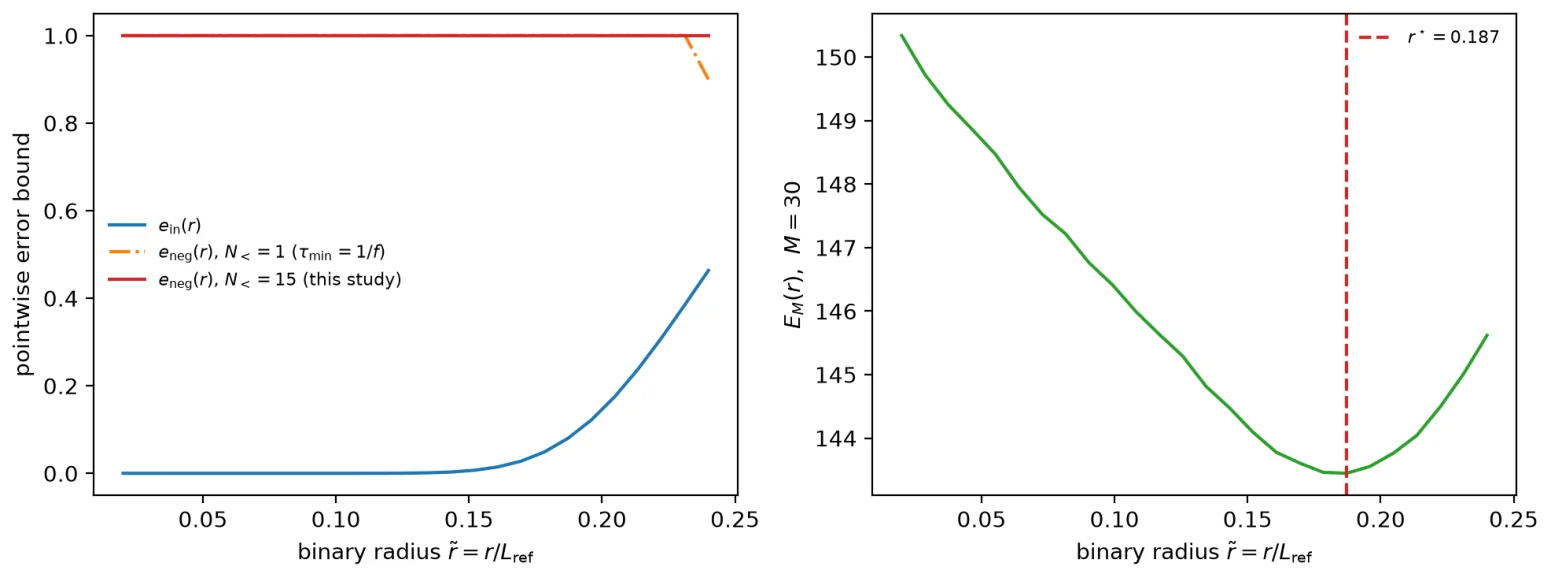
Binary-radius calibration for the Savannah data. **Left**: binary-positive and binary-negative error bounds over $r\leq \tilde{R}=0.240$, together with the one-period ($N_{<}=1$) diagnostic. **Right**: deployment-aware error $E_{M}\left(r\right)$ at M=30, with $r^{★}=0.187$ marked.

Figure 7 illustrates these two behaviors. The left panel shows that $e_{\mathrm{in}}\left(r\right)$ and $e_{\mathrm{neg}}\left(r\right)$ do not intersect; therefore, the condition of Proposition 2 is not satisfied, and the minimax criterion does not discriminate among admissible radii. The one-period curve ($\tau_{\min}=1/f$, hence $N_{<}=1$) from Corollary 1 is shown only as a diagnostic: its hypothesis $\tau_{\min}=1/f$ does not hold for the deployed maritime configuration, and even under that hypothesis, the bound equals $\rho_{\max}=0.9$ at $r=\tilde{R}$ rather than zero.

The deployment-aware criterion is evaluated on a probe set containing all 49 candidate locations with 4 of the 36 orientations ($0^{\circ }$, $90^{\circ }$, $180^{\circ }$, and $270^{\circ }$). The right panel of Figure 7 evaluates the deployment-aware criterion of Corollary 3 instead. It has an interior minimum at

(143) $$\begin{array}{c} r^{★}=0.1872. \end{array}$$

At this radius, $e_{\mathrm{in}}\left(r^{★}\right)=0.0799$, so Lemma 1 guarantees a continuous trajectory-level detection probability of at least 0.9201 for every binary-positive trajectory under the stated assumptions.

The absolute level of $E_{M}\left(r\right)$ remains conservative: it varies by only 2.3% over the admissible interval and exceeds the total trajectory intensity. Consequently, Corollary 3 is more informative here as a radius-ranking criterion than as a tight absolute error certificate.

#### 5.4.2. Wildlife

The modeled sensor is a Pulsar Oryx LRF XG35 thermal imager. Using its detector geometry and a deer-sized target gives an effective sensing horizon of $R_{\mathrm{sens}}=2052$ m, corresponding to $\tilde{R}=0.342$, and a half-FOV angle of $\phi =6.26^{\circ }$. The continuous kernel uses $\rho_{\max}=0.90$ and l=0.199, with revisit frequency f=1/600 Hz. The kernel is $y\left(d;l\right)=\exp\left(-d^{2}/l\right)$ with *d* in units of $L_{\mathrm{ref}}=6$ km, so *ℓ* is expressed in units of $L_{\mathrm{ref}}^{2}$. It is derived from the same criterion as the sensing horizon: $R_{\mathrm{sens}}$ is the Johnson detection range, at which the detection probability is 0.5 [11,12], so *ℓ* is set by $\rho_{\max}y\left(R_{\mathrm{sens}};l\right)=0.5$, that is, $l={\tilde{R}}^{2}/\ln\left(2\rho_{\max}\right)=0.199$ (a distance scale $L_{\mathrm{ref}}\sqrt{l}=2.68$ km). Treating the Johnson probability as the single-look probability is a modeling assumption. Dwell times divide path length by a single speed, $\bar{v}=0.0183$ m/s, the median endpoint speed of the training passages.

The deployed binary model requires $N_{\min}=10$ sensing opportunities, giving $\tau_{\min}=6000$ s and $N_{<}=10$. A one-period setting, $\tau_{\min}=1/f$, is evaluated separately to illustrate Corollary 1.

**Figure 8:**
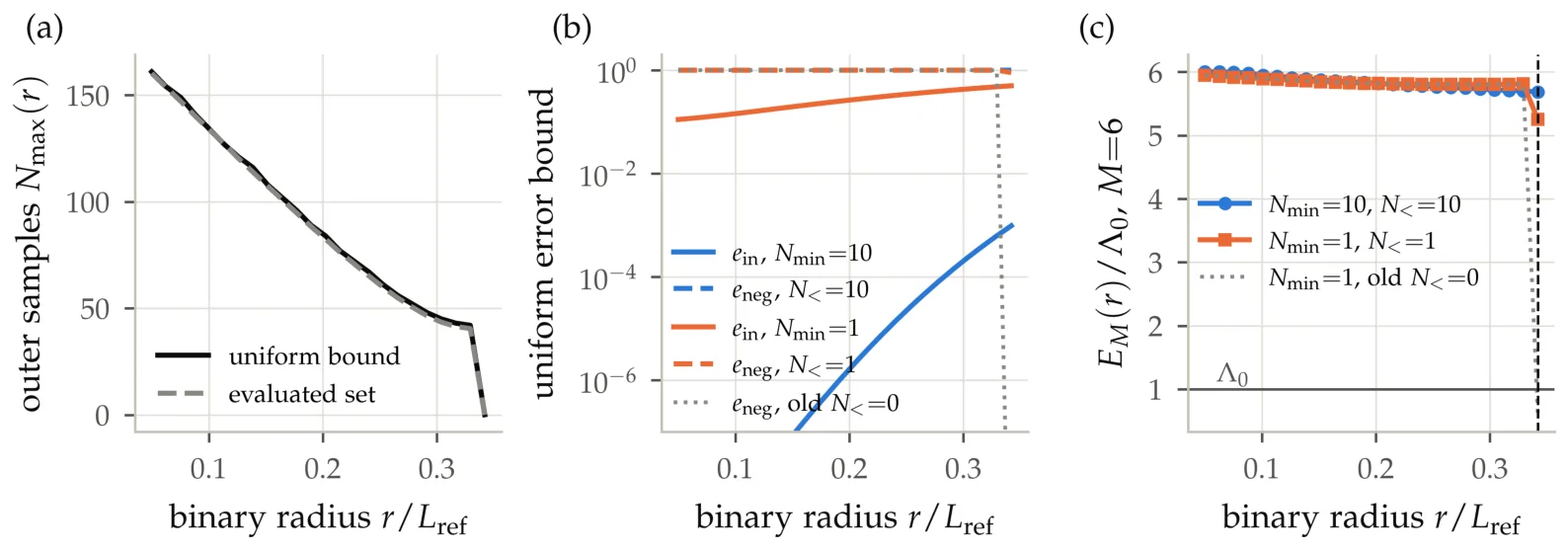
Binary-radius calibration for the mule deer data. (**a**) Geometry-dependent $N_{\max}\left(r\right)$. (**b**) Binary-positive and binary-negative error bounds under the deployed and one-period dwell settings. (**c**) Deployment-aware error $E_{M}\left(r\right)$ at M=6, with $r^{★}=\tilde{R}=0.342$ marked.

Figure 8a evaluates a geometry-specific empirical tightening of the finite-horizon count in Assumption 4 over the candidate and trajectory sets used in this study. The resulting $N_{\max}\left(r\right)$ decreases from 161 at r=0.05 to zero at $r=\tilde{R}$, as expected because no outer sensing annulus remains within the effective sensing horizon. Scheduled samples are counted separately in each disjoint outer interval, so the count applies to the evaluated set, not uniformly; a sweep over all straight passages at $\bar{v}$ differs from it by at most 2 samples.

Figure 8b evaluates the two trajectory-level lemmas. Under the deployed dwell requirement, $e_{\mathrm{in}}\left(r\right)$ remains extremely small, from $2.9\times 10^{-10}$ to $9.8\times 10^{-4}$, whereas $e_{\mathrm{neg}}\left(r\right)\approx 1$ because ${\left(1-\rho_{\max}\right)}^{N_{<}}=10^{-10}$. The deployed configuration therefore lies in a regime where $e_{\mathrm{in}}\left(r\right)$ is very small, whereas the uniform binary-negative bound is uninformative.

The one-period diagnostic behaves differently. With $\tau_{\min}=1/f$, $N_{<}=1$, so Corollary 1 retains one short-dwell factor: $e_{\mathrm{in}}\left(r\right)$ increases from 0.111 to 0.500, while $e_{\mathrm{neg}}\left(r\right)=1-\left(1-\rho_{\max}\right){\left(1-p_{r}\right)}^{N_{\max}\left(r\right)}$ decreases from approximately 1 to $\rho_{\max}=0.9$ at $\tilde{R}$, not to zero. Because $e_{\mathrm{neg}}\left(r\right)\geq 0.9>e_{\mathrm{in}}\left(r\right)$ throughout, the two bounds do not cross, and the minimax objective in (84) is not informative in this diagnostic setting either.

Finally, Figure 8c evaluates Corollary 3. The deployment-aware criterion selects

(144) $$\begin{array}{c} r^{★}=\tilde{R}=0.342. \end{array}$$

At $r^{★}$, $e_{\mathrm{in}}\left(r^{★}\right)=$$9.8\times 10^{-4}$, corresponding through Lemma 1 to a continuous detection lower bound of approximately 0.999 for every binary-positive trajectory under the stated model assumptions.

Under the deployed setting, $E_{M}\left(r\right)$ changes by only 5.2% and remains larger than the attainable objective range, again showing that its level is conservative. Nevertheless, the same radius independently minimizes the measured continuous–binary objective error, which reaches 0.147 expected passages at $r^{★}$. This agreement supports the selected radius even though the theoretical envelope itself is loose.

Across both datasets, the uniform binary-negative bound is therefore the main source of conservativeness under the deployed dwell requirements, whereas the deployment-aware criterion remains usable for selecting a binary radius within the effective sensing horizon.

### 5.5. Continuous-Versus-Binary Approximation

We next evaluate how the trajectory-level errors propagate to the deployment objective and greedy decisions. Theorem 1 provides the network-level objective bound, while Lemma 3 and Corollary 4 characterize candidate-level and one-step greedy distortion. The exact-decision conditions of Corollaries 5 and 6 are evaluated where the required diagnostics are available.

#### 5.5.1. Maritime

**Figure 9:**
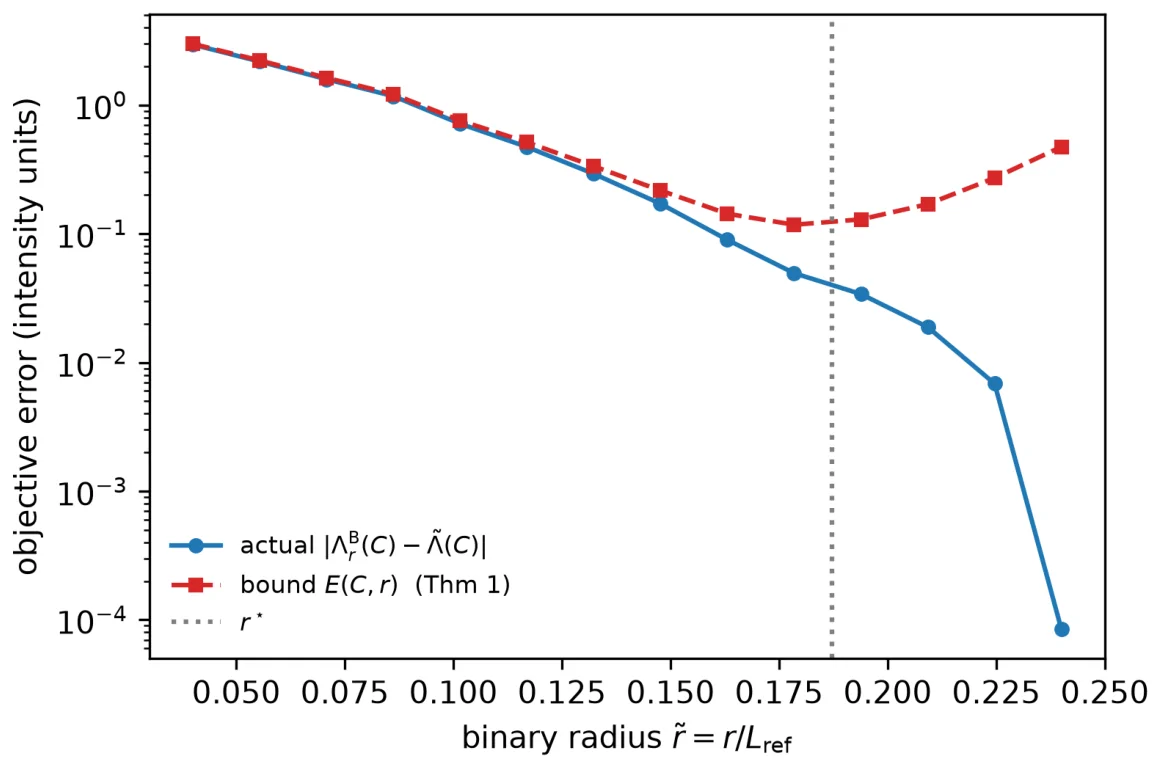
Measured continuous–binary objective error and theoretical network bound for the Savannah data.

Figure 9 compares the measured objective error $|\Lambda_{r}^{B}\left(C\right)-\tilde{\Lambda }\left(C\right)|$ with the bound E(C,r) from Theorem 1 for the M=30 deployment. The measured error decreases from 2.948 at r=0.040 to $8\times 10^{-5}$ at the sensing horizon. Over the lower half of the admissible range, the bound exceeds the measured error by only approximately 1–15%; at $r^{★}$, it is 0.129 against a measured error of 0.034. Thus, unlike the single-sensor deployment-aware envelope $E_{M}\left(r\right)$, the sharp network envelope can become informative once a sufficiently large deployment covers much of the trajectory domain.

The additive network bound in (82) is substantially looser because it discards the binary-positive/binary-negative structure. The comparison therefore shows that retaining the binary-positive/binary-negative structure yields a more informative network-level bound on this deployment.

**Figure 10:**
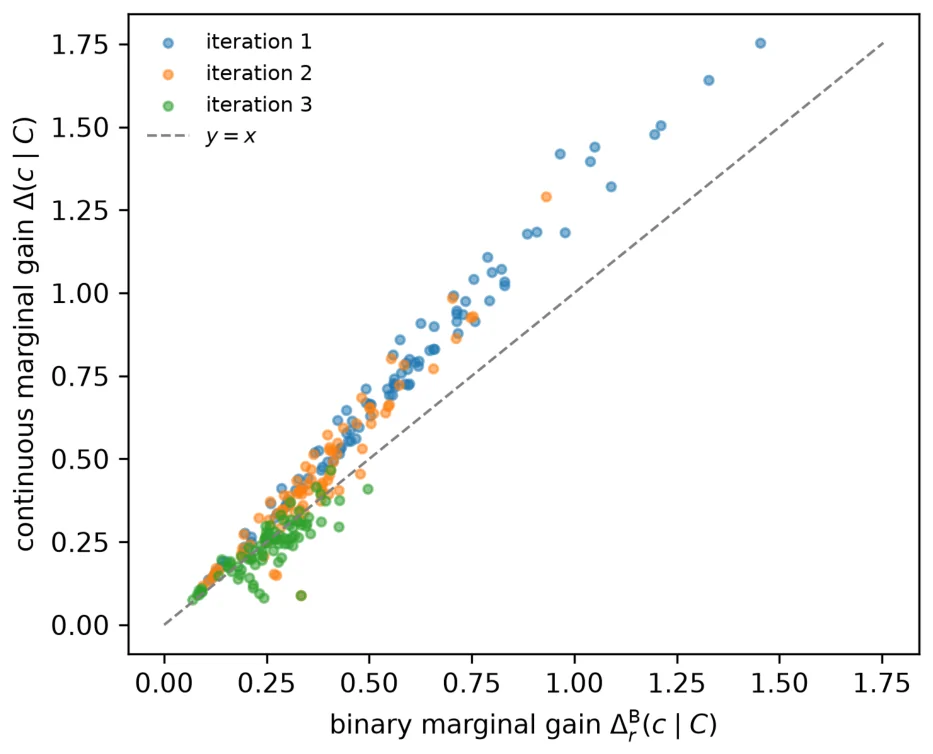
Continuous and binary marginal gains over three representative greedy iterations for the Savannah data.

**Figure 11:**
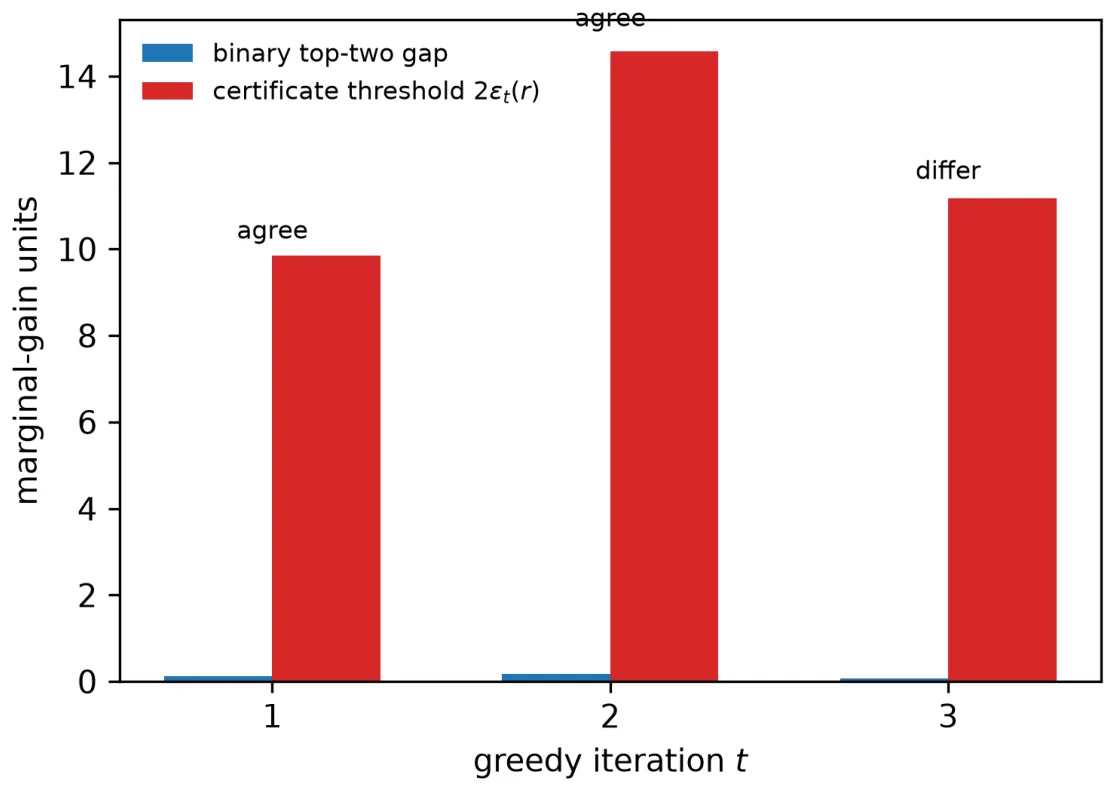
Binary–continuous selection agreement and exact decision certificate, Savannah data.

Figure 10 and Figure 11 examine the more relevant question for greedy deployment: whether the binary approximation preserves candidate ranking. On a reduced instance of 16 locations and 6 orientations, the Spearman correlations between binary and continuous marginal gains are 0.991, 0.944, and 0.829 over the first three iterations. The argmax agrees in the first two iterations and differs in the third.

Figure 10 shows that even where the rankings diverge, the continuous marginal-gain penalty is small. The observed losses associated with the binary-selected candidate are 0, 0, and 0.055 intensity units, with the last corresponding to approximately 1% of the total trajectory intensity. This directly evaluates Corollary 4: the observed loss is much smaller than the allowed $2\varepsilon_{t}\left(r\right)$.

Figure 11 evaluates the simpler exact-decision certificate of Corollary 6. It does not fire at any of the three iterations even though the binary and continuous decisions coincide twice. The reason is that $\varepsilon_{t}\left(r\right)$ depends on E(C,r) at small partial deployments, where many trajectories remain in the all-negative branch and $e_{\mathrm{neg}}\left(r\right)\approx 1$. Thus, the certificate remains a valid sufficient condition but is conservative in this regime. The candidate-specific certificate of Corollary 5 is not used as the primary maritime diagnostic because it requires separate candidate-wise error intervals; it is examined explicitly in the wildlife study below.

Corollary 2 follows from the same missed-intensity error through an exponential transformation. A separate error plot is therefore not required; the corresponding void probabilities are reported directly with the final deployment results in Section 5.7.

#### 5.5.2. Wildlife

The measured continuous–binary objective error decreases from 1.40 at the smallest admissible radius to 0.147 at $r^{★}=\tilde{R}$. The measured error remains below the bound of Theorem 1 throughout the sweep, but the bound is substantially more conservative under the deployed dwell setting because $e_{\mathrm{neg}}\left(r\right)\approx 1$.

**Figure 12:**
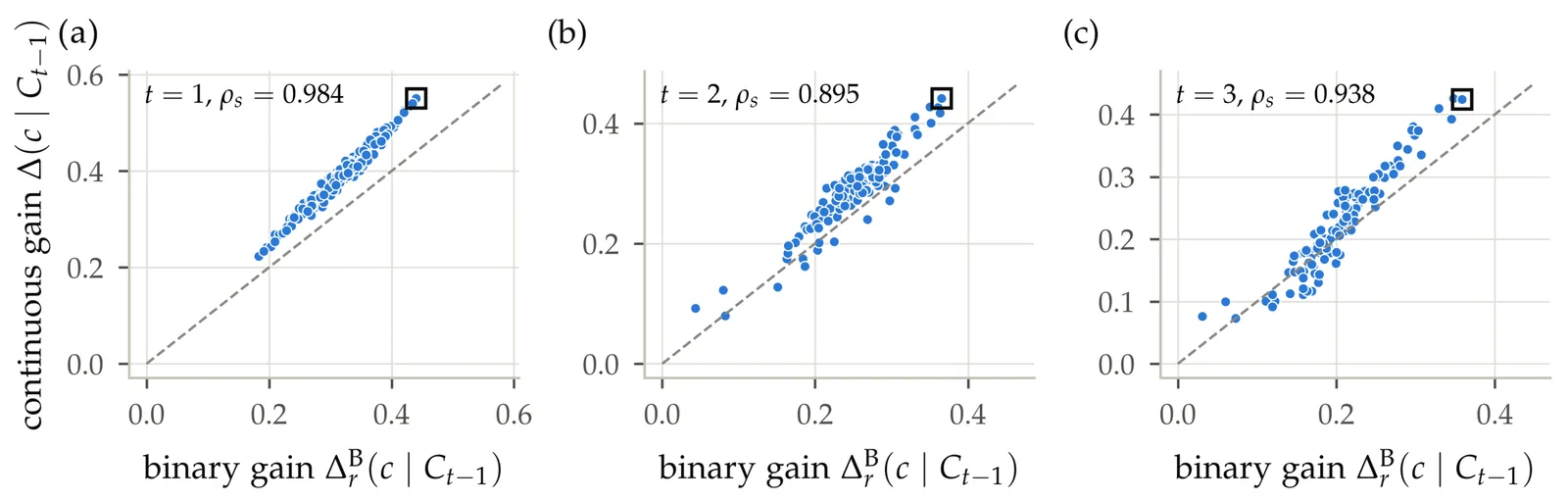
Continuous and binary marginal gains over three representative greedy iterations for the mule deer data.

Figure 12 shows that candidate ranking is substantially more accurate than the absolute objective bound suggests. On a reduced instance of 9 locations and 18 orientations, the Spearman correlations are 0.984, 0.895, and 0.938 over three iterations under the deployed dwell setting. Four of the top five candidates coincide at every iteration; the binary and continuous argmax agree at iterations 1 and 2 and differ at iteration 3, where the observed one-step loss of Corollary 4 is $1.6\times 10^{-3}$. The disagreement comes from passages that dwell less than $\tau_{\min}$ in a sector: the binary model scores them zero, whereas the reference detector, with many looks at single-look probability of at least 0.50, detects them with high probability. Under the one-period setting, the correlations are 0.999, 0.997, and 0.999; the top-five sets coincide at every iteration, and the binary and continuous argmax agree at every iteration.

**Figure 13:**
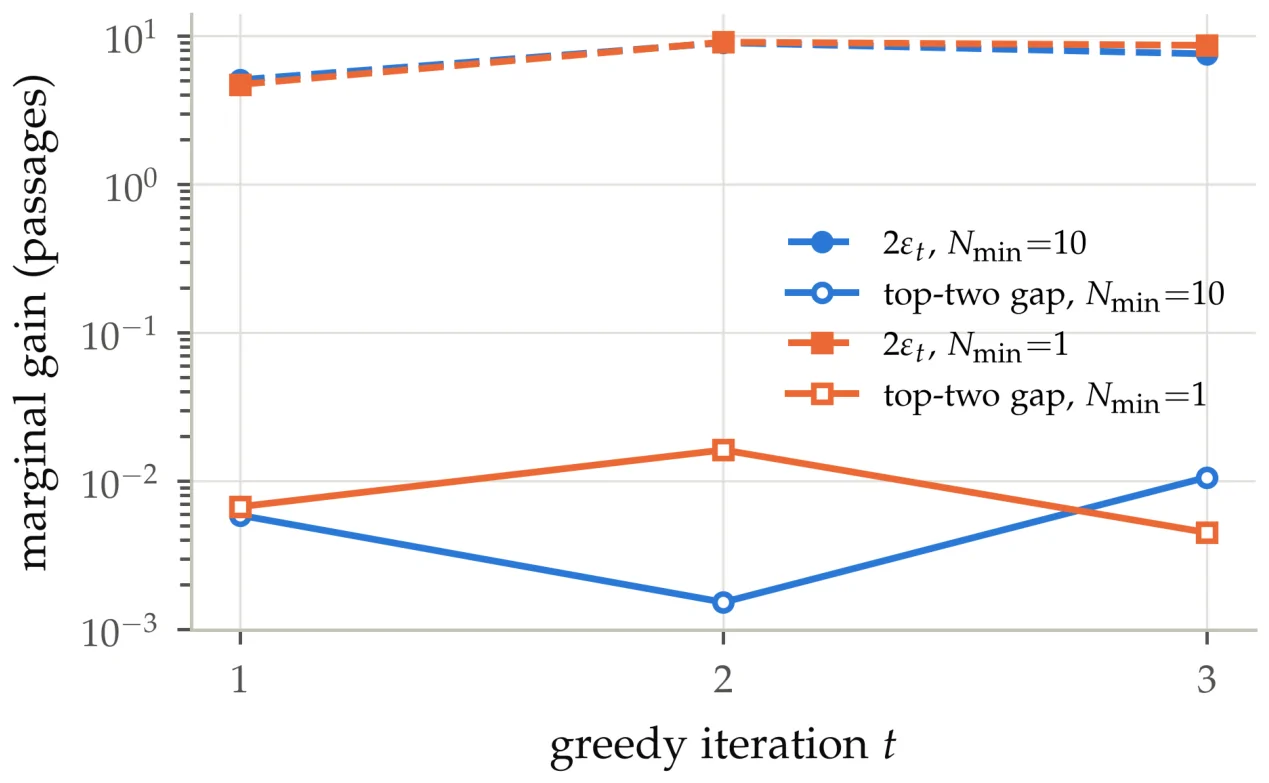
Binary–continuous selection agreement and decision certificates for the mule deer data under both dwell settings.

Figure 13 compares this observed agreement with both the uniform certificate of Corollary 6 and the candidate-specific certificate of Corollary 5. Neither fires under either dwell setting, although the argmax agrees at two of the three iterations under the deployed setting and at all three under the one-period setting. Under the deployed dwell setting, the dominant source of conservativeness is the binary-negative branch of E(C,r). Under the one-period diagnostic, $e_{\mathrm{neg}}$ is smaller, but the integrated candidate-specific intervals remain wider than the observed top-two marginal-gain separations. The result therefore illustrates an important distinction: failure of a sufficient certificate does not imply failure of the approximation.

Across the six representative greedy iterations examined over the two datasets, the binary and continuous argmax agree in four. In the two disagreements, the continuous marginal-gain loss is 0.055 (maritime) and $1.6\times 10^{-3}$ (wildlife). The sufficient decision certificates are therefore considerably more conservative than the observed ranking behavior.

### 5.6. Omni-Greedy Approximation

We next evaluate the second approximation identified in Section 4: replacing joint location–orientation ranking with an omnidirectional location proxy. Theorem 2 bounds the discrepancy through $A_{r}+B_{r}$, Corollary 7 converts that discrepancy into a one-step location loss, and Corollary 8 provides a sufficient condition for exact location agreement.

#### 5.6.1. Maritime

**Figure 14:**
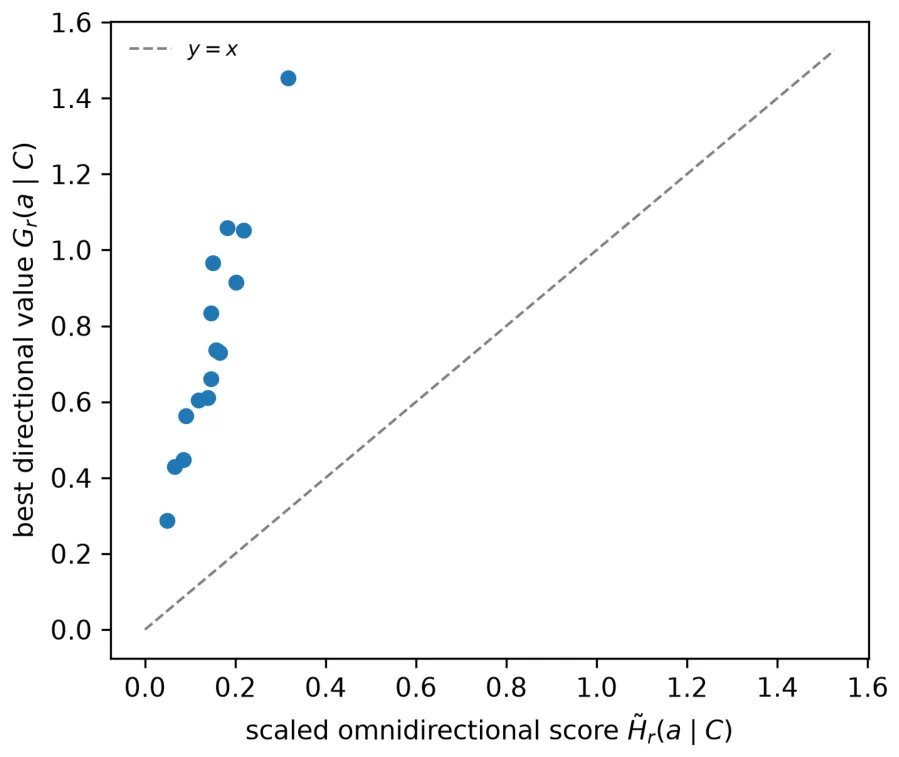
Scaled omnidirectional location score versus best directional location value for the Savannah data.

**Figure 15:**
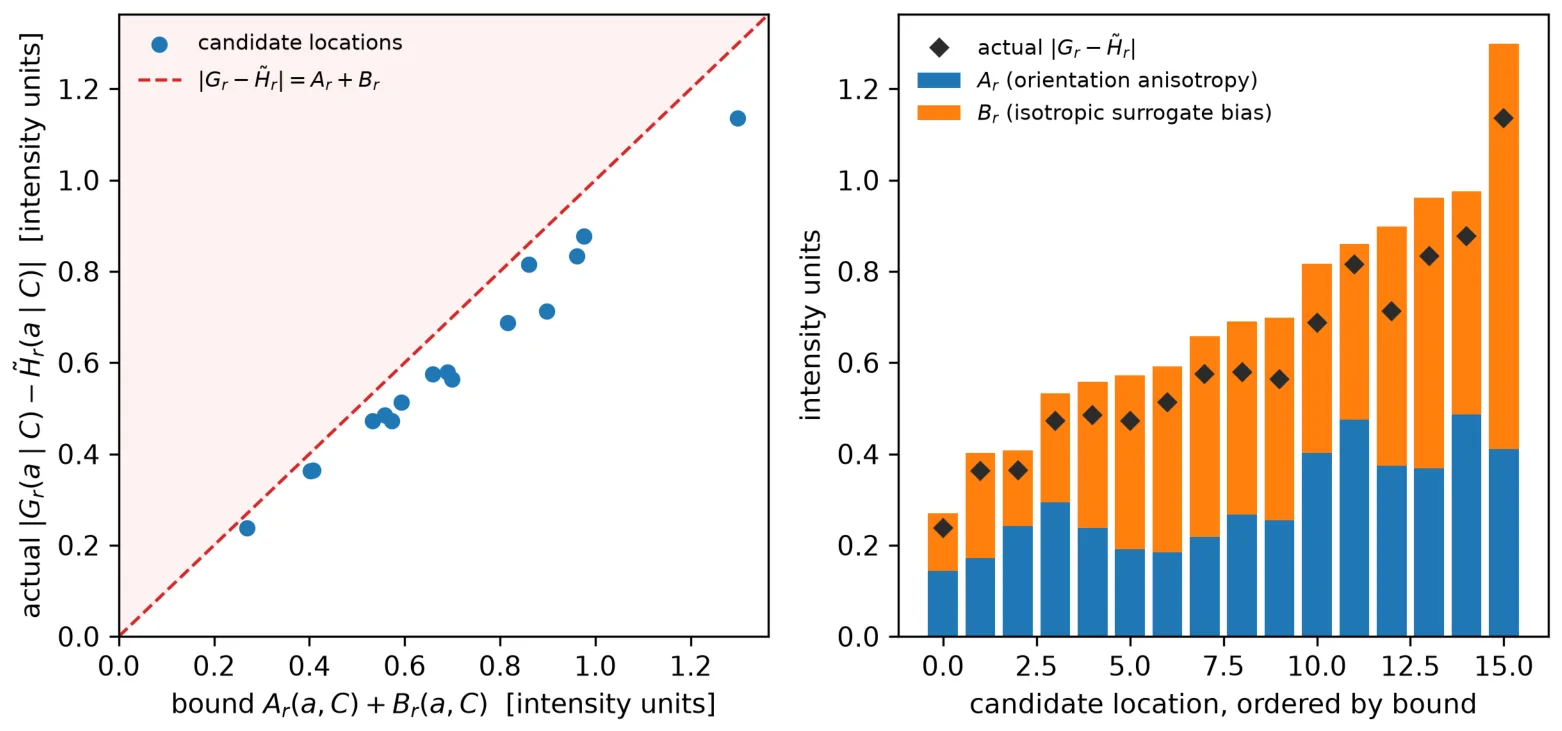
Omnidirectional-to-directional discrepancy and its decomposition into $A_{r}$ and $B_{r}$, Savannah data.

Figure 14 and Figure 15 compare the scaled omnidirectional score ${\tilde{H}}_{r}\left(a|C\right)$ with the best directional value $G_{r}\left(a|C\right)$ for K=36 uniformly spaced orientations.

Figure 15 explains the discrepancy quantitatively. The bound from Theorem 2 is satisfied at every evaluated location, and the median ratio of the observed discrepancy to $A_{r}\left(a,C\right)+B_{r}\left(a,C\right)$ is 0.872.

The decomposition in Figure 15 is also informative. The median fraction $B_{r}/\left(A_{r}+B_{r}\right)$ is 0.58, showing that the isotropic-surrogate discrepancy is a substantial component of the total location-level bound. Using $A_{r}$ alone fails at every evaluated location. Thus, the omnidirectional approximation error is not explained solely by sensitivity to orientation; the mapping from total omnidirectional exposure to average directional exposure is itself a substantial source of discrepancy.

Along six representative greedy iterations, omni-greedy selects the same location as full binary directional greedy in four iterations. At the two disagreements, directional greedy’s selected location is ranked second and third by the omnidirectional proxy. The corresponding observed losses in best-directional value are 0.175 and 0.025 intensity units, while the remaining four losses are zero. These values satisfy the $2\zeta_{t}\left(r\right)$ bound of Corollary 7 with substantial margin.

The exact location condition of Corollary 8 does not certify the two disagreement cases. In these cases, the change in selected location produces only a small one-step objective loss, consistent with several candidate locations having similar residual trajectory value.

#### 5.6.2. Wildlife

**Figure 16:**
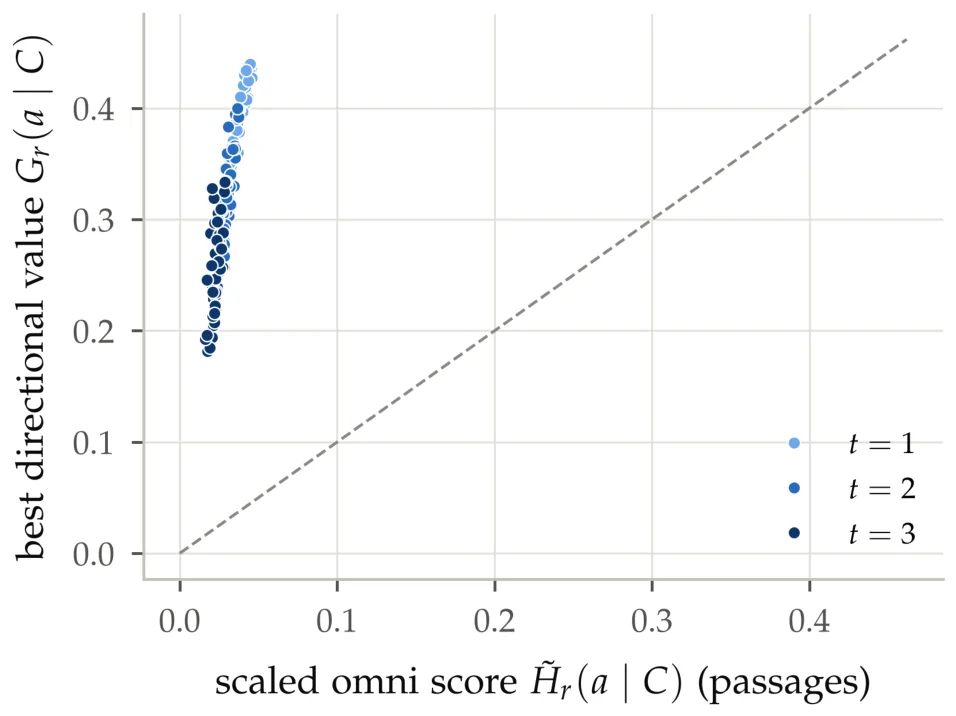
Scaled omnidirectional location score versus best directional value for the mule deer data at the first three omni-greedy iterations (deployed dwell setting).

Figure 16 again shows a systematic relationship between the scaled omnidirectional score and the best directional value, together with location-dependent deviations that can alter the selected site.

**Figure 17:**
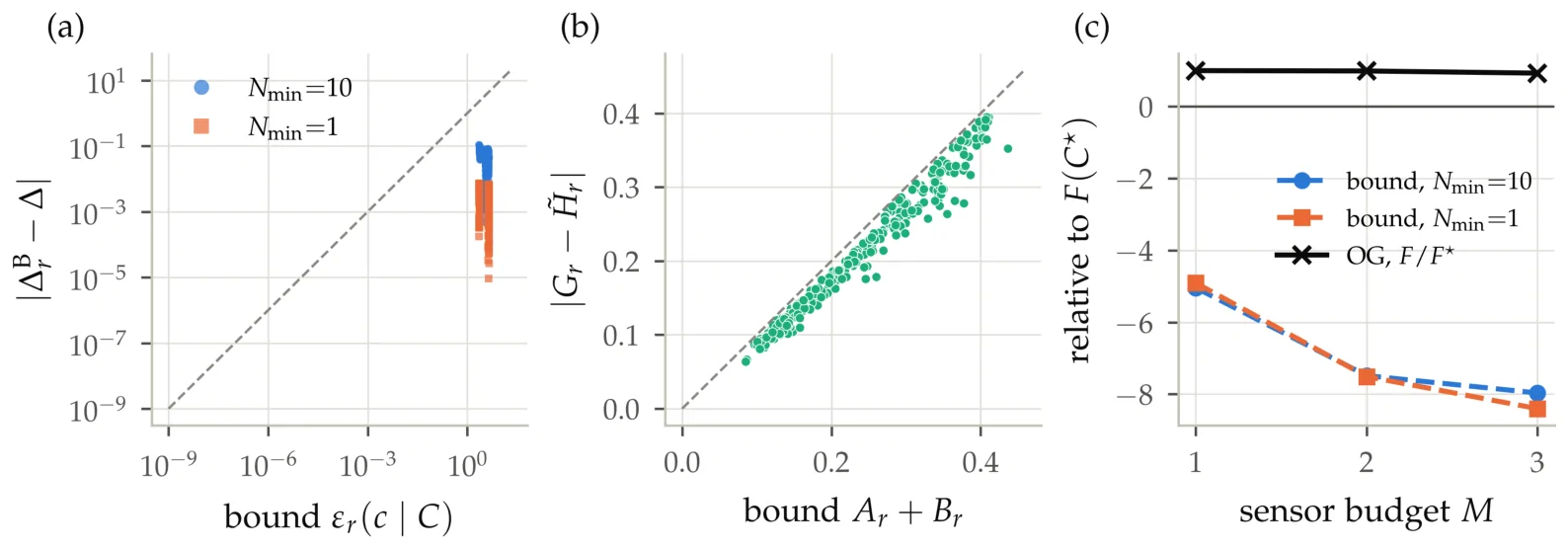
Observed approximation errors and theoretical bounds for the mule deer data. (**a**) Binary marginal-gain distortion. (**b**) Omni-to-directional discrepancy. (**c**) End-to-end guarantee on the enumerable instance.

Figure 17b directly evaluates Theorem 2. The bound holds at every evaluated location–iteration pair along the first six omni-greedy iterations, with a median observed-error-to-bound ratio of 0.86–0.94. As in the maritime case, the median fraction $B_{r}/\left(A_{r}+B_{r}\right)$ is 0.59–0.71. The similar decomposition across the two datasets indicates that both directional anisotropy and isotropic-surrogate mismatch contribute materially to the location-level discrepancy.

At each of its first six partial deployments, omni-greedy selects the best directional location in two of six iterations. At the remaining iterations, the observed loss in best-directional value is 0.007–0.037 intensity units, compared with $2\zeta_{t}\left(r\right)$ bounds of 0.50–0.87. Thus, the observed losses remain below the $2\zeta_{t}\left(r\right)$ bound of Corollary 7, but with substantial slack. The exact certificate of Corollary 8 does not fire.

Figure 17a additionally places the binary candidate-level distortion of Lemma 3 beside its bound, while panel (b) does the same for the omni-greedy approximation. Together, the first two panels show that the binary bounds are substantially more conservative than the $A_{r}+B_{r}$ location bound.

Taken together, the two studies show that the observed location-level errors remain below the $A_{r}+B_{r}$ envelope. Moreover, the disagreement cases observed here incur relatively small one-step objective losses even when the selected locations differ.

### 5.7. Deployment Performance

The previous subsections evaluate the two approximations separately. Theorem 3 combines them at a single greedy iteration, while Theorem 4 propagates their effects across the full sensor budget. Proposition 1 provides the structural greedy baseline, and Corollary 9 translates the final benefit guarantee back to the deterministic void-probability metric.

Rather than introducing a separate experiment for Theorem 3, its two terms have already been measured individually above: $\varepsilon_{t}\left(r\right)$ through the continuous–binary comparisons and $\zeta_{t}\left(r\right)$ through the omni-greedy comparisons. We now evaluate their cumulative deployment consequences.

#### 5.7.1. Performance Normalization

For a fixed evaluation intensity $\bar{\lambda }$, the normalization denominator is the total expected passage count

$$\Lambda_{0}=\int_{\Theta }\bar{\lambda }\left(x\right)\;dx>0.$$

The normalized detected intensities under the probabilistic reference model and binary surrogate are, respectively,

(145) $$D_{C}\left(C\right)=\frac{F(C)}{\Lambda_{0}}=1-\frac{\tilde{\Lambda }\left(C\right)}{\Lambda_{0}},\;D_{B}\left(C\right)=\frac{F_{r}^{B}\left(C\right)}{\Lambda_{0}}=1-\frac{\Lambda_{r}^{B}\left(C\right)}{\Lambda_{0}}.$$

Both quantities lie in [0,1] and represent the fraction of the modeled trajectory intensity detected by the deployment. All methods evaluated against the same intensity use the same denominator. This normalization removes dependence on a common multiplicative scaling of $\bar{\lambda }$; it does not normalize performance relative to an optimal deployment.

In the quadrature implementation, the corresponding denominator is $\sum_{j=1}^{J}w_{j}$. Thus,

$$D_{C}\left(C\right)\approx \frac{\sum_{j=1}^{J}w_{j}\left[1-\Pi \left(x_{j};C\right)\right]}{\sum_{j=1}^{J}w_{j}},$$

with the analogous expression using $\Pi_{r}^{B}$ for $D_{B}\left(C\right)$. In the experiments, $\bar{\lambda }$ is a periodic kernel density estimate of the training segments, each weighted in proportion to its length with the weights normalized to sum to the number of segments. The estimate is then rescaled so that $\Lambda_{0}$ equals the expected number of training segments in the observation window; for the maritime data, 144 training segments over a 688 h span give $\Lambda_{0}=0.2093\;h^{-1}\times 24\;h=5.02$. A passage in this count is an extracted linear segment: curved transits contribute on average 1.85 segments. Length weighting changes the shape of $\bar{\lambda }$ but not $\Lambda_{0}$. The ratios $D_{C}$ and $D_{B}$ do not depend on this scale, whereas $\tilde{\nu }\left(C\right)=\exp\left[-\Lambda_{0}\left(1-D_{C}\left(C\right)\right)\right]$ does.

#### 5.7.2. Maritime

**Figure 18:**
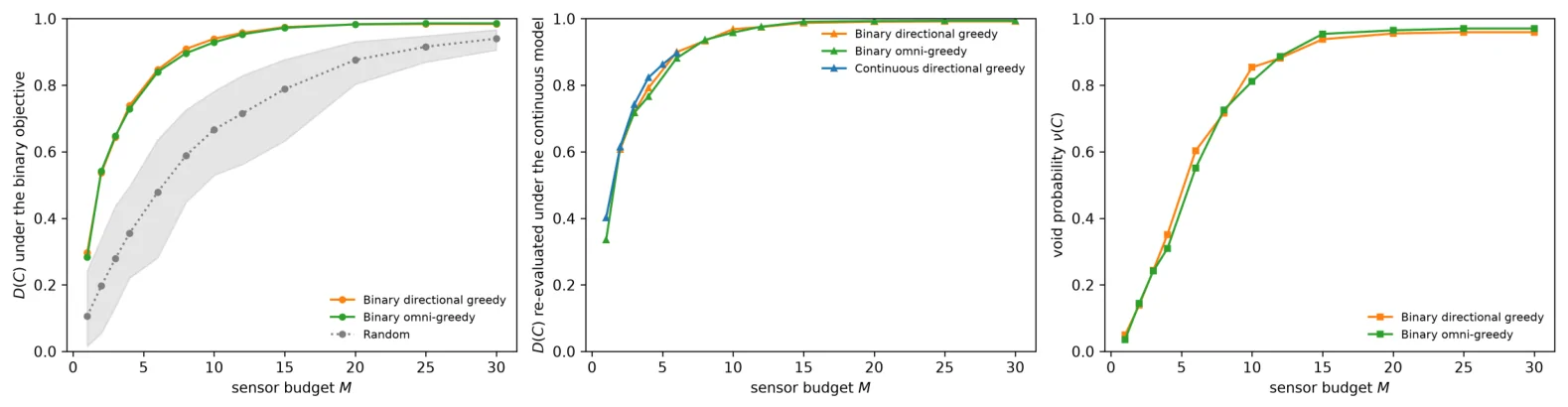
Detection performance versus sensor budget for the Savannah data. **Left**: normalized detected intensity under the binary objective. **Center**: the same deployments under the continuous objective. **Right**: void probability over a 24 h observation window.

The full candidate set contains 49 locations and 36 orientations. Figure 18 and Table 5 compare binary directional greedy (DG), binary omni-greedy (OG), continuous directional greedy (CDG), and random deployment. CDG is evaluated through M=6 in this comparison; with cached candidate responses (Section 5.8), it extends to M=30 at a total cost of 41 s, reaching $D_{C}=0.9895$, compared with 0.9918 for DG and 0.9941 for OG.

The left panel of Figure 18 shows performance under the binary objective, whereas the center panel re-evaluates the same deployments under the continuous reference model. This separation is important because it reveals whether the binary surrogate merely favors its own deployment or also produces a strong solution under the original sensing model. All structured methods substantially outperform random placement.

Under the continuous model, DG and CDG remain close: CDG exceeds DG by 0.008 at M=2 and 0.031 at M=4, whereas DG is higher by 0.003 at M=6. These close values do not reflect identical deployments: DG and CDG select the same first sensor, but their configurations differ from M=2 onward, and at M=6, they share 3 of 6 location–orientation pairs and 4 of 6 locations (Table 6). All scores are computed by one reference-detector evaluation with the same trajectories, weights, speed rule, and normalization, so identical configurations receive identical scores, as at M=1. Thus, replacing continuous sensing with the calibrated binary surrogate has little effect on final deployment quality over the range where CDG is computationally available. OG also remains close to DG and becomes marginally higher at several larger budgets, consistent with several near-equivalent greedy paths.

The right panel of Figure 18 reports the corresponding void probability over a 24 h observation window. This provides the operational interpretation associated with Corollaries 2 and 9: as missed trajectory intensity decreases, the probability that no trajectory is missed increases nonlinearly. Because the observation window is fixed, this transformation does not alter the within-dataset deployment ranking.

Table 5 provides the numerical values underlying Figure 18 and shows that the performance differences among the informed methods become small as the budget increases.

The end-to-end guarantee is examined on a reduced enumerable instance, where directional greedy attains the enumerated binary optimum and is within 1.7% of the enumerated continuous optimum. Binary omni-greedy attains the enumerated continuous optimum on the same instance. In contrast, the lower bound from Theorem 4 is negative because $\sum_{t}\varepsilon_{t}\left(r\right)$ is much larger than $\sum_{t}\zeta_{t}\left(r\right)$. The bound is therefore numerically vacuous on this instance. Its decomposition nevertheless identifies the binary sensing term as the dominant source of theoretical slack.

**Figure 19:**
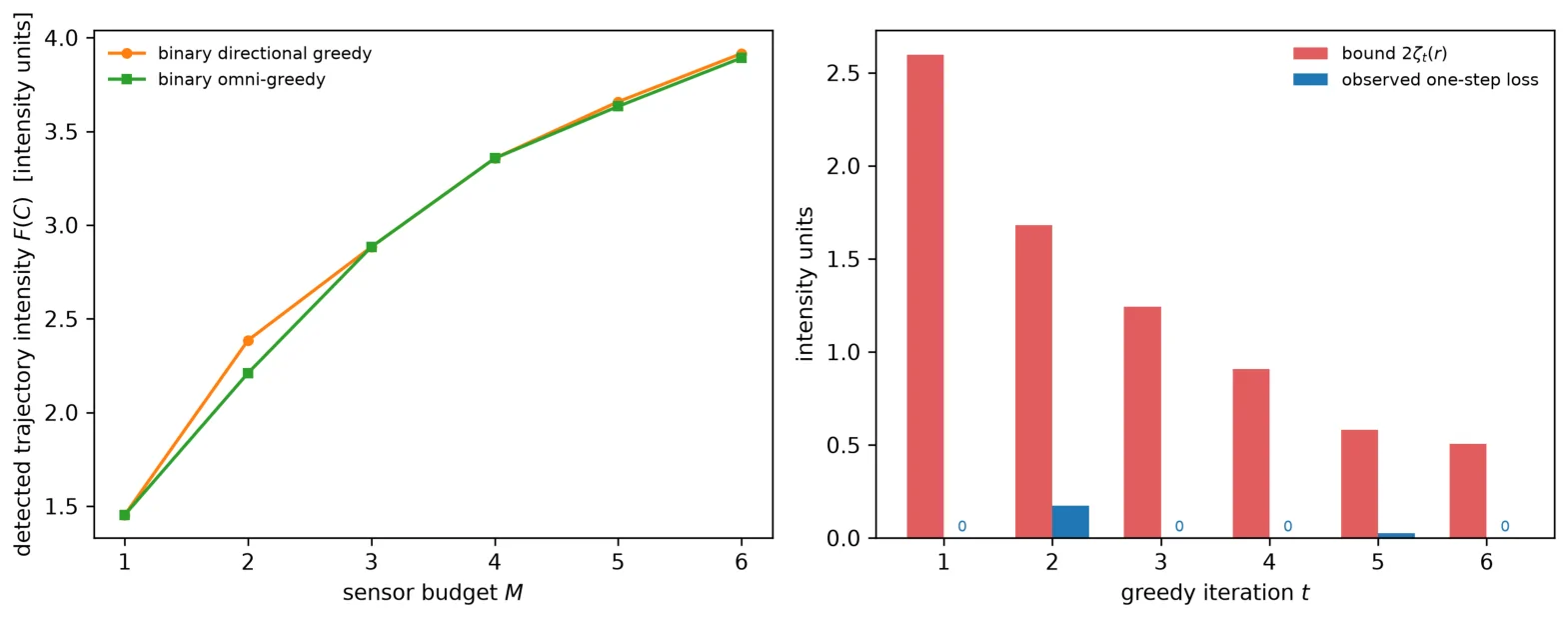
Location–orientation decoupling on the reduced Savannah instance (L=16, K=36, M≤6). **Left**: detected intensity for DG and OG, an empirical comparison; no bound on their difference is implied. **Right**: at each omni-greedy partial deployment $S_{t}^{\mathrm{OG}}$, the one-step loss (best feasible binary directional marginal gain minus the gain of the omni-greedy choice) and $2\zeta_{t}\left(r\right)$ evaluated at the same partial deployment, in the same intensity units.

Figure 19 isolates the second term. The left panel shows that DG and OG attain closely matched deployment values, while the right panel compares the observed one-step location loss directly with the $2\zeta_{t}\left(r\right)$ allowance of Corollary 7. The bound holds at every tested step but is larger than the observed loss, confirming that the decoupling contribution is controlled but conservative. The one-step loss is zero at four of six steps and at most 0.175 intensity units, against allowances between 0.50 and 2.60. The final DG–OG differences in the left panel are empirical comparisons only: because the two algorithms follow different selection sequences, the one-step corollary does not bound their cumulative difference.

Because $S_{M}\left(r\right)$ is large on the reduced instance, the corresponding void-probability lower bound of Corollary 9 is also extremely conservative. This conservativeness does not reflect the much smaller empirical performance gap observed on the reduced enumerable instance.

#### 5.7.3. Wildlife

**Figure 20:**
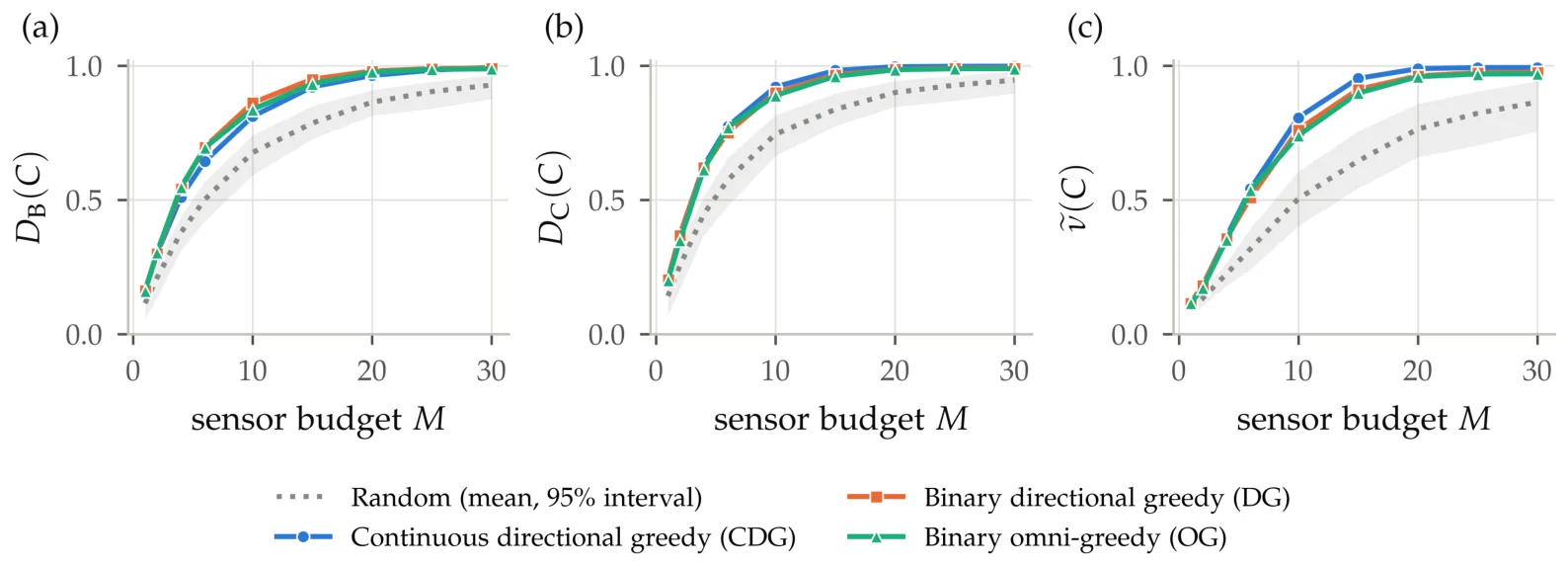
Detection performance versus sensor budget for the mule deer data. (**a**): normalized detected intensity under the binary objective. (**b**): continuous evaluation of the same deployments. (**c**): void probability using T=2.71 expected passages over the specified 14-day monitoring window.

The full wildlife candidate set also contains 49 locations and 36 orientations. Figure 20 and Table 7 provide the same comparison as in the maritime case.

The saved selection sequences show that binary and continuous directional greedy select identical configurations, in the same order, only at M=1, where their scores therefore match exactly. From the second sensor on, they diverge: at M=6, they share 1 of 6 configurations and 3 of 6 locations, and their normalized detected intensities under the reference model are 0.7505 and 0.7733, respectively. Similar objective values do not imply identical deployments.

Under the binary objective, OG is within 0.025 of DG at every budget. Under the reference model, OG is ahead of DG at M=6 (0.7697 versus 0.7505), and at M=20 the values are 0.9856 for DG and 0.9843 for OG. The result reinforces the distinction established in Section 5.6: location decisions can differ substantially while final objective values remain close.

The right panel of Figure 20 reports void probability using T=2.71 expected passages over the specified 14-day monitoring window. *T* counts retained passages (linear segments crossing the monitoring disk), not whole transits: 1654 training passages over 8541 individual days of tracking give 0.194 passages per individual day, times 14 days. The length-weighted KDE is rescaled so that $\Lambda_{0}=\int_{\Theta }\bar{\lambda }\;dx=T$. The normalized detected intensity *D* does not depend on this scale, whereas $\tilde{\nu }=\exp\left[-T\left(1-D\right)\right]$ does. As in the maritime study, this supplies the probability interpretation of the missed-intensity objective while preserving the within-dataset ranking.

Table 7 shows that the DG–OG difference is small at every budget but does not decrease monotonically with *M*.

Exhaustive enumeration on the 9-location, 4-orientation instance shows that directional greedy attains the binary optimum at M=1 and 3 but not at M=2, where it is within 0.1%; continuous directional greedy attains the continuous optimum at M=1 and 2. Binary omni-greedy attains 0.999, 0.989 and 0.929 of the continuous optimum at M=1,2,3.

Figure 17c compares this observed result directly with Theorem 4. On this reduced enumerable instance, the empirical deployment attains or nearly attains the enumerated optimum, whereas the theoretical lower bound is negative at the tested budgets (between −8.0 and −5.0 times the optimum). Under the deployed dwell setting, $\sum_{t}\varepsilon_{t}\left(r\right)$ exceeds $\sum_{t}\zeta_{t}\left(r\right)$ by a factor of 9.8 at M=3. Thus, as in the maritime case, the binary sensing term dominates the theoretical slack in the end-to-end bound.

**Figure 21:**
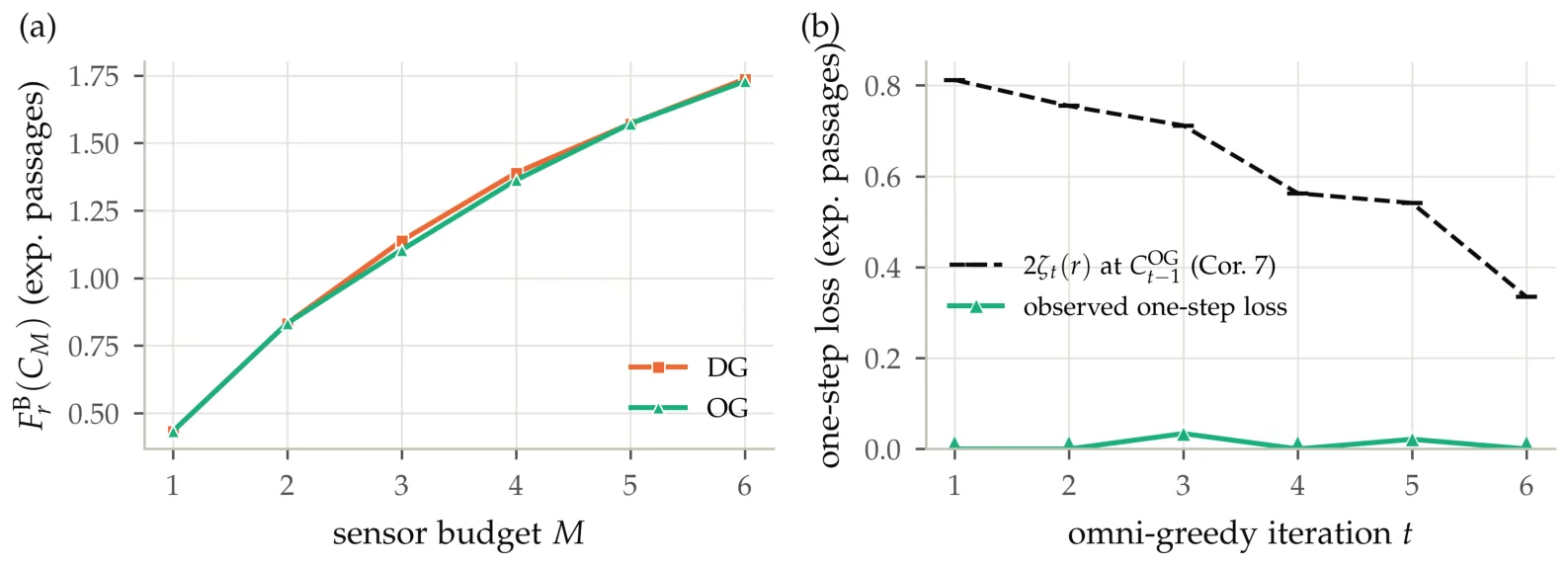
Location–orientation decoupling on the reduced mule deer instance (L=16, K=36, M≤6). (**a**): detected intensity for DG and OG, an empirical comparison; no bound on their difference is implied. (**b**): at each omni-greedy partial deployment $S_{t}^{\mathrm{OG}}$, the one-step loss (best feasible binary directional marginal gain minus the gain of the omni-greedy choice) and $2\zeta_{t}\left(r\right)$ evaluated at the same partial deployment, in the same intensity units.

Figure 21 compares the deployment performance of directional greedy and omni-greedy on the reduced wildlife instance. Their final objective difference is an empirical comparison between algorithms that may follow different selection sequences. Corollary 7 instead bounds alternative choices evaluated at a common partial deployment and does not directly bound this final difference. The one-step loss is zero at 4 of 6 steps and at most 0.034 intensity units, against allowances between 0.33 and 0.81; the largest loss uses 5% of its allowance. The final DG–OG differences in the left panel are empirical comparisons only: because the two algorithms follow different selection sequences, the one-step corollary does not bound their cumulative difference.

As in the maritime case, the corresponding deterministic void-probability guarantee of Corollary 9 is highly conservative because $S_{M}\left(r\right)$ is dominated by the binary sensing term.

The two datasets therefore exhibit the same qualitative pattern: empirical deployment losses are small relative to the worst-case end-to-end penalties, with the binary sensing term contributing most of the theoretical slack.

### 5.8. Generalization and Computational Performance

The final experiments examine two practical questions: whether deployments estimated from the training trajectory intensity retain performance on held-out trajectories and how computational cost scales with the candidate set. Binary sensing simplifies individual sensing evaluations, whereas omni-greedy additionally reduces the location–orientation search from O(MLK) to O(M(L+K)). The runtime results below focus on this latter reduction.

#### 5.8.1. Maritime

**Figure 22:**
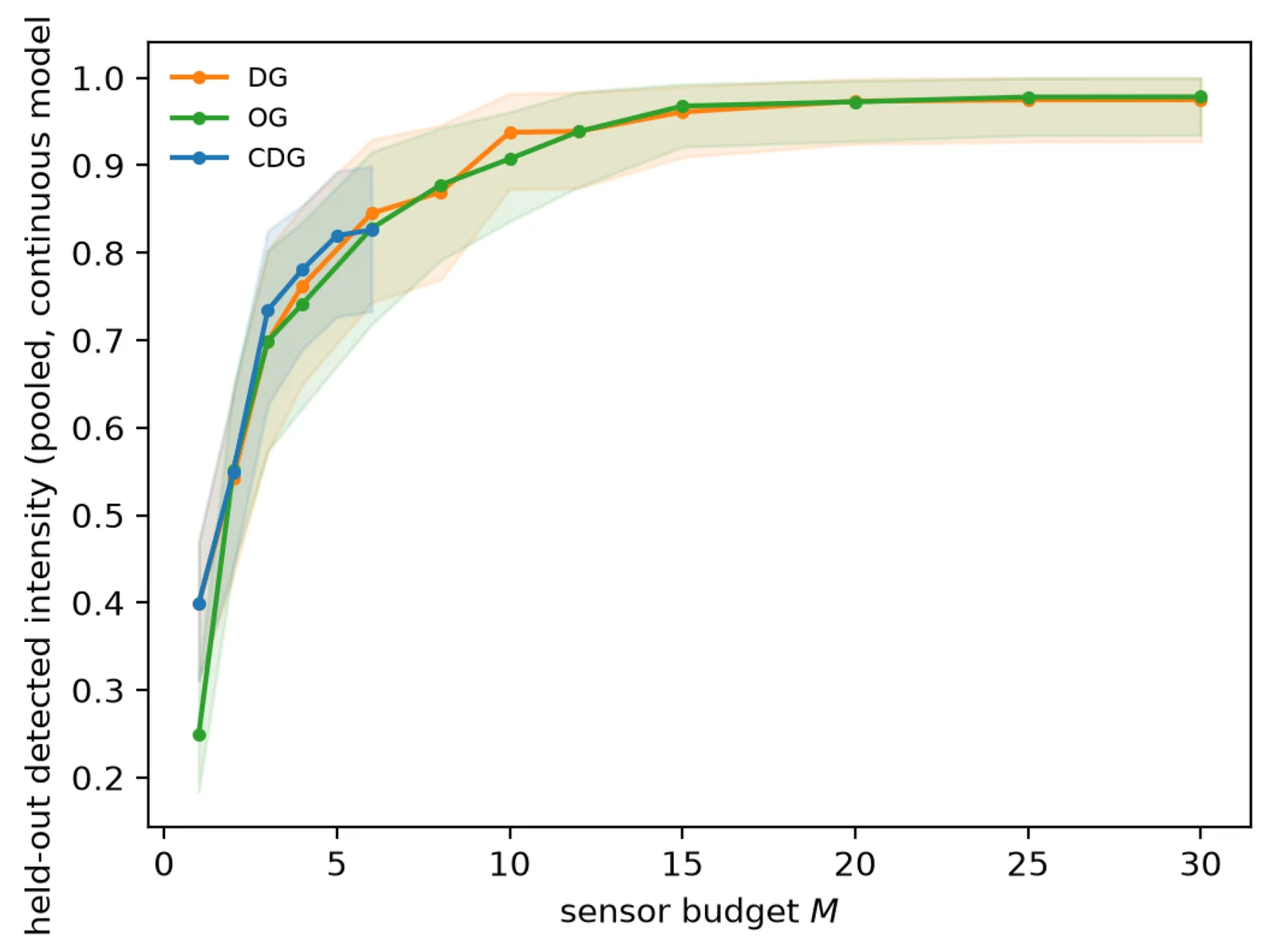
Held-out detection performance with vessel-level bootstrap intervals, Savannah data.

Figure 22 evaluates the deployments on 46 test segments from 25 unseen vessels, with bootstrap resampling performed at the vessel level. All methods are evaluated by the same reference detector. Each test vessel’s segments define its own length-weighted intensity, the held-out score pools detected and total intensity over vessels, and the 95% intervals use the same 1000 vessel-level bootstrap resamples for every method. For binary directional greedy, the pooled held-out score increases from 0.399 at M=1 to 0.975 at M=30; at M=6, it is 0.845 [0.743, 0.929] for DG, 0.826 [0.733, 0.899] for CDG, and 0.828 [0.718, 0.915] for OG. The bootstrap intervals are wider than the performance differences among the informed methods. Thus, the deployments retain high detection performance on vessels excluded from intensity estimation. The overlapping bootstrap intervals do not resolve a clear performance difference among the informed methods.

**Figure 23:**
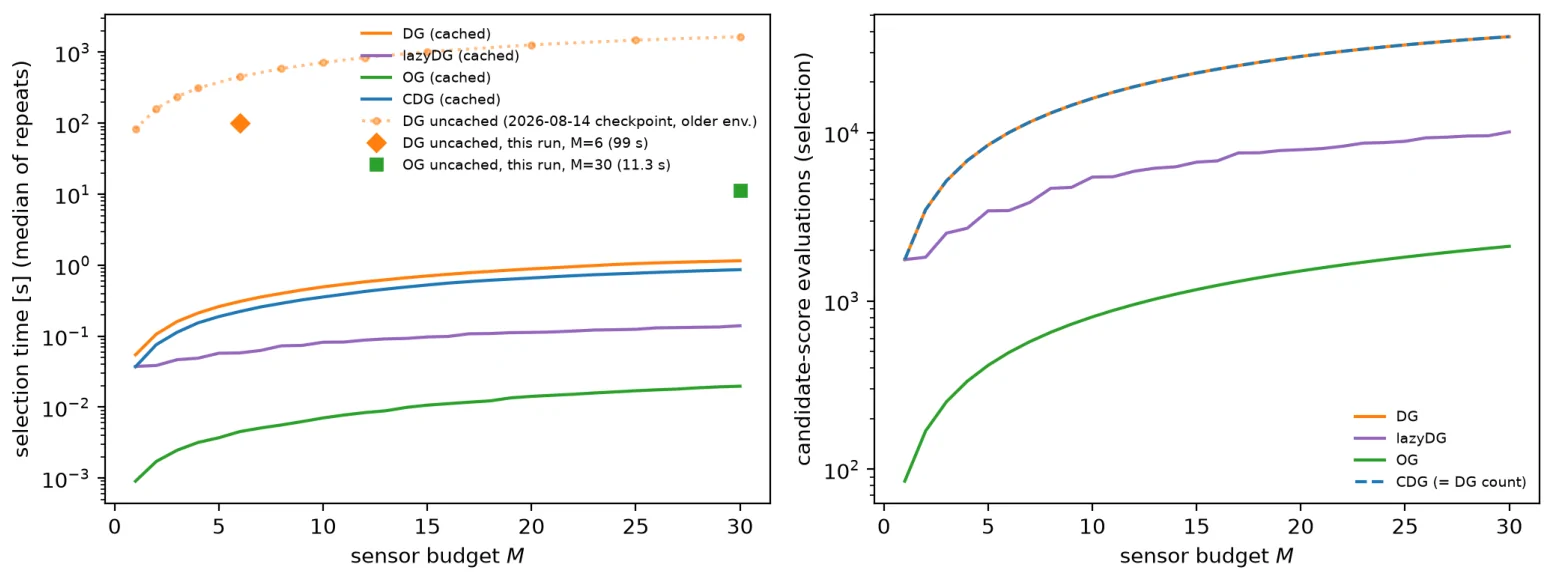
Planning cost for the Savannah data (L=49, K=36). **Left**: greedy-selection time of the cached implementations (median of five repeats) and, for reference, the uncached directional greedy (dotted: single run from an earlier software environment; diamond: re-timed on the same machine as Table 8). **Right**: selection-stage candidate-score evaluations; cached CDG scores the same candidates as DG.

Figure 23 evaluates the computational scaling directly. For L=49 and K=36, the single-step complexity ratio LK/(L+K) predicts a 20.75× reduction in candidate evaluations. The measured ratio is exactly 20.75× at M=1 and decreases to 17.62× at M=30 as location exclusivity progressively reduces the remaining directional candidate set. These counts, and the timings in Figure 23, refer to an implementation that re-evaluates candidate responses at every step.

#### 5.8.2. Cached Implementations and Lazy Directional Greedy

A candidate’s response $\Gamma_{c}\left(x\right)$ or $\Gamma_{c,r}^{B}\left(x\right)$ does not depend on the deployment. All methods can therefore precompute these responses once and, at each step, update only the network residual through $\Pi \left(x;C\cup \left\{c\right\}\right)=\Pi \left(x;C\right)\left[1-\Gamma_{c}\left(x\right)\right]$. We also implement lazy directional greedy [19], which keeps previous marginal gains as upper bounds in a priority queue and re-evaluates only candidates that can still be selected. By Proposition 1, a stale gain bounds the current gain from above; a candidate whose location is occupied is discarded, preserving location exclusivity. Ties within a numerical tolerance are broken by the lowest candidate index after re-evaluating every stale entry in the tolerance band. With this rule, lazy and exhaustive directional greedy returned identical sequences at every budget, and every cached method reproduced the deployments of its uncached counterpart.

Table 8 separates the planning stages, measured on one machine with identical settings. Lazy directional greedy needs 10,122 selection-stage evaluations, 3.7× fewer than exhaustive directional greedy and 4.8× more than omni-greedy. Once responses are cached, selection is no longer the dominant cost: response precomputation and radius calibration are shared by all binary methods, and the total planning times of DG, lazy DG and OG differ by about 1%. The advantage of omni-greedy in the uncached implementation therefore reflects repeated response evaluation rather than an intrinsic difference in total planning cost; with caching, it is confined to the selection stage.

The difference between evaluation-count and wall-clock ratios arises because the two candidate evaluations do not have equal cost. Omnidirectional evaluation uses a disk-intersection test, whereas directional evaluation also checks FOV membership along the trajectory. The evaluation-count reduction is therefore the algorithmic result, while the larger wall-clock speedup includes implementation-specific effects; Table 8 shows that caching removes most of that speedup.

**Figure 24:**
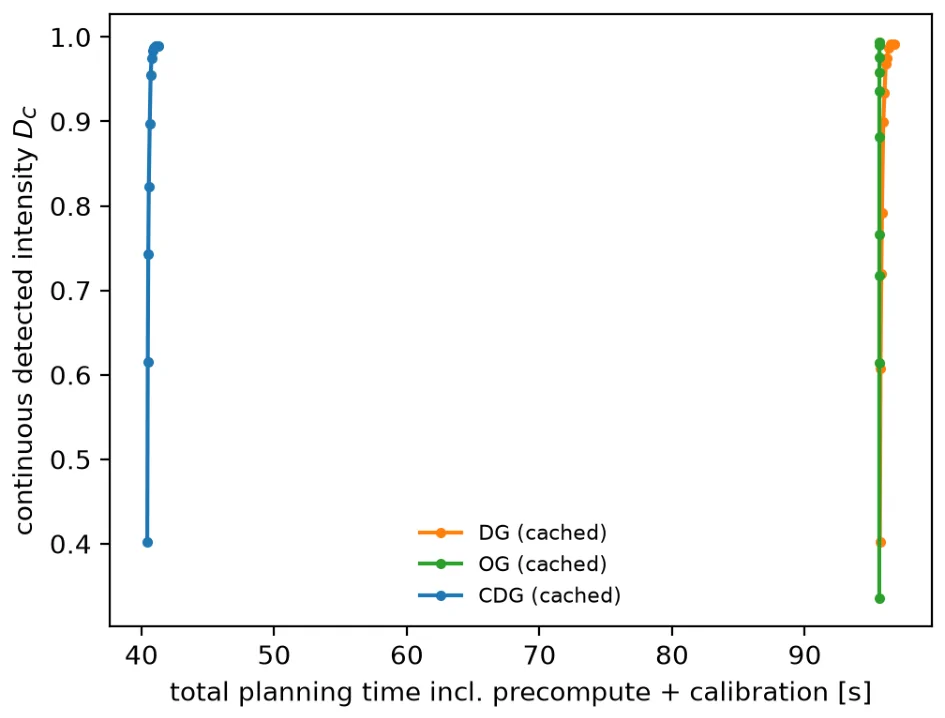
Detection performance versus total planning time (cached implementations), Savannah data.

Figure 24 shows the empirical performance–computation tradeoff directly, using the total planning time of the cached implementations, including shared precomputation and calibration. The binary methods reach similar detection performance at nearly the same total cost; CDG, which needs no binary calibration, is the least costly once its responses are cached.

**Figure 25:**
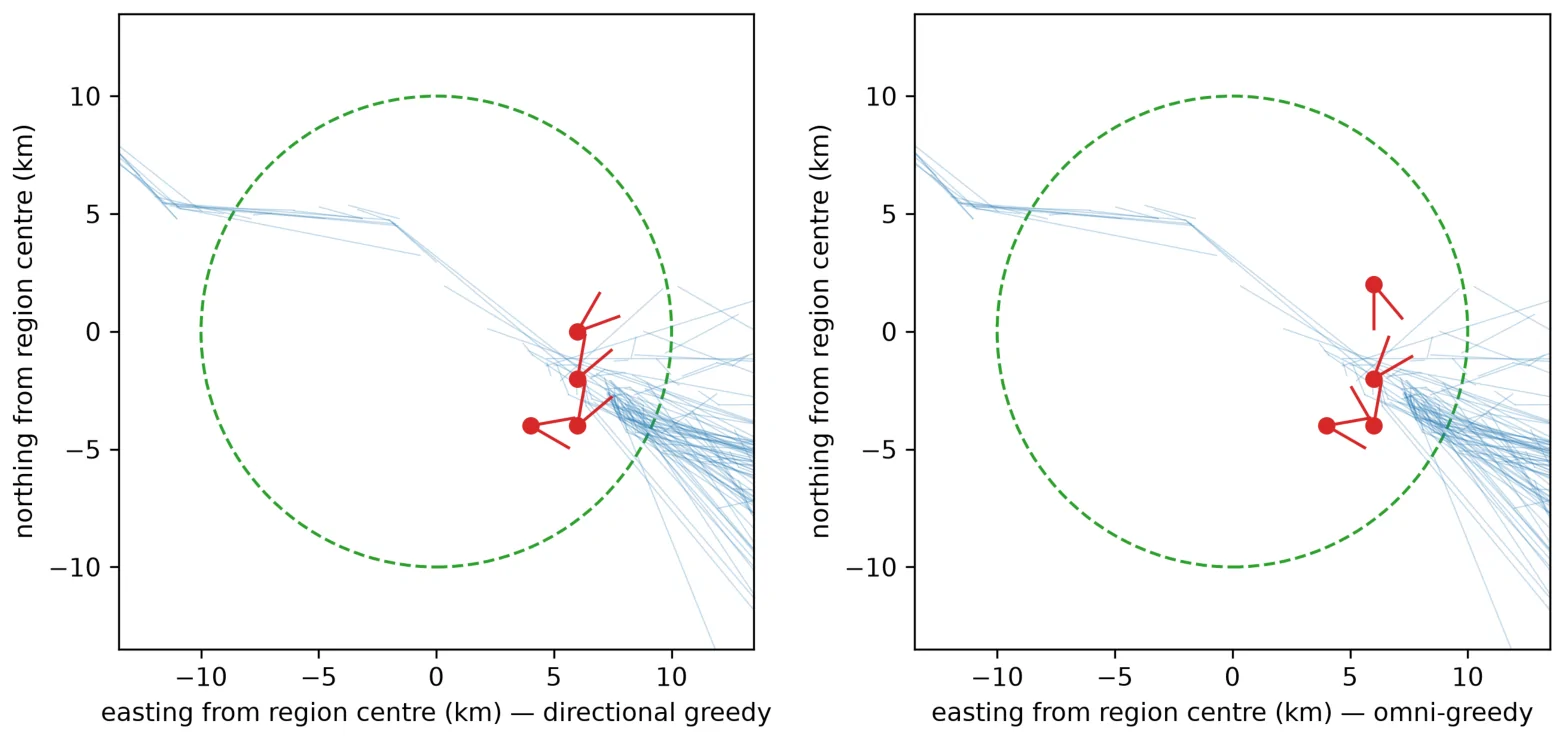
Representative M=4 directional deployments produced by DG and OG, Savannah data.

Figure 25 provides the corresponding geometric interpretation at M=4. Both DG and OG orient sensors toward the dominant channel traffic, while their differences occur mainly among lower-intensity approach regions. Thus, the two algorithms capture the same dominant flow structure despite differing in some individual site choices.

#### 5.8.3. Wildlife

**Figure 26:**
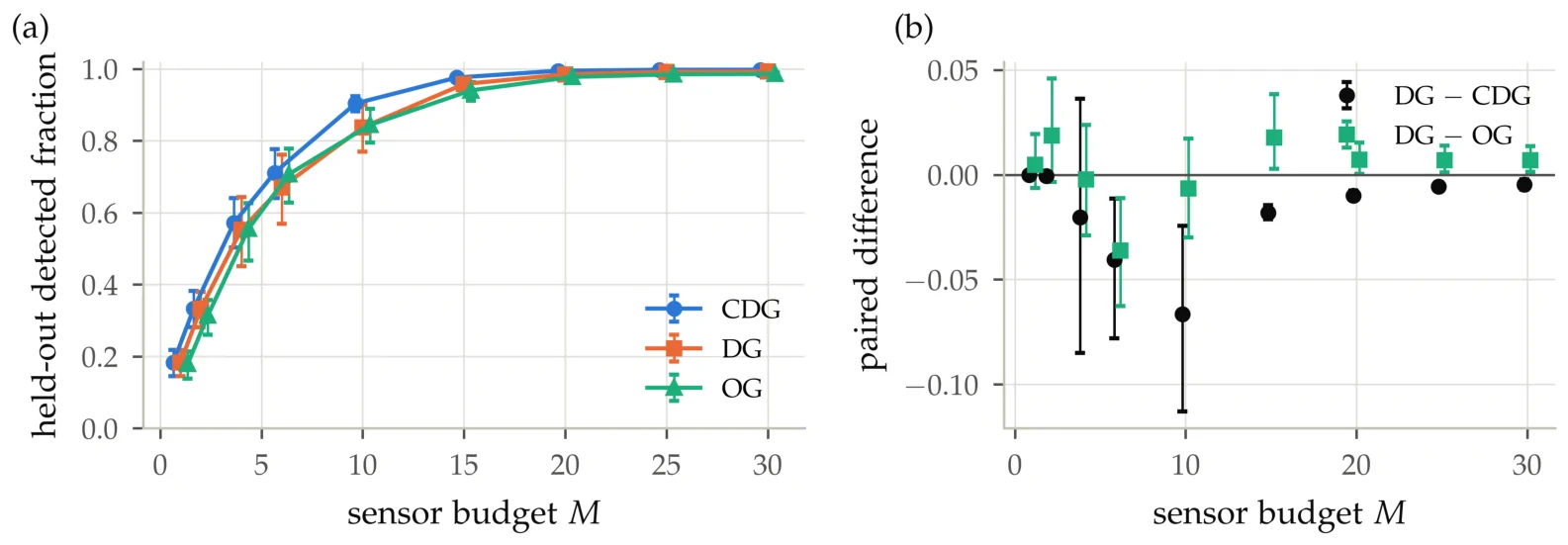
Held-out detection performance with individual-level bootstrap intervals, mule deer data.

Figure 26 evaluates the deployments on 744 test passages from 15 unseen deer, with bootstrap resampling performed at the individual level. All methods are evaluated by the same reference detector. Each test deer’s passages define its own length-weighted intensity, the held-out score pools detected and total intensity over deer, and the 95% intervals use the same 1000 individual-level bootstrap resamples for every method. For binary directional greedy, the pooled held-out score increases from 0.183 at M=1 to 0.993 at M=30; at M=6, it is 0.671 [0.569,0.761] for DG, 0.711 [0.641,0.777] for CDG, and 0.707 [0.627,0.777] for OG. The marginal intervals overlap, but the paired differences on the shared resamples resolve DG−CDG =−0.040 [−0.078,−0.011] and DG−OG =−0.036[−0.063,−0.011], whereas CDG−OG =0.004 [−0.016,0.027] is not resolved at M=6.

**Figure 27:**
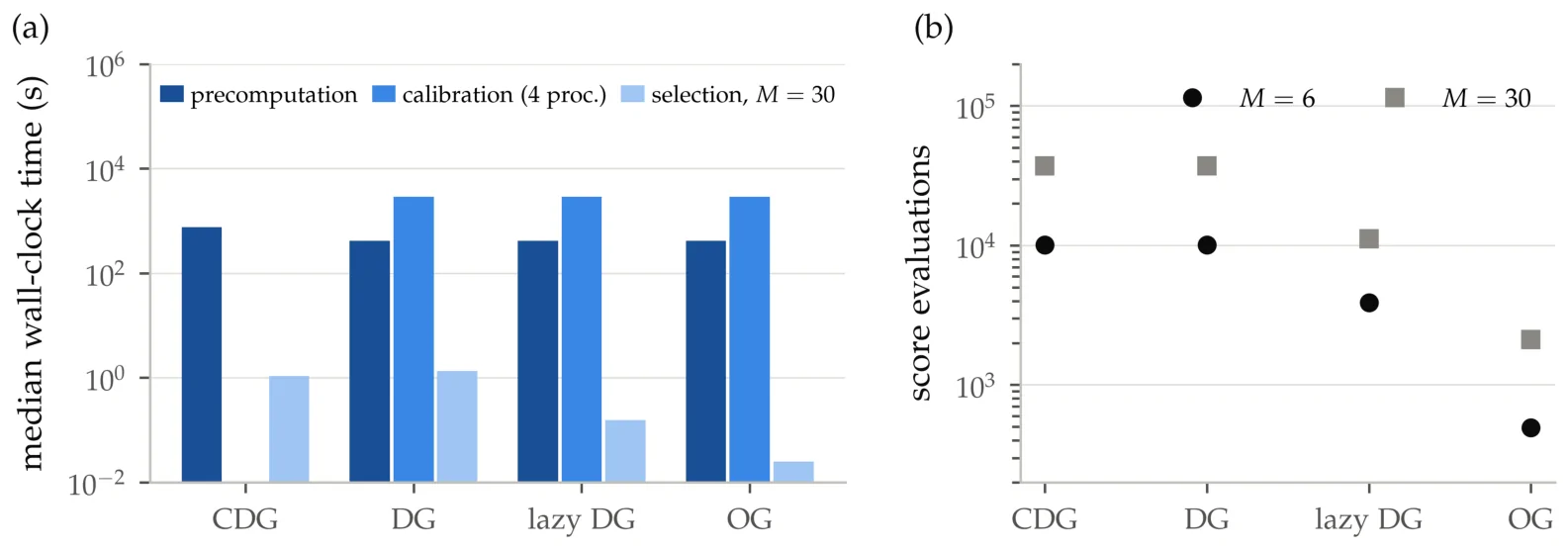
Planning cost for the mule deer data (L=49, K=36). (**a**): median time of each planning stage of the cached implementations. (**b**): selection-stage candidate-score evaluations; cached CDG scores the same candidates as DG.

Figure 27reports the planning cost. For the 49-location, 36-orientation set at M=6, directional greedy performs 10,044 candidate evaluations whereas omni-greedy performs 495, giving a 20.29× reduction. The previously reported 507 also counted 12 calls made inside the omni-greedy loop that do not score candidates: at each of the six steps, one diagnostic evaluation of the scaled omnidirectional score and one update of the network residual. The measured evaluation-count reduction is consistent with the LK/(L+K) ratio implied by the O(MLK) and O(M(L+K)) candidate-evaluation structures. Table 9 separates the planning stages of the cached implementations, measured on one machine with identical settings. Lazy directional greedy returns the same deployments as exhaustive directional greedy with 11,119 selection-stage evaluations at M=30, 3.4× fewer than exhaustive directional greedy and 5.3× more than omni-greedy. Once responses are cached, selection is no longer the dominant cost: response precomputation and radius calibration are shared by all binary methods, and the total planning times of DG, lazy DG and OG are 3305.2, 3304.0 and 3303.9 s. The advantage of omni-greedy is therefore confined to the selection stage.

**Figure 28:**
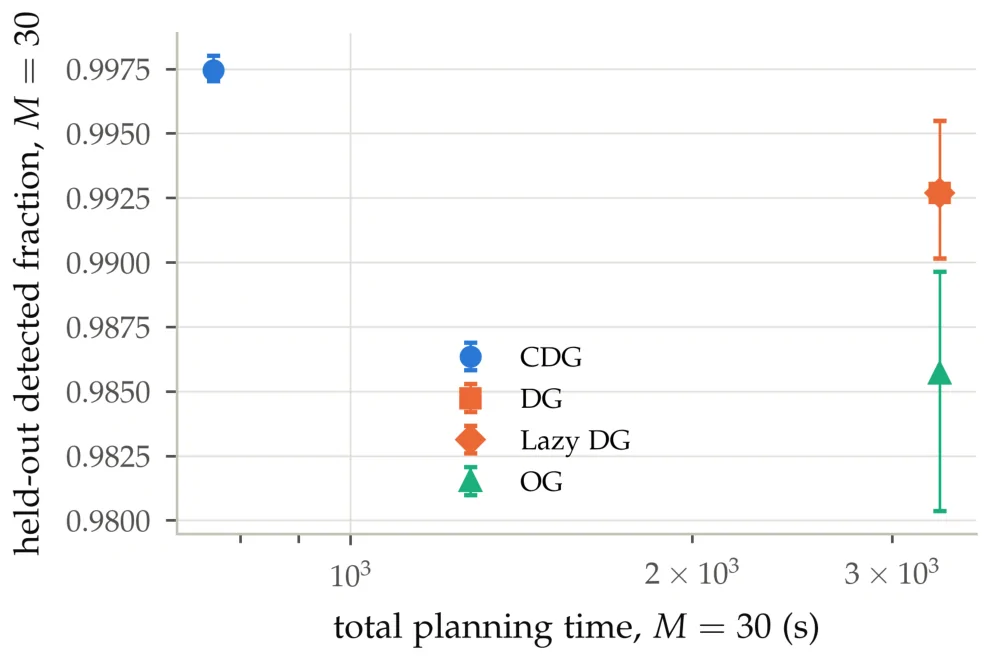
Held-out detection performance versus total planning time (cached implementations), mule deer data.

Figure 28 shows the same empirical performance–computation tradeoff on the wildlife data, using the total planning time of the cached implementations, including shared precomputation and calibration. The binary methods have nearly the same total cost; CDG, which needs no binary calibration, is the least costly once its responses are cached.

**Figure 29:**
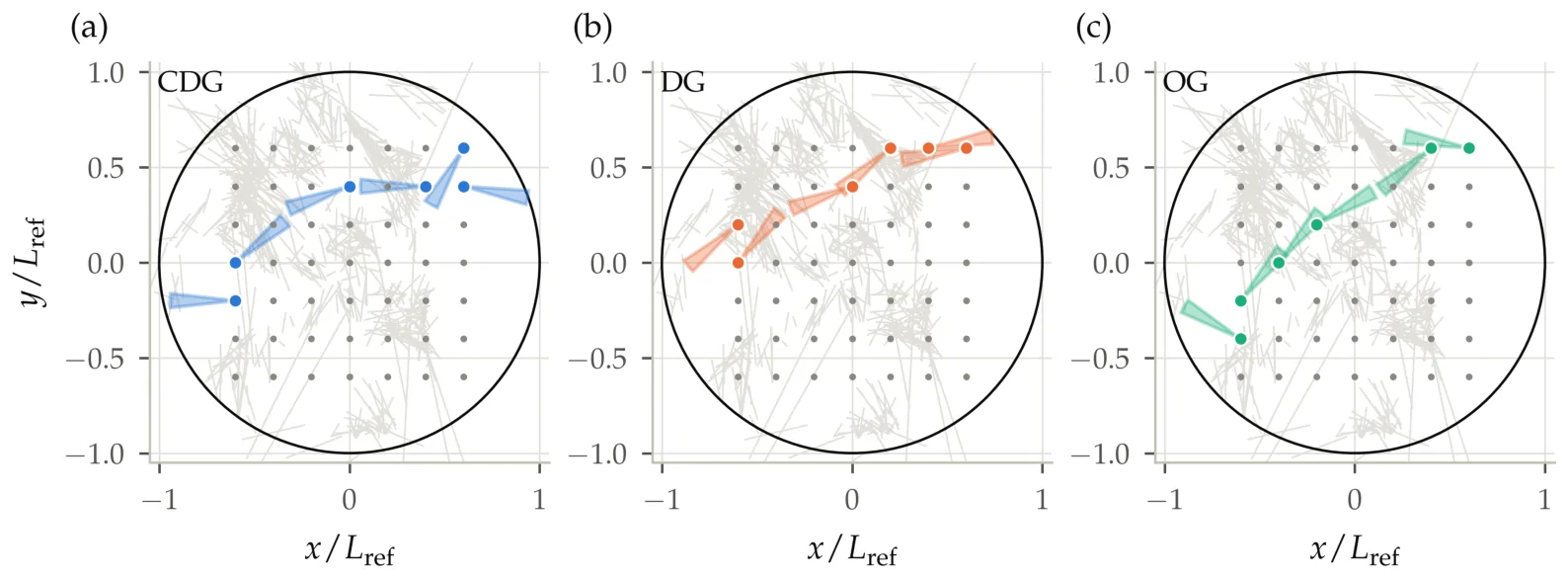
Representative M=6 deployments produced by (**a**) continuous directional greedy, (**b**) binary directional greedy, and (**c**) binary omni-greedy, mule deer data.

Finally, Figure 29 shows that similar performance does not imply similar deployment geometry. At M=6, OG shares only two locations with directional greedy and uses a different orientation at both, yet its continuous detected intensity is 0.019 higher than that achieved by directional greedy. This result is consistent with the one-step omni-greedy analysis: many candidate configurations have similar marginal value, so relatively small ranking perturbations can produce visibly different deployments with limited objective loss.

## 6. Discussion

The two case studies illustrate when sensing and search approximations can support efficient directional deployment and where their limitations remain consequential. The central empirical finding is that different sensor configurations can achieve similar detection performance under the specified probabilistic reference model. This observation supports evaluating the approximations through their effect on deployment benefit, while distinguishing empirical performance from theoretical certification.

### 6.1. Interpreting Sensing Accuracy and Theoretical Certificates

The binary approximation is useful when its dwell-time criterion preserves the relative value of promising sensor configurations. Accurate reproduction of every probabilistic detection response is therefore not necessary for useful deployment decisions. Nevertheless, a small binary-positive error alone does not establish overall accuracy: passages rejected by the binary criterion may still have substantial probabilistic detection benefit.

This distinction explains the gap between the observed deployment performance and the conservative theoretical guarantees. In the reported experiments, the binary sensing term contributes most of the additive penalty in the end-to-end bound. This identifies a limitation of the current certificate, rather than evidence that binary sensing causes the largest empirical loss. A numerically vacuous lower bound cannot certify near-optimal performance, even when a deployment performs well in the experiments. Likewise, agreement with enumerated optima on reduced instances does not establish optimality on the full candidate sets.

The wildlife results show where the binary approximation loses information. A passage whose dwell in a sector is shorter than $\tau_{\min}$ can still receive up to $N_{<}=10$ looks there, and with $\rho_{\max}y\geq 0.50$ inside the horizon *k* such looks detect it with probability at least $1-0.50^{k}$ (0.94 for k=4). Because deer move slowly ($\bar{v}=0.0183$ m/s), such passages are common: they carry a median 19% (interquartile range 17–21%) of a candidate’s reference-detected intensity, and the binary model scores them zero. This is why binary and continuous directional greedy share only their first selection, why binary directional greedy detects 4.0 percentage points less of the held-out traffic than continuous directional greedy at M=6, and why the binary-negative bound is uninformative ($e_{\mathrm{neg}}\geq 0.9$ in both dwell settings). The loss is not a failure of the calibration, since the calibrated radius already equals the sensing horizon; it follows from requiring 10 frames for a confirmed detection when the reference detector needs far fewer at this speed. A geometry-aware binary-negative bound, or a dwell threshold matched to the target speed, would reduce it; the results report the loss rather than tuning $\tau_{\min}$ after the fact.

The practical implication is that radius calibration should be assessed together with reference-model performance and the informativeness of the resulting bounds. Tighter bounds that account for the geometry and sensing opportunities of binary-negative passages are a priority for improving certification.

### 6.2. Deployment Quality and the Cost of Reduced Search

The deployment comparisons in Table 5 and Table 7 show that the consequences of reduced search depend on the sensor budget. At larger budgets, additional sensors can cover passages omitted by earlier selections, and the reported DG–OG performance differences become small. At smaller budgets, the choice of each sensor is more consequential, and the observed differences should not be dismissed as negligible. Applications with limited sensing resources should therefore assess the detection cost of omni-greedy at their intended operating budget.

Different selected locations do not, by themselves, indicate poor approximation. Similar objective values can arise from distinct configurations that cover comparable trajectory intensity. Conversely, similar aggregate detection performance does not make those configurations interchangeable when access, installation cost, or site-specific monitoring requirements matter. Such requirements should be represented explicitly in the deployment problem.

The location-score analysis also shows why directional anisotropy alone is insufficient to assess the omnidirectional proxy. The mismatch between the scaled disk score and the orientation-averaged directional score contributes separately. These quantities describe candidate scores at a common partial deployment; they should not be interpreted as directly bounding the final difference between algorithms following different selection sequences.

The computational comparison concerns reduced candidate scoring relative to exhaustive directional greedy. Its practical value depends on whether the resulting detection loss is acceptable and whether selection cost is substantial relative to preprocessing. Because both probabilistic and binary sensing responses can be cached, runtime comparisons must distinguish response construction, radius calibration, and deployment selection. On the maritime data, lazy directional greedy reduces selection-stage evaluations by 3.7× while returning the same deployments as exhaustive directional greedy, and with cached responses the total planning times of DG, lazy DG and OG differ by about 1% because shared precomputation and radius calibration dominate (Table 8). The reduced candidate scoring of omni-greedy is therefore most useful when selection, rather than preprocessing, dominates the planning cost. The wildlife data give the same picture (Table 9): lazy directional greedy needs 3.4× fewer selection-stage evaluations than exhaustive directional greedy, and the total planning times of DG, lazy DG and OG are 3305, 3304 and 3304 s. On these data, omni-greedy is also not dominated by directional greedy under the reference model: at M=6, it detects 0.770 of the modeled traffic against 0.750 for directional greedy, and 0.707 against 0.671 of the held-out traffic. Both algorithms optimize the binary objective, on which directional greedy is marginally ahead, so the reversal reflects the mismatch between the binary objective and the reference detector rather than an advantage of the reduced search itself.

### 6.3. Scope of Validation and Practical Limitations

The held-out evaluations test transfer to individuals excluded from trajectory-intensity estimation under the same sensing model. They do not constitute field validation of the detector or establish robustness to changes in traffic patterns, habitat use, or sensor conditions. Overlapping uncertainty intervals also do not establish equivalence between methods; a paired analysis of performance differences would be needed to assess that question directly. For the wildlife data, such a paired analysis on the shared individual-level resamples resolves the differences DG−CDG and DG−OG at M=6, whereas CDG−OG is not resolved.

Several modeling choices limit the interpretation of the results. The experiments use a deterministic KDE-based trajectory intensity, so they do not evaluate posterior intensity uncertainty or the Jensen gap associated with the LGCP formulation. In the wildlife study, sparse GPS observations leave intermediate paths and residence times unresolved. The trajectory reconstruction therefore introduces an additional source of uncertainty that is not included in the sensing- and search-approximation bounds. Two consequences are quantified. First, detection is evaluated at interpolated positions: on the traced passage, every one of the sensing instants in the sensor footprint falls inside a single 5 h fix interval (Figure 5). Second, each passage is represented by its directed line, so detection is credited on the extension of the observed segment: for held-out passages that cross a deployed sector, on average, only 5.8% of the detection chord lies within the observed segment. Straight-line speeds between sparse fixes also underestimate travel speed [15], which lengthens modeled dwell times. The wildlife results should therefore be read as deployment comparisons for reconstructed straight passages at the modeled speed. Restricting each passage to its observed extent, or assigning speed as a trajectory mark, are modeling changes that were identified but not adopted in this revision. Evaluation on held-out individuals retains this reconstruction assumption and does not independently validate its accuracy.

Detection performance is conditional on the specified radial response, sampling convention, effective sensing horizon, and independence assumptions. Radius calibration against that reference model does not establish the accuracy of the physical detector. Field application would require sensor-specific validation and sensitivity analysis, together with candidate locations that account for terrain, access, occlusion, and other siting restrictions.

Further evaluation should therefore prioritize larger-scale accelerated-search benchmarks beyond the cached and lazy variants compared here, sensitivity to motion and sensing assumptions, and validation with measured detection outcomes. These additions would clarify whether the observed computational tradeoffs persist under more realistic deployment conditions.

The numerical evaluation uses a phase-free approximation to the periodically scheduled sensing model; accordingly, the theoretical sample-count bounds characterize the scheduled model and are not formal certificates for the numerical results.

## 7. Conclusions

This study developed a theoretically grounded and scalable framework for joint placement and orientation of directional sensors for stochastic directed-trajectory detection. The framework combines (i) trajectory-level probabilistic sensing with an error-controlled binary approximation and (ii) an omni-greedy strategy that reduces candidate-evaluation complexity from O(MLK) to O(M(L+K)). Theoretical results quantify binary sensing distortion, provide principled radius calibration and decision-preservation conditions, characterize location–orientation decoupling, and establish an end-to-end deployment guarantee. Experiments using real maritime AIS and wildlife GPS trajectories show that the binary approximation can preserve high-value greedy decisions despite conservative worst-case bounds, while omni-greedy substantially reduces candidate evaluations with relatively small losses in detection performance compared with directional greedy search; with cached candidate responses, however, total planning time is dominated by shared precomputation and radius calibration. Overall, the results show how binary sensing can provide analytical control of approximation error while location–orientation decoupling enables scalable directional sensor deployment. 

## Figures and Tables

**Table 1:** Comparison of representative related formulations. The table summarizes modeling and algorithmic characteristics, rather than results from a common experimental benchmark. MATE is a benchmark environment rather than a single deployment algorithm.

Study	Objective and Target Model	Decisions and Approach	Relationship to This Study
Fusco and Gupta [1]	Geometric coverage with directional sensing	Sensor selection and orientation; approximation algorithms	Foundation for orientation-aware selection; geometric coverage differs from accumulated passage detection
Wasim et al. [2]	Mobile-target detection using probabilistic motion models	Time-phased directional deployment; direct-search optimization	Accounts for motion and deployment timing; motivates comparison with passage-based deployment objectives
Xu et al. [3]	Coverage of moving targets	Hierarchical reinforcement learning for target assignment and sensor orientation control	Online coordination rather than selection of a fixed deployment
Pan et al. [4]	Distributed coverage of mobile targets	Benchmark for multi-agent camera control, communication, and competitive target behavior	Evaluates sequential control policies rather than static placement algorithms
Kim et al. [6]	Detection of Poisson-distributed targets; void-probability objective	Sensor placement through a submodular surrogate	Foundation for stochastic detection objectives and approximation analysis
Kósa et al. [5]	Moving-target coverage while maintaining *k*-barrier coverage	Sector selection at given camera locations; integer programming, clustering, and greedy methods	Explicit geometric barrier constraint differs from the intensity-weighted passage objective
Kim et al. [8]	Detection of stochastic linear trajectories	Placement in a transformed trajectory space	Direct precursor to trajectory-based deployment modeling
Kim et al. [7]	Void probability under target-intensity uncertainty	Improved network-performance approximation for seabed sensors	Addresses objective approximation under occurrence uncertainty
Present study	Intensity-weighted detection of finite target passages	Joint location and orientation selection through a binary sensing model and omni-greedy procedure	Separates sensing-approximation error from

**Table 2:** Principal notation used in the deployment formulation and algorithms.

Symbol	Definition
*Deployment domain and decisions*	
Q,Ψ	Physical monitoring region and admissible sensor-location region, respectively.
L,B	Finite sets of candidate locations and sensor orientations.
L,K,M	Numbers of candidate locations and orientations, and sensor budget, respectively.
c=(a,β)	Sensor configuration comprising location *a* and boresight orientation β.
V=L×B	Set of candidate sensor configurations.
$C,\;I_{M}$	Selected deployment and family of feasible deployments satisfying the budget and location-exclusivity constraints.
L(C)	Set of physical locations occupied by deployment *C*.
*Trajectory representation and occurrence*	
x=(ξ,p),Θ	Directed-line representation, comprising heading ξ and signed offset *p*, and the relevant trajectory domain.
$s_{x}\left(\tau \right),\;v_{x},\;I_{x}$	Target position at time τ, passage speed, and finite passage interval for trajectory *x*.
$\lambda \left(x\right),\;\bar{\lambda }\left(x\right)$	Random trajectory intensity and the deterministic intensity used for planning; the latter is a posterior mean when an LGCP is fitted.
$\Lambda_{0}$	Expected total number of passages in the specified observation window.
*Sensing model*	
$\phi ,\;R_{\mathrm{sens}}$	Sensor half-FOV angle and effective sensing horizon.
$\rho_{\max},\;y\left(d;l\right)$	Maximum instantaneous detection probability and radial response at distance *d*, with distance-scale parameter *ℓ*.
$f,\;\vartheta_{i}$	Sampling frequency and sampling phase of sensor *i*.
$F_{i},\;F_{i}\left(r\right)$	Directional FOV and its restriction to binary radius *r*.
$N_{i}\left(x\right),\;\gamma_{i}\left(s\right),\;\Gamma_{i}\left(x\right)$	Number of sensing opportunities, instantaneous detection probability, and trajectory-level detection probability.
$r,\;\tau_{\min},\;T_{i}\left(x;r\right)$	Binary sensing radius, minimum required dwell time, and passage duration inside $F_{i}\left(r\right)$.
$\Gamma_{i,r}^{B}\left(x\right)$	Binary directional detection indicator based on the dwell-time criterion.
$\Gamma_{a,r}^{O}\left(x\right)$	Binary omnidirectional detection indicator used to score location *a*.
*Network objective and selection scores*	
$\Pi \left(x;C\right),\;\Pi_{r}^{B}\left(x;C\right)$	Network missed-detection probability and its binary counterpart.
$\tilde{\Lambda }\left(C\right),\;\Lambda_{r}^{B}\left(C\right)$	Expected missed-trajectory count under the probabilistic reference model and binary surrogate.
$F\left(C\right),\;F_{r}^{B}\left(C\right)$	Expected detected-trajectory count under the reference model and binary surrogate.
$\nu \left(C\right),\;\tilde{\nu }\left(C\right)$	Posterior-averaged LGCP void probability and deterministic-intensity void probability, respectively; the latter is a lower bound on the former when $\bar{\lambda }$ is the LGCP posterior mean.
$\Delta \left(c|C\right),\;\Delta_{r}^{B}\left(c|C\right)$	Additional detection benefit from configuration *c* under the reference and binary models.
$\Delta_{r}^{O}\left(a|C\right)$	Omnidirectional location score evaluated against the current directional residual.
*Finite numerical implementation*	
$J,\;x_{j},\;w_{j}$	Number of trajectory quadrature nodes, node *j*, and its nonnegative intensity weight.
$B_{j,a,k},\;O_{j,a}$	Precomputed binary responses for directional configuration $\left(a,\beta^{\left(k\right)}\right)$ and omnidirectional location *a*.
$z_{j}$	Current binary network missed-detection indicator at node $x_{j}$.

**Table 3:** Experimental setup for the maritime and wildlife case studies. Normalized lengths are expressed relative to the monitoring-region radius $L_{\mathrm{ref}}$. The sensing parameters describe the numerical models used in the experiments.

Quantity	Maritime	Wildlife
Trajectory data	Vessel AIS	Mule deer GPS
Monitoring-region radius $L_{\mathrm{ref}}$	10 km	6 km
Retained trajectory segments	190	2398
Training/testing split unit	Vessel	Individual deer
Training/testing individuals	57/25	36/15
Trajectory intensity estimator	Periodic KDE	Periodic KDE
Candidate locations *L*	49	49
Candidate orientations *K*	36	36
Maximum reported sensor budget	30	30
Sensor half-FOV angle ϕ	$20^{\circ }$	$6.26^{\circ }$
Sampling interval 1/f	1 s	600 s
Maximum instantaneous detection probability $\rho_{\max}$	0.90	0.90
Effective sensing horizon $R_{\mathrm{sens}}$	2.4 km	2.052 km
Minimum dwell time $\tau_{\min}$	15 s	6000 s
Dwell-based count $\left\lfloor f\tau_{\min}\right\rfloor$	15	10
Short-dwell upper count $N_{<}=\left\lceil f\tau_{\min}\right\rceil$	15	10
Radial kernel y(d;l)	$\exp(-d^{2}/l)$, *d* in units of $L_{\mathrm{ref}}$ (A)	$\exp(-d^{2}/l)$, *d* in units of $L_{\mathrm{ref}}$ (A)
Kernel scale *ℓ*	0.01993, from $\rho_{\max}y\left(R_{\mathrm{sens}};l\right)=0.05$ (A)	0.50 →0.199 $L_{\mathrm{ref}}^{2}$, from $\rho_{\max}y\left(R_{\mathrm{sens}};l\right)=0.5$ at the Johnson detection range (S)
Speed used for dwell time	5.56 m/s, median training speed, all passages (D)	0.0183 m/s, median training speed, all passages (D)
Quadrature: heading × offset nodes; points per chord	120×81; 64	120×81; 512
KDE bandwidths $\left(\xi ,\tilde{p}\right)$; weights	(0.18rad,0.06); segment length (A)	(0.30rad,0.40), cross-validated over individuals; segment length (D)
Outer-region count $N_{\max}\left(r^{★}\right)$, evaluated set	295	0 ($r^{★}=\tilde{R}$)
Calibrated binary radius $r^{★}/L_{\mathrm{ref}}$	0.1872	0.342
Sensor budget used for radius calibration	30	6
Observation window for void probability	24 h	14 days

**Table 4:** Parameters of the wildlife study. (D) estimated from the training data; (S) sensor specification; (A) assumption; (N) numerical resolution fixed by a convergence study. Lengths are also given normalized by $L_{\mathrm{ref}}=6$ km.

Parameter	Value		Reason/Reference
*Trajectory data*			
GPS fix interval	5 h	D	96.7% of inter-fix intervals [9].
Stationary-step threshold	60 m	A	GPS-collar location errors are of the order of tens of meters [14]; a displacement below 60 m in 5 h cannot be distinguished from a stationary animal and carries no heading.
Trip gap	15 h	A	Three fix intervals: up to two missed fixes are bridged; longer gaps start a new trip.
Linear-segment rule	$20^{\circ }$, ≥3 fixes, straightness ≥0.98	A	Same rule as the maritime study, so both use one segmentation.
Retained passages	2398	D	Observed segment intersects the monitoring disk, so every retained passage physically crosses the region.
Region center and radius	max. overlap; $L_{\mathrm{ref}}=6$ km	D/A	Center: point where 53 of 54 individuals have fixes within 6 km (D). Radius: corridor-scale design choice (A).
Train/test split	36/15 individuals	–	70/30 by individual with a fixed seed, so no animal contributes to both sets.
Target speed $\bar{v}$	0.0183 m/s	D	Median endpoint speed of the training passages (quartiles 0.012–0.028 m/s). Straight-line speed between sparse fixes underestimates travel speed [15], so dwell times are, if anything, overestimated.
KDE bandwidths	0.30 rad; 0.40 (2.4 km)	D	Likelihood cross-validation [16] with folds formed by individuals, matching the held-out design.
Expected passages *T*	2.71 per 14 days	D	1654 training passages over 8541 individual-days =0.194 per individual-day, times a 14-day window (A).
*Sensor*			
Half field of view ϕ	$6.26^{\circ }$	S	$\arctan(N_{h}p/2f_{\mathrm{lens}})$ with 640 pixels, 12μm pitch and a 35 mm lens [10].
Sensing horizon $R_{\mathrm{sens}}$	2052 m (0.342)	S	Johnson detection criterion: two pixels across the critical dimension $\sqrt{1.10\times 1.80}$ m of a deer-sized target [11].
Maximum single-look probability $\rho_{\max}$	0.90	A	No detector performance data; same value as the maritime study.
Distance scale *ℓ*	$0.199\;L_{\mathrm{ref}}^{2}$ (2.68 km)	S	$\rho_{\max}y\left(R_{\mathrm{sens}};l\right)=0.5$, the probability at the Johnson detection range that defines $R_{\mathrm{sens}}$ [11,12]: $l={\tilde{R}}^{2}/\ln\left(2\rho_{\max}\right)$. A fit to the Johnson-criteria target transfer probability function gives 0.214 [13].
Revisit interval 1/f	600 s	A	Revisit and processing interval of the modeled installation; much shorter than the 5 h fix interval.
Dwell threshold $\tau_{\min}$	6000 s	A	$N_{\min}=\left\lfloor f\tau_{\min}\right\rfloor =10$ frames for a confirmed detection; $N_{<}=\left\lceil f\tau_{\min}\right\rceil =10$; at $\bar{v}$ it requires 110 m of path in the sector.
*Numerical*			
Θ grid	120×81	N	$\Delta \xi =3^{\circ }$, $\Delta \tilde{p}=0.025$ (148 m); refining to 240×161 changes *D* by less than 0.004 (Figure 3).
Points per chord	512	N	64 points are not converged for deer (Section 5, Figure 3).
Candidate set	L=49, K=36	A	7×7 grid with 1.2 km spacing; $10^{\circ }$ orientation spacing, below the $12.5^{\circ }$ field of view.
Bootstrap	1000 resamples	–	Resampling of whole individuals, shared by all methods [17].

**Table 5:** Deployment performance for the Savannah maritime case study. DG, OG, and CDG denote binary directional greedy, binary omni-greedy, and continuous directional greedy, respectively. $D_{B}$ and $D_{C}$ are normalized detected intensities under the binary and probabilistic reference models. The same selected deployments are evaluated under each model. $\tilde{\nu }$ is the deterministic-intensity void probability over a 24 h observation window. Dashes indicate budgets for which CDG was not evaluated.

	$D_{B}\left(C\right)$			$D_{C}\left(C\right)$			$\tilde{\nu }\left(C\right)$	
M	DG	OG	Random	DG	OG	CDG	DG	OG
1	0.2964	0.2829	0.1056	0.4018	0.3351	0.4018	0.0495	0.0354
2	0.5366	0.5415	0.1970	0.6069	0.6144	0.6149	0.1388	0.1442
4	0.7388	0.7285	0.3551	0.7917	0.7664	0.8229	0.3511	0.3092
6	0.8470	0.8400	0.4791	0.8994	0.8814	0.8969	0.6033	0.5512
10	0.9396	0.9291	0.6668	0.9685	0.9584	—	0.8538	0.8115
15	0.9748	0.9726	0.7884	0.9873	0.9907	—	0.9382	0.9541
20	0.9828	0.9832	0.8762	0.9909	0.9929	—	0.9555	0.9650
25	0.9841	0.9859	0.9157	0.9918	0.9940	—	0.9594	0.9704
30	0.9841	0.9861	0.9403	0.9918	0.9941	—	0.9594	0.9706

**Table 6:** Agreement between the saved binary directional greedy (DG) and probabilistic directional greedy (CDG) deployments, Savannah data. Configurations are location–orientation pairs; the selection order also differs from M=2 onward.

*M*	Identical Set	Shared Configurations	Shared Locations
1	yes	1/1	1/1
2	no	1/2	1/2
3	no	1/3	1/3
4	no	1/4	1/4
5	no	2/5	3/5
6	no	3/6	4/6

**Table 7:** Deployment performance for the mule deer case study. DG, OG, and CDG denote binary directional greedy, binary omni-greedy, and continuous directional greedy, respectively. $D_{B}$ and $D_{C}$ are normalized detected intensities under the binary and probabilistic reference models. The same selected deployments are evaluated under each model. $\tilde{\nu }$ is the deterministic-intensity void probability over a 14-day observation window. CDG is evaluated at every budget.

	$D_{B}\left(C\right)$			$D_{C}\left(C\right)$			$\tilde{\nu }\left(C\right)$	
M	DG	OG	Random	DG	OG	CDG	DG	OG
1	0.1622	0.1596	0.1143	0.2033	0.1995	0.2033	0.1153	0.1141
2	0.2999	0.3042	0.2089	0.3685	0.3487	0.3687	0.1805	0.1711
4	0.5409	0.5471	0.3777	0.6196	0.6136	0.6259	0.3565	0.3508
6	0.6962	0.6932	0.5009	0.7505	0.7697	0.7733	0.5084	0.5356
10	0.8611	0.8364	0.6758	0.8992	0.8885	0.9206	0.7610	0.7392
15	0.9495	0.9320	0.7854	0.9657	0.9600	0.9819	0.9112	0.8972
20	0.9797	0.9761	0.8635	0.9856	0.9843	0.9957	0.9616	0.9584
25	0.9886	0.9860	0.9031	0.9902	0.9882	0.9973	0.9738	0.9685
30	0.9901	0.9878	0.9274	0.9908	0.9885	0.9973	0.9753	0.9692

**Table 8:** Planning cost by stage, Savannah data (L=49, K=36, M=30). Medians of repeated runs on one machine (x86_64, 16 logical CPUs, Python 3.12, NumPy 2.5); shared stages are counted once per method. Precomputation (binary) n=5, IQR [23.4, 23.7] s; calibration n=2, [71.9, 72.3] s; selection n=5. Uncached reference on the same machine: DG to M=6, 99.5 s; OG to M=30, including diagnostics 11.3 s.

Stage	DG	Lazy DG	OG	CDG
Response precomputation [s]	23.5	23.5	23.5	40.4
Radius calibration [s]	72.1	72.1	72.1	–
Greedy selection [s]	1.15	0.140	0.020	0.86
Optional certification [s]	–	–	0.023	–
Total planning time [s]	96.8	95.7	95.6	41.3
Selection evaluations	37,260	10,122	2115	37,260

**Table 9:** Planning cost by stage, mule deer data (L=49, K=36, M=30; 512 points per chord). Medians of repeated runs on one machine (4 logical CPUs, Python 3.11, NumPy 2.4); shared stages are counted once per method. Precomputation (binary) n=5, IQR [404.5, 414.2] s; calibration (24 radii) n=2, [2844.7, 2935.3] s; selection n=5. Uncached reference on the same machine (64 points per chord): DG to M=6, 182.2 s; OG to M=30, including diagnostics 21.1 s. The cached selections reproduce the saved DG and OG sequences, and lazy DG returns the DG sequence; the cached CDG agrees with the saved one through M=26 and then breaks a tie between marginal gains that are equal to within $1.1\times 10^{-14}$ expected passages differently; the final detected fractions agree to within $4.7\times 10^{-13}$.

Stage	DG	Lazy DG	OG	CDG
Response precomputation [s]	413.8	413.8	413.9	756.1
Radius calibration [s]	2890.0	2890.0	2890.0	–
Greedy selection [s]	1.344	0.157	0.025	1.089
Optional certification [s]	–	–	0.021	–
Total planning time [s]	3305.2	3304.0	3303.9	757.2
Selection evaluations	37,260	11,119	2115	37,260

## Data Availability

The AIS vessel data analyzed in this study are openly available through MarineCadastre AccessAIS [18]. The mule deer GPS telemetry data are openly available as described in [9].

## Abbreviations

The following abbreviations are used in this manuscript:

AIS	Automatic Identification System
FOV	Field of View
GPS	Global Positioning System
LGCP	Log-Gaussian Cox Process
